# Supplementary material for: Engineering of a Peptide α‐N‐Methyltransferase to Methylate Non‐Proteinogenic Amino Acids
Source: Angew Chem Weinheim Bergstr Ger. 2021 May 17;133(26):14440–4. doi: 10.1002/ange.202100818 (PMC10947093; doi:10.1002/ange.202100818)
Supplement: Supplementary file 1 — Supplementary [file ANGE-133-14440-s001.pdf]

Angewandte Chemie
Eine Zeitschrift der Gesellschaft Deutscher Chemiker

Supporting Information

Engineering of a Peptide α-N-Methyltransferase to Methylate Non-Proteinogenic Amino Acids

*Haigang Song, Antony J. Burton, Sally L. Shirran, Jūratė Fahrig-Kamarauskaitė, Hannelore Kaspar, Tom W. Muir, Markus Künzler, and James H. Naismith**

ange_202100818_sm_miscellaneous_information.pdf

Table of Contents

Experimental Procedures *3*
Results and Discussion *5*
References *96*

Experimental Procedures

Materials

Tris buffer, 2-Mercaptoethanesulfonic acid sodium salt (MESNA), ethylenediaminetetraacetic acid (EDTA), deoxyribonuclease I (DNase) from bovine pancreas, nickel (II) sulfate and imidazole were purchased from Sigma-Aldrich. cOmplete EDTA-free protease inhibitor cocktail was purchased from Roche. Oligos were purchased from Integrated DNA Technologies. Restriction enzymes including NdeI and KpnI, DNA polymerase (Phusion), *E. coli* competent cells Bl21 (DE3) and DH5a were purchased from New England Biolabs. Tris(2-carboxyethyl)phosphine hydrochloride (TCEP) was purchased from Fluorochem. Glycerol was bought from VWR Chemicals. Isopropyl β-d-1-thiogalactopyranoside (IPTG) and kanamycin monophosphate were purchased from Generon. 5 ml His-Trap FF nickel column, HiPrep 26/10 desalting column, and HiLoad Superdex S200 column were purchased from GE healthcare. Terrific Broth media was purchased from Melford. Peptides were either purchased from Bio-synthesis or GenScript. DNA sequencing was done by Source Bioscience.

Protein cloning, expression and purification.

Deletion variant of OphMA (OphMAΔC12, OphMAΔC12-G390_E391insCys, and fOphMA2ΔC12-G390_E391insCys) were carried out using a published protocol [1] with Phusion DNA Polymerase and corresponding templates.

The N-terminal region of fused *OphMA* gene including the flexible (GGGGS)6 linker was synthesized by GenScript with NdeI and KpnI restriction site at the 5' and 3' respectively. The DNA sequence was codon optimized for *E. coli* and contained the sequence coding for the 8His-tagged OphMA followed by the (SerGly4)6 linker and the N-terminal part of the second OphMA up to residue Thr17. The restriction endonuclease sites NdeI and KpnI were introduced at the 5' and the 3' ends of the synthetic gene for cloning. The second KpnI site that originally was in the 5' terminal OphMA gene was removed. The sites NheI and PstI were installed upstream and downstream of the sequence coding for the (GGGGS)6 linker to allow for easy linker exchange. To construct the full-length OphMA tandem gene the NdeI/KpnI fragment of the synthetic gene was ligated to OphMA.

For protein expression, plasmids were transformed into *E. Coli* BL21 (DE3) cells. Single colonies were innoculated at 37°C overnight in LB medium before transferred into Terrific Broth medium. The cultures were incubated at 37 ºC until OD600 (optical density at 600 nm) reached around 1.0 before cooled down in ice-cold water bath. Protein expression was induced with 0.2 mM isopropyl-β-D-thiogalactopyranoside (IPTG) for 20 hours before cells were collected and frozen at -80 ºC. For purification, frozen cells were resuspended in lysis buffer [25 mM tris-HCl (pH 8.0), 300 mM NaCl, 10% glycerol, DNase I (25 µg/ml; Sigma), and EDTA-free cocktail protease inhibitor (Roche Applied Science)] for 1 hour in a cold room. Cells were then lysed using a cell disrupter at 30 kpsi (Constant System Ltd.), and the cell lysate was cleared by centrifugation (40,000g for 30 min) at 4°C. The supernatant was filtered through 0.45 µm membrane filter (MF-Millipore), loaded on a 5 ml nickel HisTrap FF column (GE Healthcare), washed with lysis buffer, and eluted with 250 mM imidazole. Protein fractions were collected and desalted into buffer (50 mM Tris, pH 8.0, 100 mM NaCl, and 10% glycerol and 1 mM TCEP). For the purification of OphMA-ΔC12, OphMA-ΔC12- G390_E391insCys, and OphMAΔC27-CfaN-SUMO, the histags at the N-terminus were removed by incubation with TEV protease. Concentrated protein was further purified using size-exclusion chromatography (Superdex S200, GE Healthcare) pre-equilibrated with buffer (50 mM Tris pH 8.0, 100 mM NaCl, 10% glycerol and 2 mM TCEP). Well-folded proteins were collected, concentrated, aliquoted, flash-frozen in liquid nitrogen and stored at -80 ºC for later use. Protein purity was verified using SDS-Page gel (Invitrogen, NuPAGE™ 4 to 12%).

Thermal shift assay

Thermal shift assay was performed on a Prometheus NT.48. Proteins at a concentration of 0.5 mg/ml in 50 mM Tris pH 8.0, 100 mM NaCl, 10% glycerol and 2 mM TCEP were loaded into a capillary. The capillaries containing protein samples were placed in the chamber pre-cooled to 15 ºC. The aggregation process starts with a linear increase of temperature by 1 ºC/min from 15 ºC to 95 ºC.

Protein sequence of OphMAΔC27-Cfa^N-SUMO.

GTSTQTKAGSLTIVGTGIESIGQMTLQALSYIEAAAKVFYCVIDPATEAFILTKNKNCVDLYQYYDNGKSRLNTYTQMSELMVREVRKGLDVVGV
FYGHPGVFVNPSHRALAIAKSEGYRARMLPGVSAEDCLFADLCIDPSNPGCLTYEASDFLIRDRPVSIHSHLVLFQVGCVGIADFNFTGFDNNKF
GVLVDRLEQEYGAEHPVVHYIAAMMPHQDPVTDKYTVAQLREPEIAKRVGGVSTFYIPPKARKASNLDIIRRLELLPAGQVPDKKARIYPANQWE
PDVPEVEPYRPSDQAAIAQLADHAPPEQYQPLATSKAMSDVMTKLALDPKALADYKADHRAFAQSVPDLTPQERAALELGDSWAIRCAMKNM
PSSLLDAARESGCLSYDTEILTVEYGFLPIGKIVEERIECTVYTVDKNGFVYTQPIAQWHNRGEQEVFEYCLEDGSIIRATKDHKFMTTDGQMLPI
DEIFERGLDLKQVDGLPKSKMSDSEVNQEAKPEVKPEVKPETHINLKVSDGSSEIFFKIKKTTPLRRLMEAFAKRQGKEMDSLRFLYDGIRIQAD
QTPEDLDMEDNDIIEAHREQAALEHHHHHH

Protein sequence of fused fOphMA2ΔC27-CfaN-SUMO.

MEHHHHHHHHTSTQTKAGSLTIVGTGIESIGQMTLQALSYIEAAAKVFYCVIDPATEAFILTKNKNCVDLYQYYDNGKSRLNTYTQMSELMVREV
RKGLDVVGVFYGHPGVFVNPSHRALAIAKSEGYRARMLPGVSAEDCLFADLCIDPSNPGCLTYEASDFLIRDRPVSIHSHLVLFQVGCVGIADFN
FTGFDNNKFGVLVDRLEQEYGAEHPVVHYIAAMMPHQDPVTDKYTVAQLREPEIAKRVGGVSTFYIPPKARKASNLDIIRRLELLPAGQVPDKKA
RIYPANQWEPDVPEVEPYRPSDQAAIAQLADHAPPEQYQPLATSKAMSDVMTKLALDPKALADYKADHRAFAQSVPDLTPQERAALELGDSW

AIRCAMKNA**SGGGGSGGGGSGGGGSGGGGSGGGGSGGGG**LQETSTQTKAGSLTIVGTGIESIGQMTLQALSYIEAAAKVFYCVIDPATEAFIL
TKNKNCVDLYQYYDNGKSRLNTYTQMSELMVREVRKGLDVVGVFYGHPGVFVNPSHRALAIAKSEGYRARMLPGVSAEDCLFADLCIDPSNP
GCLTYEASDFLIRDRPVSIHSHLVLFQVGCVGIADFNFTGFDNNKFGVLVDRLEQEYGAEHPVVHYIAAMMPHQDPVTDKYTVAQLREPEIAKR
VGGVSTFYIPPKARKASNLDIIRRLELLPAGQVPDKKARIYPANQWEPDVPEVEPYRPSDQAAIAQLADHAPPEQYQPLATSKAMSDVMTKLAL
DPKALADYKADHRAFAQSVPDLTPQERAALELGDSWAIRCAMKNMPSSLLDAARESGCLSYDTEILTVEYGFLPIGKIVEERIECTVYTVDKNGF
VYTQPIAQWHNRGEQEVFEYCLEDGSIIRATKDHKFMTTDGQMLPIDEIFERGLDLKQVDGLPKSKMSDSEVNQEAKPEVKPEVKPETHINLKV
SDGSSEIFFKIKKTTPLRRLMEAFAKRQGKEMDSLRFLYDGIRIQADQTPEDLDMEDNDIIEAHREQAALEHHHHHH

Intein-mediated peptide ligation

Sequence of Cfa^C-AA is NH_2-VKIISRKSLGTQNVYDIGVEKDHNFLLKNGLVASAAFN-NH_2. Synthetic Cfa^C-AA is dissolved in water at a concentration of 10 mM. Synthetic substrate peptides were dissolved in 50 mM Tris (pH 8.0), 100 mM NaCl, and 1 mM TCEP. For a typical ligation reaction, enzymes at a concentration of 300 μM (OphMAΔC27-CfaN-SUMO or fOphMA2ΔC27-CfaN-SUMO) in buffer (50 mM Tris, pH 8.5 and 100 mM NaCl) were added with 1 mM TCEP, 1 mM EDTA, 100 mM MESNA, 1.5 mM synthetic substrate peptides and 1 mM Cfa^C-AA sequentially to a total volume of 50 ul. 20 mM TCEP (pH 8.0) was added to the reaction mixture after 20 min and the ligation reaction was incubated at 20 ºC. The splicing was checked and visualized by SDS-PAGE at selected 4 h and 20 h time points. All the ligation reactions for peptides containing non-natural amino acids were incubated for 20 h at 20 ºC before removal of precipitation by centrifugation. Around 450 ul of buffer (50 mM Tris pH 8.0, 100 mM NaCl, 20 mM TCEP) was added to the reaction mixture before loading onto a PD Miditrap G-25 desalting column to remove excess MESNA and EDTA. Proteins collected were analysed using TOF-MS to check the ligation status. Synthetic peptides were removed by extensive diafiltration through a 50 kDa concentrator (Vivaspin 20) and removal of the synthetic peptide (~ 2kDa) was confirmed by checking the filtrate using LC-MS. The concentrated ligated protein was aliquoted and stored in buffer (50 mM Tris pH 8.0, 10% glycerol, 100 mM NaCl, and 2 mM TCEP) at -80 ºC.

ESI-TOF MS and intact mass measurement

The intact mass for all the protein samples were recorded on a LC-MS Waters Xevo G2-QS QToF MS coupled to a ACQUITY UPLC I-Class LC system (Waters Corporation). The spectrometer was auto-calibrated throughout the experiment via an internal lock-spray mass calibrant (at 556.2771 m/z of leucine enkephalin, 1 s scan every 120 s interval). The data was acquired in Continuum format, in a 300 – 2500 m/z spectral window with a scan time of 1 s and an interscan time of 0.014 s. The mass spectrometer and ESI ionization source operated under following parameters: capillary voltage 3 kV, cone voltage 20 V, source offset voltage 40 V, source temperature 100 °C, desolvation temperature 400 °C and desolvation gas (nitrogen) flow 650 L/h, cone gas flow 30 L/h. In a typical analysis, 1 µL (30 ng) of the protein sample was injected and desalted on a ProSwift™ RP-1S HPLC (250 x 4.6 mm x 5 µm, Thermo Scientific™) column maintained at 60 ºC. The elution was performed using solvent A (water with 0.1% formic acid) and solvent B (acetonitrile with 0.1% formic acid) with a gradient elution using isocratic 95% solvent A (0 - 1 min), linear gradient to 95% solvent B (1.1 – 7 min), and isocratic 95% solvent A (7.1–10 min) at a total flow of 0.2 ml/min. Combined mass spectra from the total chromatographic protein peak were used for intact mass reconstruction using MassLynx (Waters) Max(imum) Ent(ropy) 1 deconvolution algorithm (Resolution: 1.00 Da/channel, Width at half height: ion series/protein dependent, Minimum intensity ratios: 33% Left and Right). Spectra were deconvoluted between 80000 and 95000 for fOphMA2 ligated with peptide, between 40000 and 50000 for OphMA ligated with peptide, OphMAΔC12, and OphMAΔC12-390Cys, between 100000 and 120000 for fOphMA2ΔC27-CfaN-SUMO, between 60000 and 80000 for OphMAΔC27-CfaN-SUMO. The ratio of unligated spliced protein to ligated protein were calculated from relative peak intensities in the deconvoluted spectra. An oxidation product consistently occurred during ligation and purification of OphMA but not for fused dimer fOphMA2.

***In vitro* assay**

To assess the *in vitro* methylation activity of fused OphMA dimer, the ligated product was incubated with 10 mM SAM for three days in buffer (50 mM Tris pH 8.0, 100 mM NaCl, 10% glycerol and 2 mM TCEP) at 20 ºC. The samples were frozen at – 80 ºC for mass analysis.

nLC-ESI-MS/MS analysis

All the nanoliquid chromatography (nLC)–MS/MS data were recorded on a Sciex 5600+ mass spectrometer equipped with the Eksigent 2D Ultra NanoLC system. Typically, samples were digested at 37°C overnight using sequencing-grade Glu-C (Promega) at 1:50. After injection, the resulting peptides were held on a trap column (Thermo Scientific Pepmap C18 column, 100 µm × 2 cm) for 5-min washing with buffer A (2% CH_3CN, 98% H_2O, 0.05 % trifluoroacetic acid). The trap was later switched in-line with the analytical column (Thermo Scientific Pepmap C18 column, 75 µm × 15 cm) and elution was performed with following gradient: linear 5-40% B (buffer B, 98% CH_3CN, 2% H_2O, 0.1% formic acid) for 6 min, linear 40-95% B for 3 min, isocratic 95% B for 3 min, linear 95-5% B for 2 min, and isocratic 5% B for 6 min. Mass spectra were acquired in positive-ion mode in information-dependent acquisition mode (IDA) by performing 150 ms of MS followed by 80 ms of MS/MS analysis. These masses were then excluded from analysis for the next 10 s. MS spectra were acquired from 400 to 1350 m/z, and MS/MS spectra were acquired from 95 to 2000 m/z. MS/MS data were extracted using the SCIEX PeakView software. The MS spectra were used to create extracted ion chromatograms of the masses of interest ±0.05 m/z, and the individual peak areas were measured using the PeakView software. Better MS/MS spectra were further collected utilizing product ion mode (PIS) scanned specifically for the masses of interest of the C-terminal Glu-C digested peptide under the same LC condition as above and manually interpreted.

Results and Discussion

Figure S1. Scheme for the split intein mediated peptide ligation to OphMA.

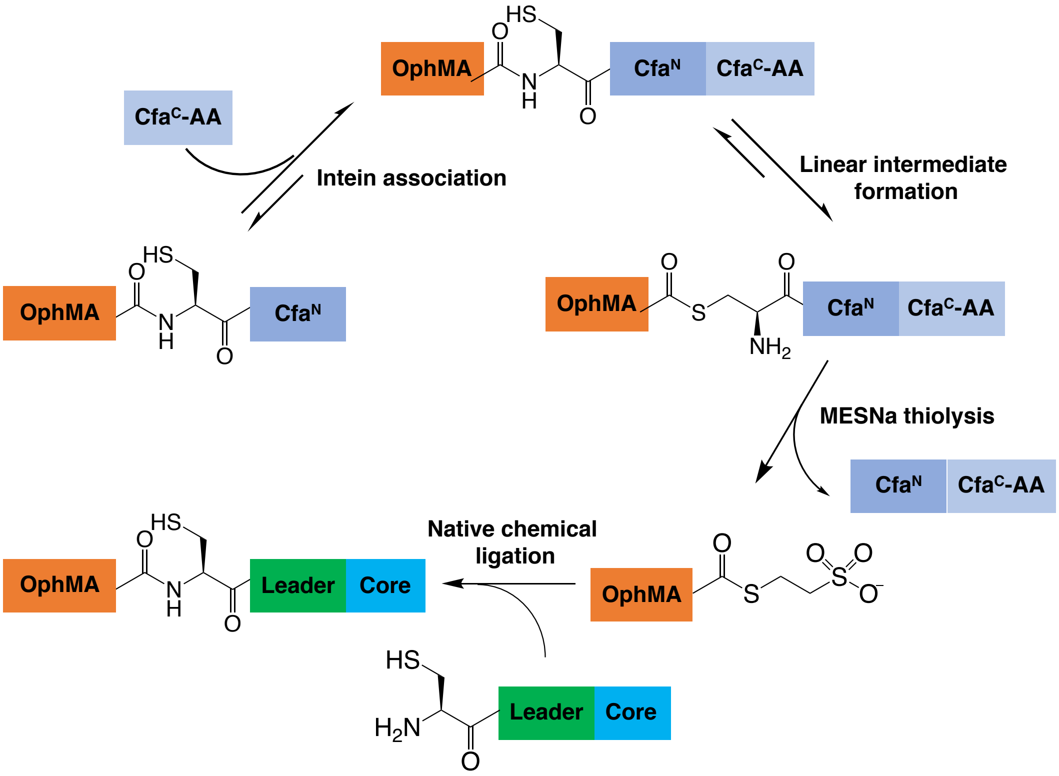

Figure S2. Gel filtration profile of OphMA∆C12-G390_E391insCys and fOphMA2∆C12-G390_E391insCys. Both proteins were loaded on a HiLoad Superdex S200 pg column equilibrated with 25 mM Tris pH 8.0, 100 mM NaCl, 10% glycerol and 2 mM TCEP. The retention volume for both proteins suggested a catalytic dimer (catanene dimer).

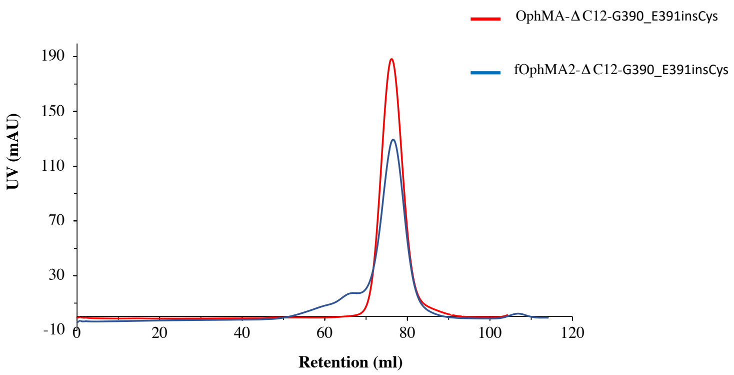

Figure S3. Thermal shift assay for OphMA∆C12-G390_E391insCys and fOphMA2∆C12-G390_E391insCys. The assays were performed at a concentration of 0.5 mg/ml in 50 mM Tris pH 8.0, 100 mM NaCl, 10% glycerol and 2 mM TCEP. Both proteins have same amount of catanene dimer at the same protein concentration. The experiments were repeated in triplicates. OphMA-∆C12-G390_E391insCys has an aggregation onset temperature (T_{agg}) of 39.1 °C while fOphMA2-∆C12-G390_E391insCys has a higher T_{agg} (43.5 °C), suggesting that fused catanene dimer is more stable than unfused dimer.

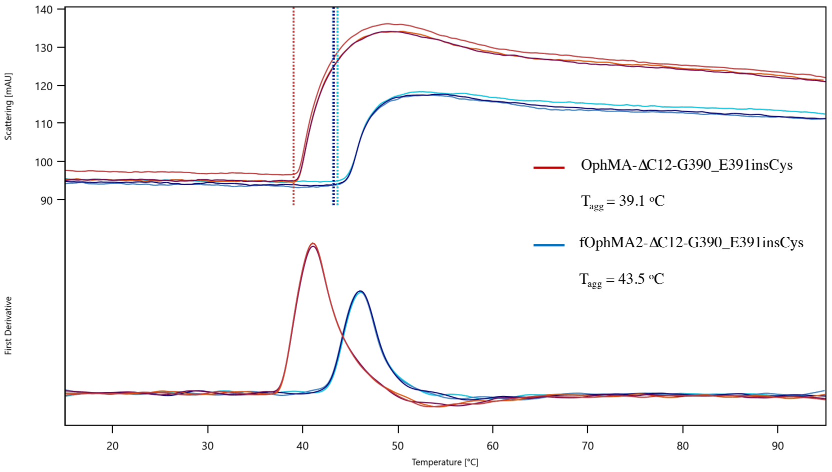

Figure S4. (a) HPLC (0-90% gradient of MeCN in H_2O with 0.1% TFA for the purified Cfa^{C}-AA peptide. (b) ESI-MS for the purified Cfa^{C}-AA peptide. (c) SDS-Page gel analysis of splicing reaction within valine peptide (performed in triplicates and monitored at 4 h and 20 h). Relative quantitation suggests similar splicing rate. The triplicates were subjected to QToF mass for ligation efficiency.

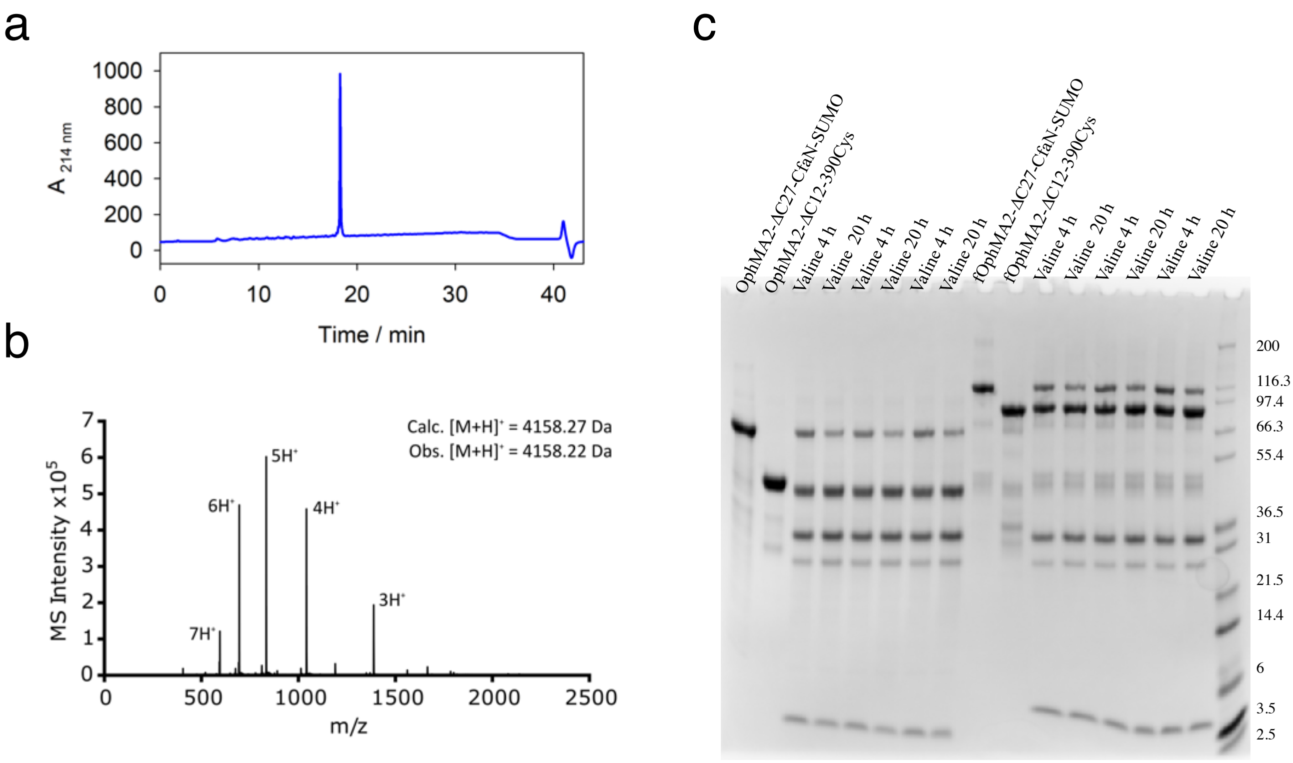

Figure S5. SDS-Page gel shows splicing using fOphMA2ΔC12-G390_E391insCys, Cfa^{C}-AA, and peptides containing non-proteinogenic amino acid at position 401. The reactions were performed for 20 h.

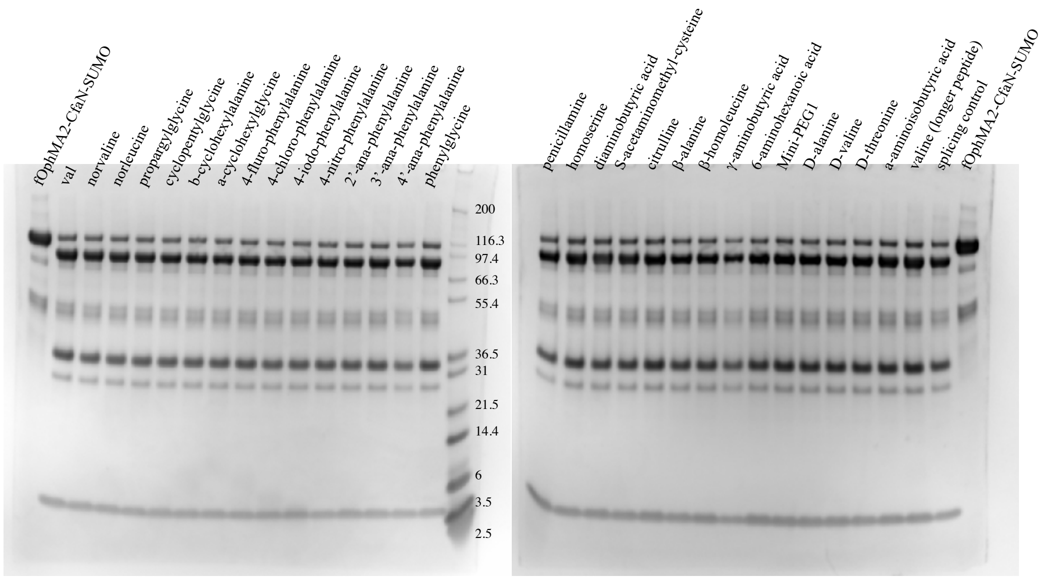

Figure S6. Intact mass of proteins. Proteins at a concentration of 0.02-0.03 mg/ml in 0.1% formic acid and 2 mM TCEP were loaded to Xevo G2-QToF Mass for intact mass analysis.

aa

OphMA-ΔC12

Expected: 44413 Da, **Observed**: 44410 Da (0 Met), 44425 (1 Met), 44439 (2 Met)

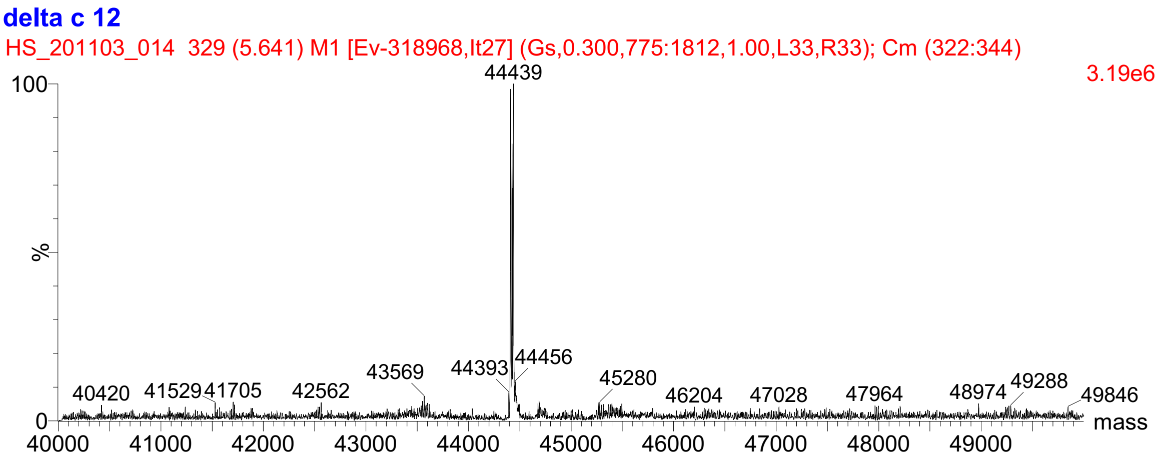

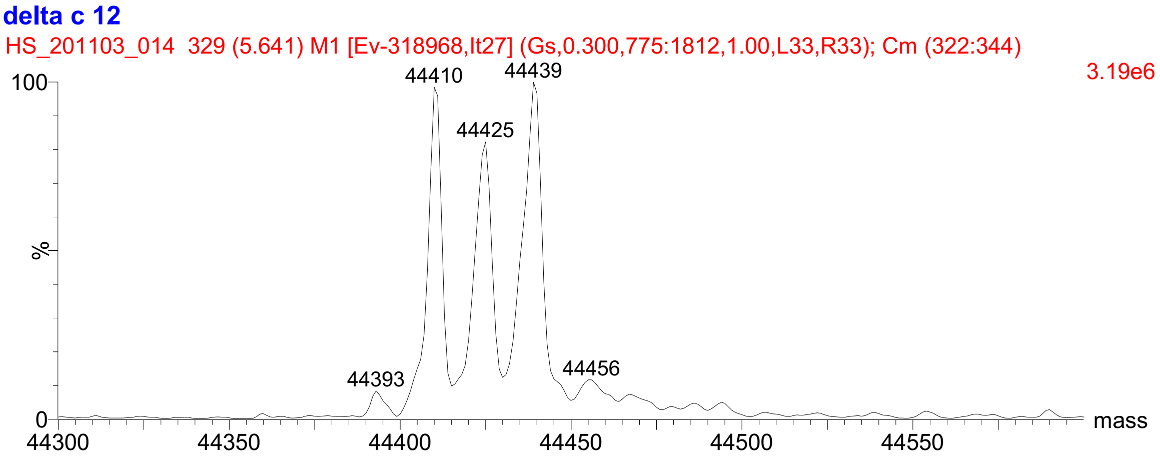

ab

OphMA-ΔC12-G390_E391insCys

Expected: 44517 Da, **Observed**: 44511 Da (0 Met), 44527 (1 Met), 44543 (2 Met)

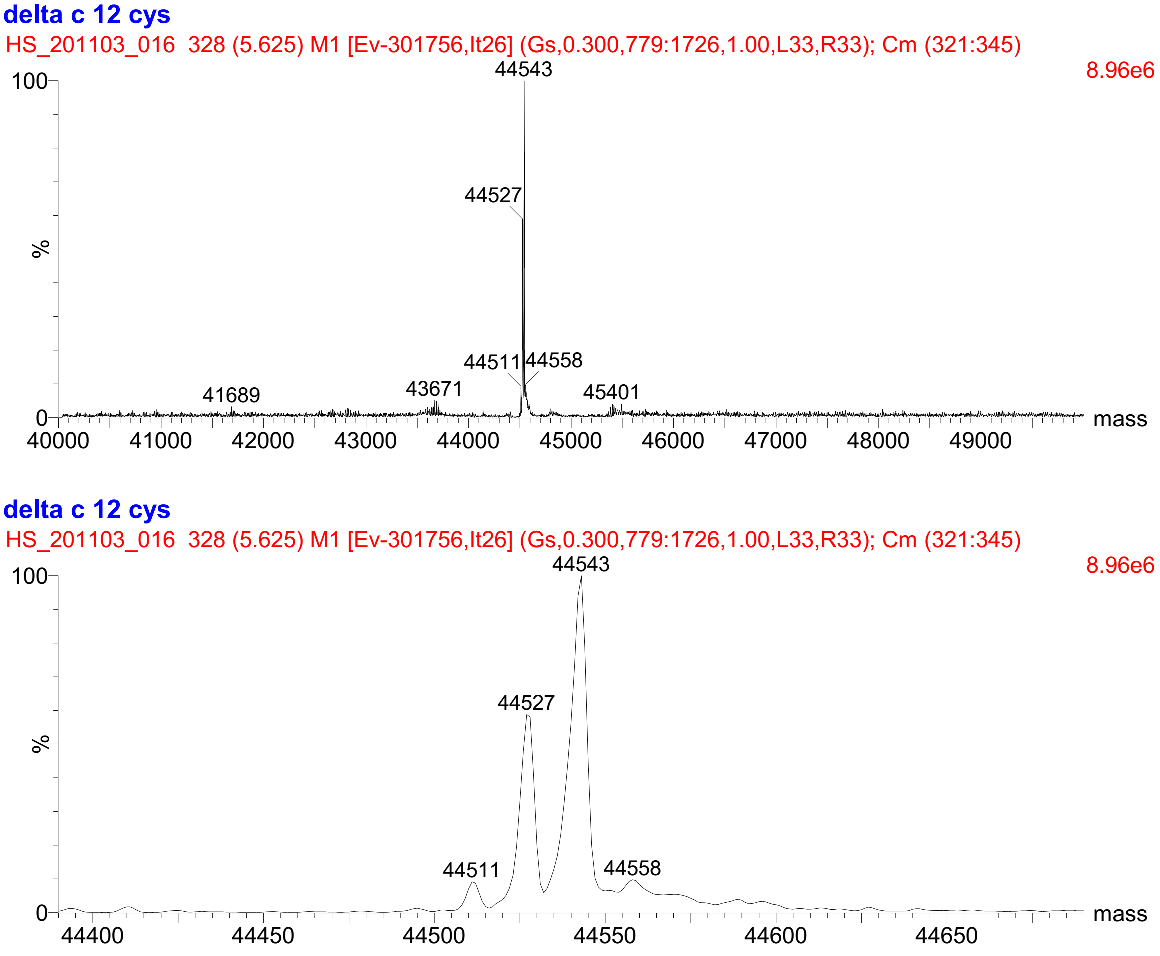

ac

OphMA-ΔC27-CfaN-SUMO

Expected: 67013 Da, **Observed**: 67012 Da

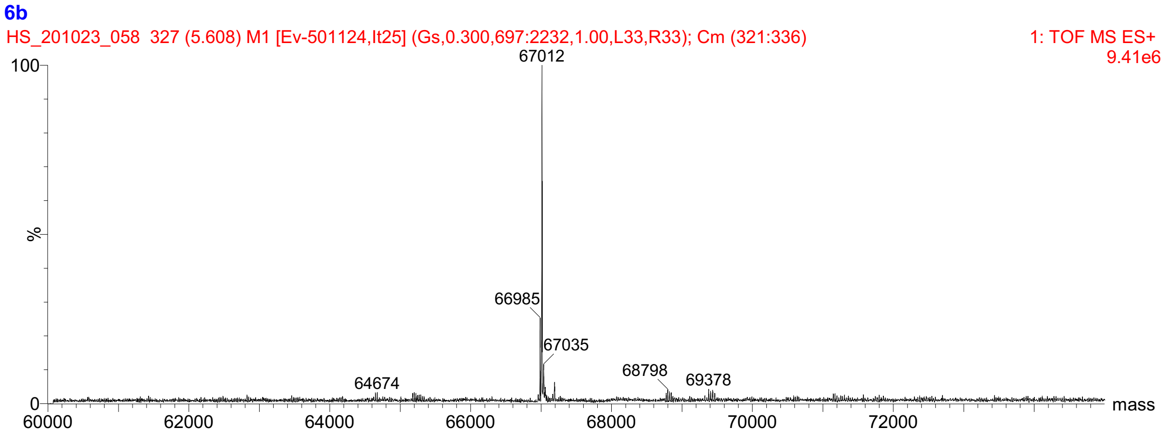

ad

fOphMA2-ΔC27-CfaN-SUMO

Expected: 67013 Da, **Observed**: 67012 Da

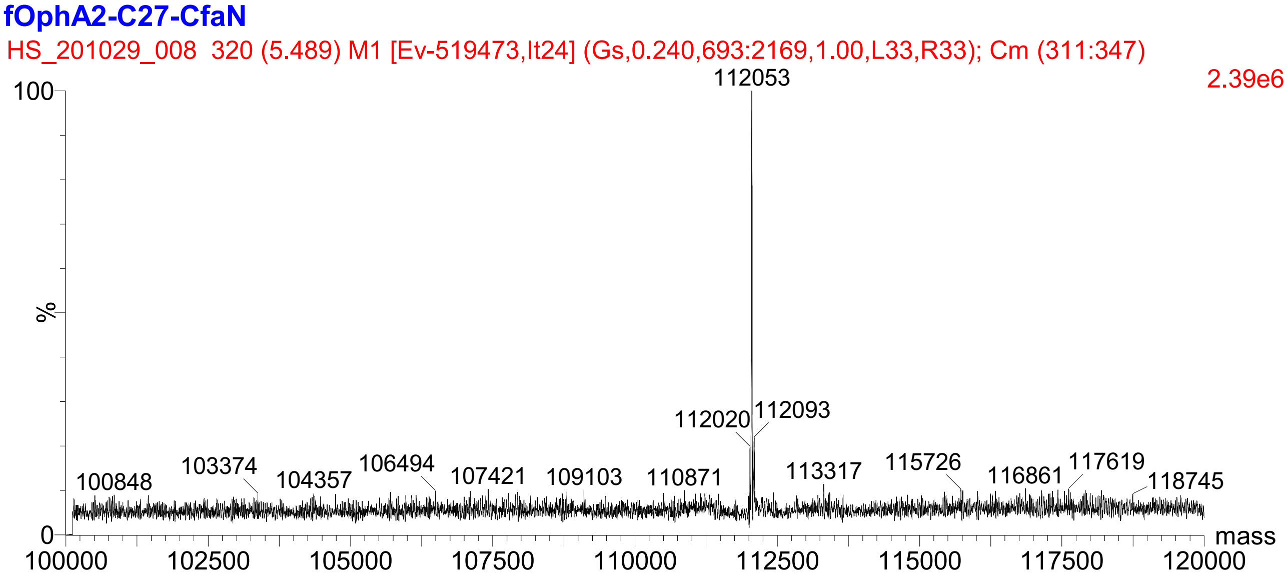

ae

Splitting control in the absence of substrate peptide

Calculated mass: 87984 Da, Observed mass: 87942 Da

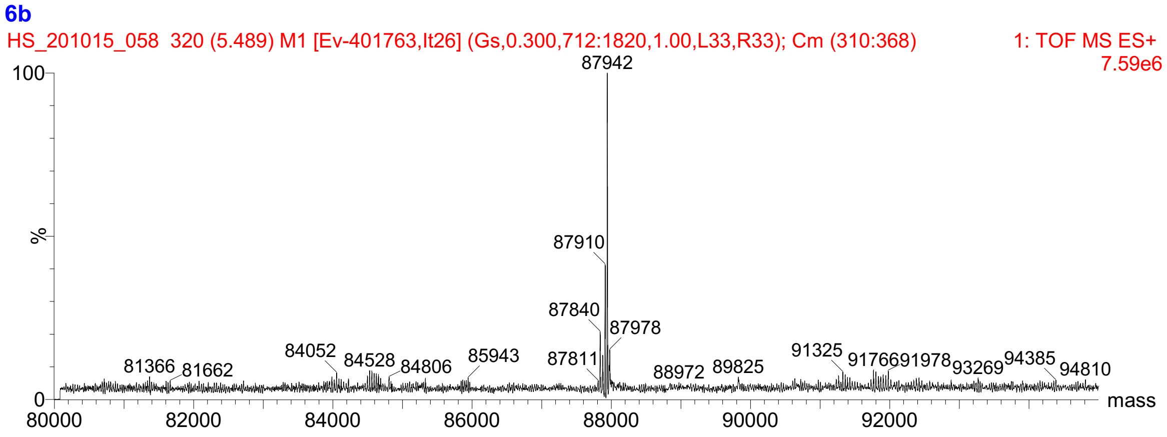

af

OphMA-ΔC12-401Val

Expected: 44517, Observed, 44514 Da, 44528 Da (1 Oxidation), 44543 (2 Oxidation).

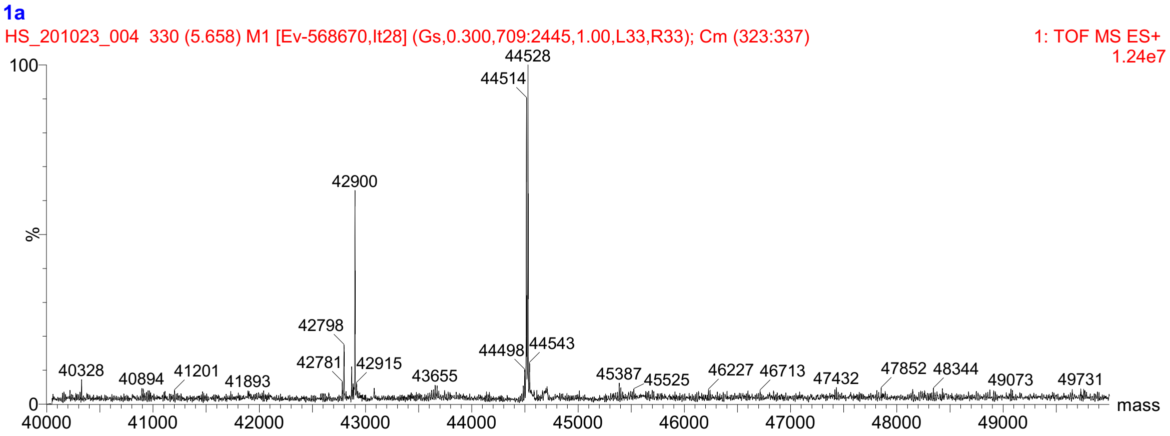

ag

fOphMA2-ΔC12-401Val:

Calculated mass: 89559 Da, Observed mass: 89557 Da

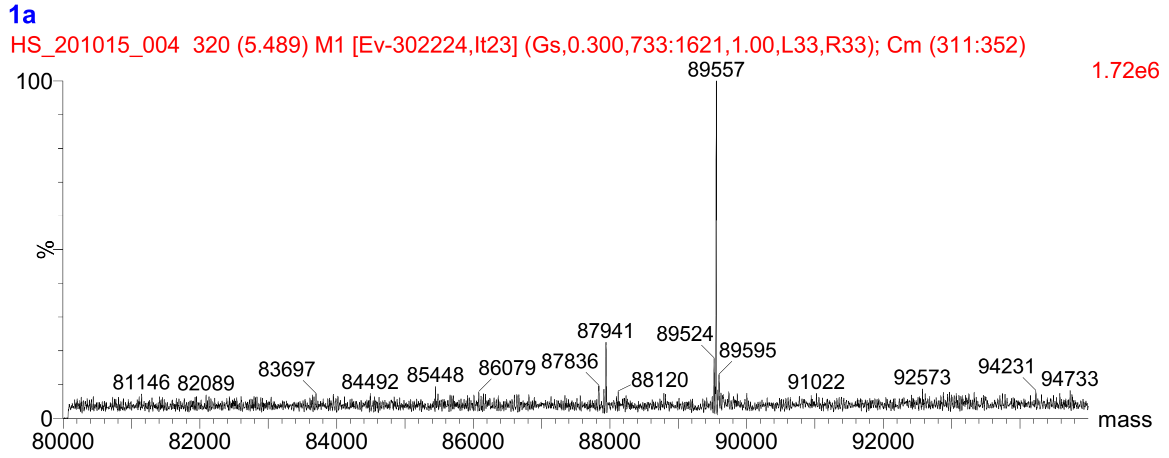

ah

fOphMA2-ΔC12-401Nva

Calculated mass: 89559 Da, Observed mass: 89556 Da

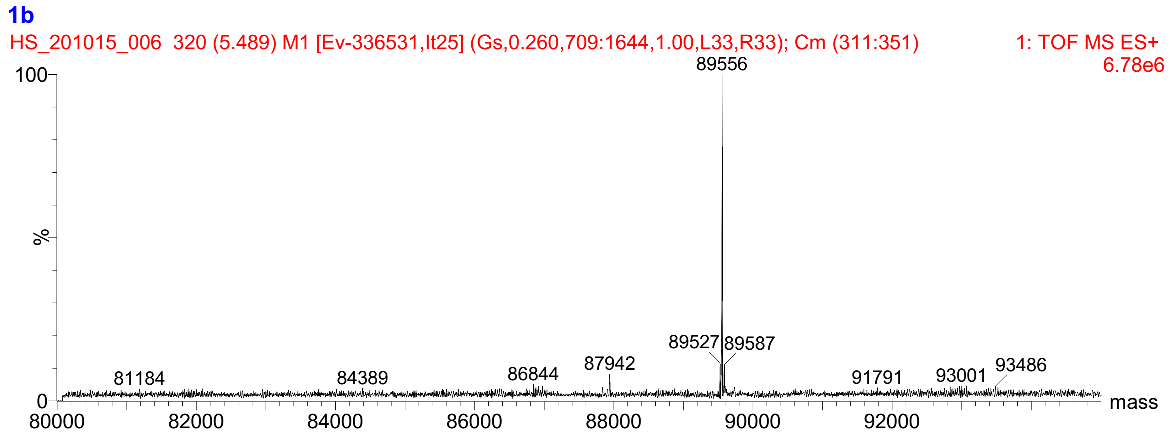

ai

fOphMA2-ΔC12-401Nle

Calculated mass: 89573 Da, Observed mass: 89571 Da

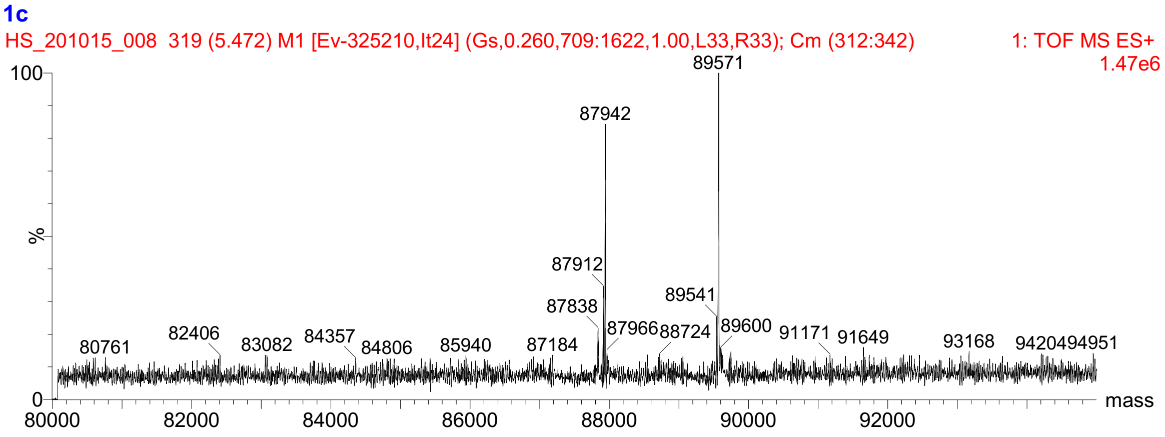

aj

fOphMA2-ΔC12-401Pra

Calculated mass: 89555 Da, Observed mass: 89552 Da

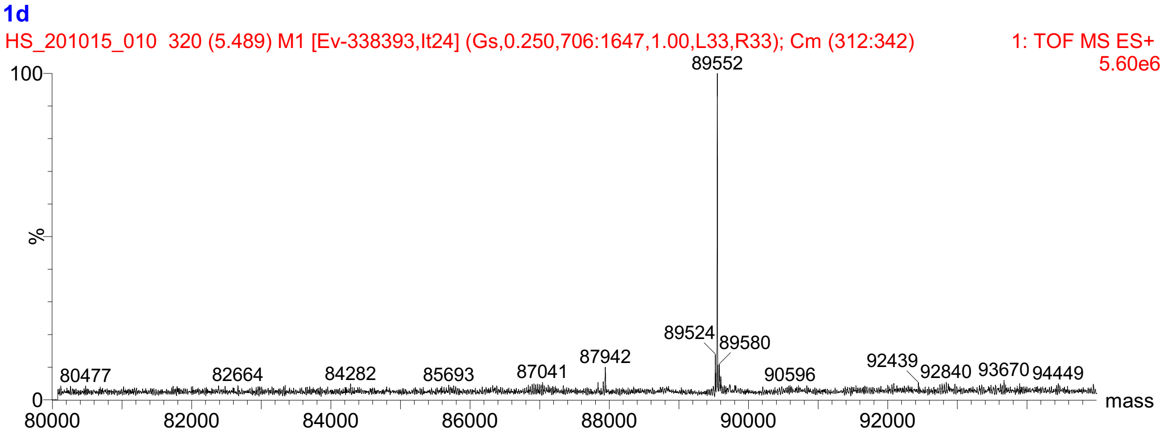

ak

fOphMA2-ΔC12-401Cha

Calculated mass: 89613 Da, Observed mass: 89610 Da

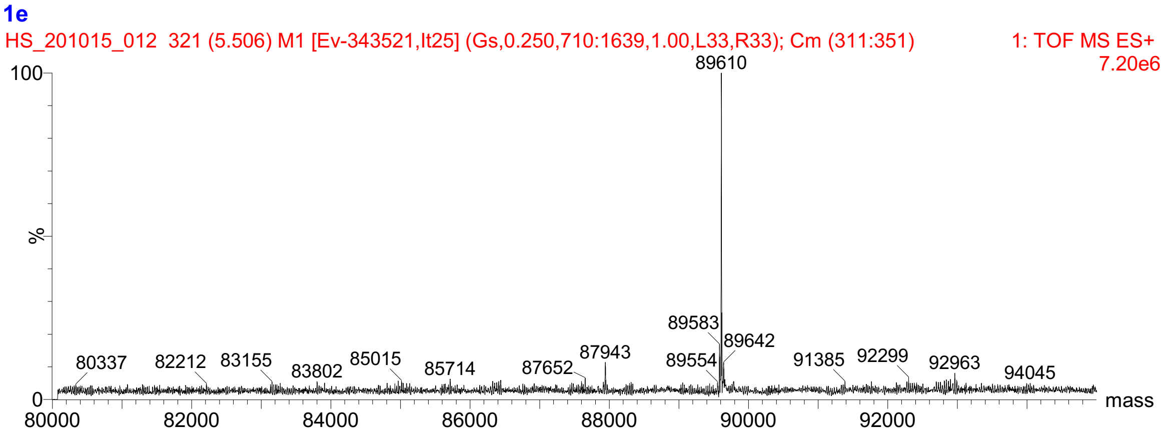

al

fOphMA2-ΔC12-401Chg

Calculated mass: 89599 Da, Observed mass: 89596 Da

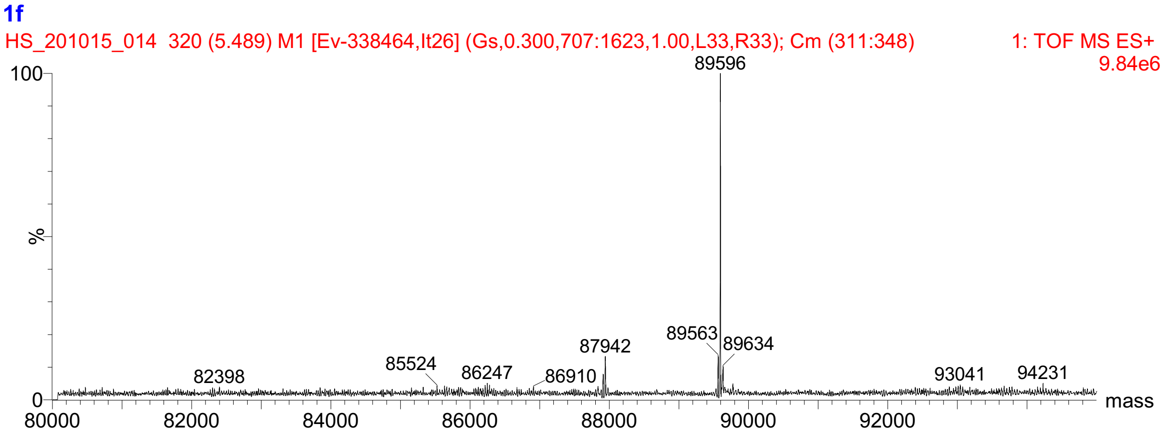

am
fOphMA2-ΔC12-401Cpg

Calculated mass: 89599 Da, Observed mass: 89598 Da

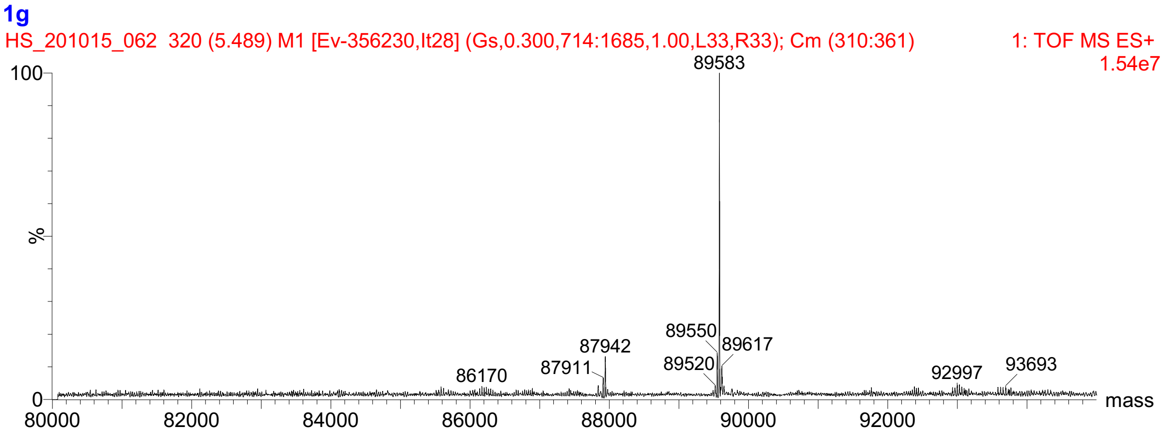

an
fOphMA2-4014-F-Phe

Calculated mass: 89625 Da, Observed mass: 89622 Da

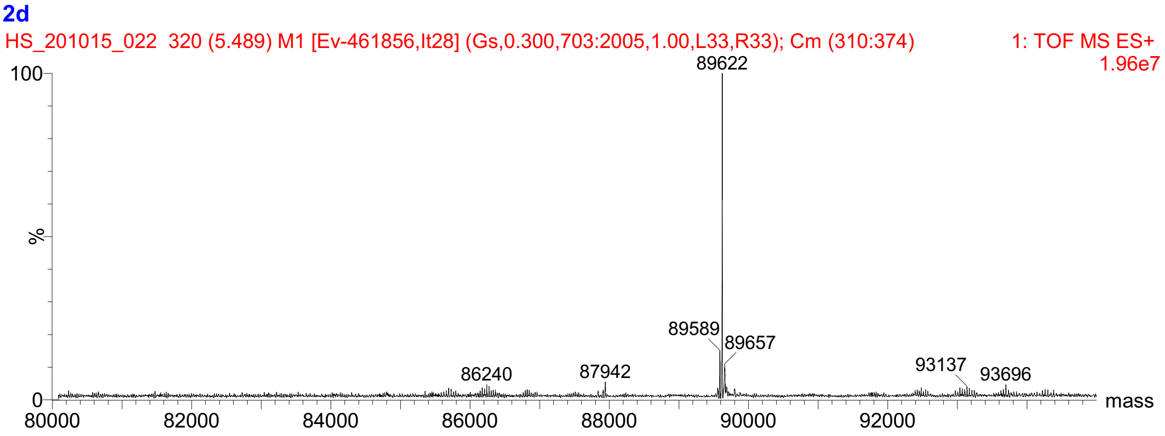

ao

fOphMA2-ΔC12-401-4-Cl-Phe

Calculated mass: 89641 Da, Observed mass: 89638 Da

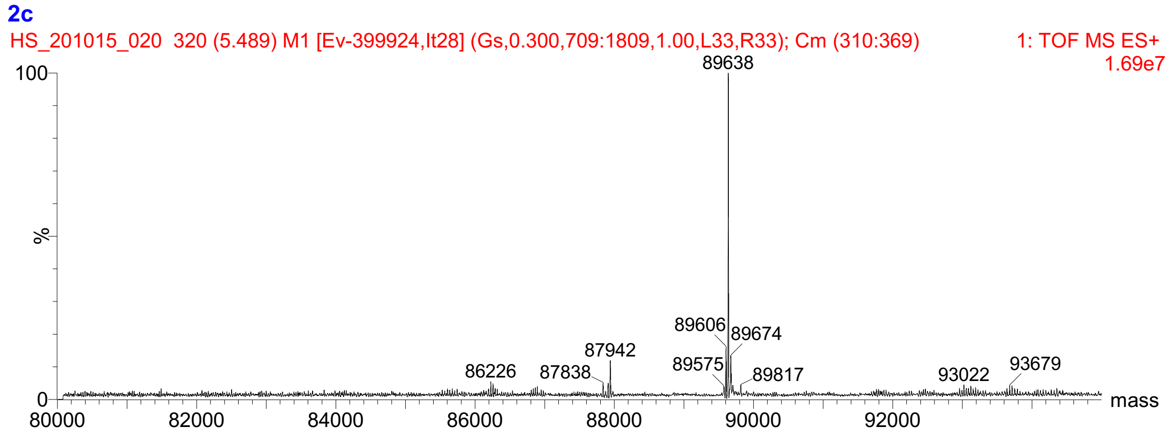

ap

fOphMA2-ΔC12-401-4-I-Phe

Calculated mass: 89733 Da, Observed mass: 89730 Da

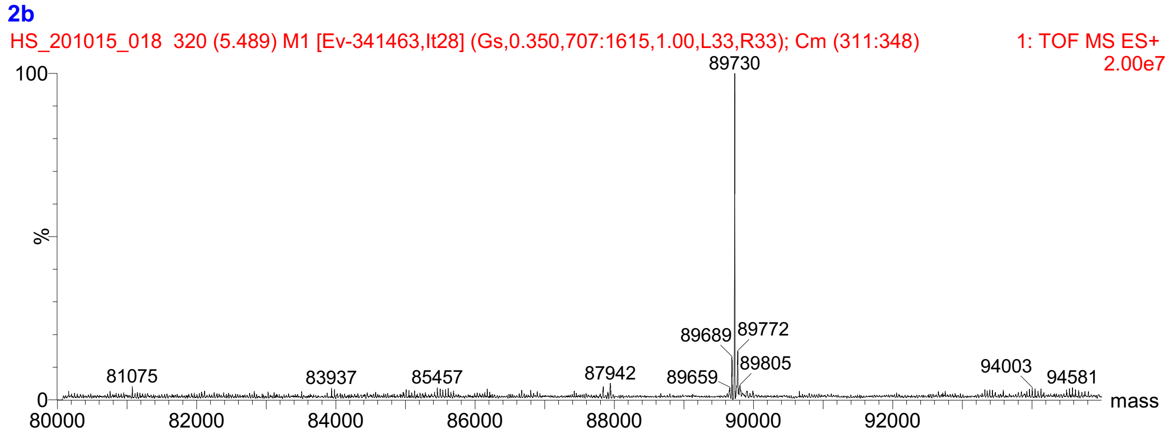

aq

fOphMA2-ΔC12-401-4-NO2-Phe

Calculated mass: 89652 Da, Observed mass: 89649 Da

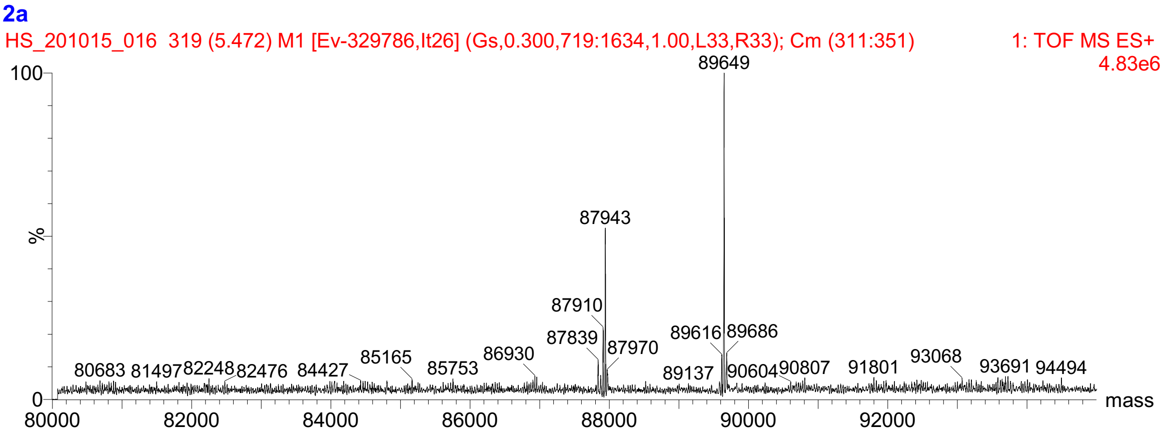

ar

fOphMA2-ΔC12-401-2-Pal

Calculated mass: 89608 Da, Observed mass: 89605 Da

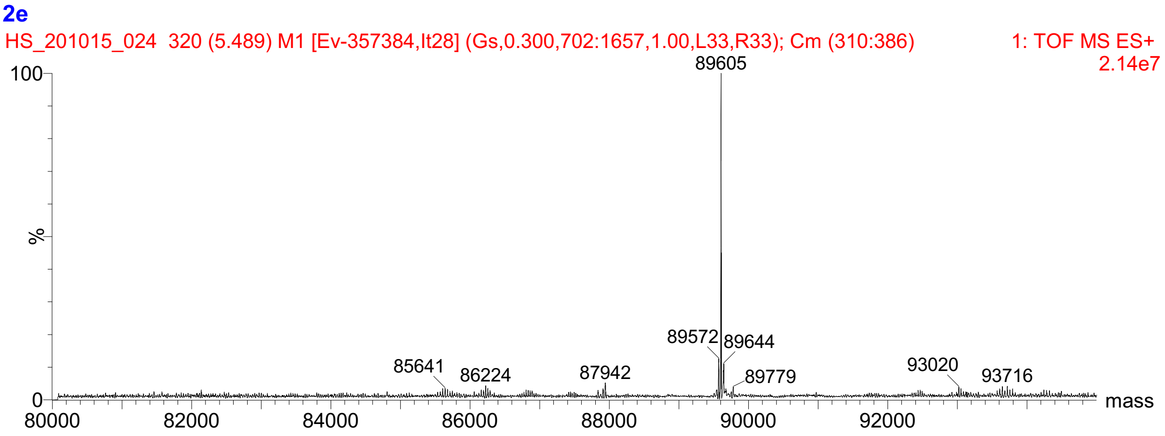

ar

fOphMA2-ΔC12-401-3-Pal

Calculated mass: 89608 Da, Observed mass: 89605 Da

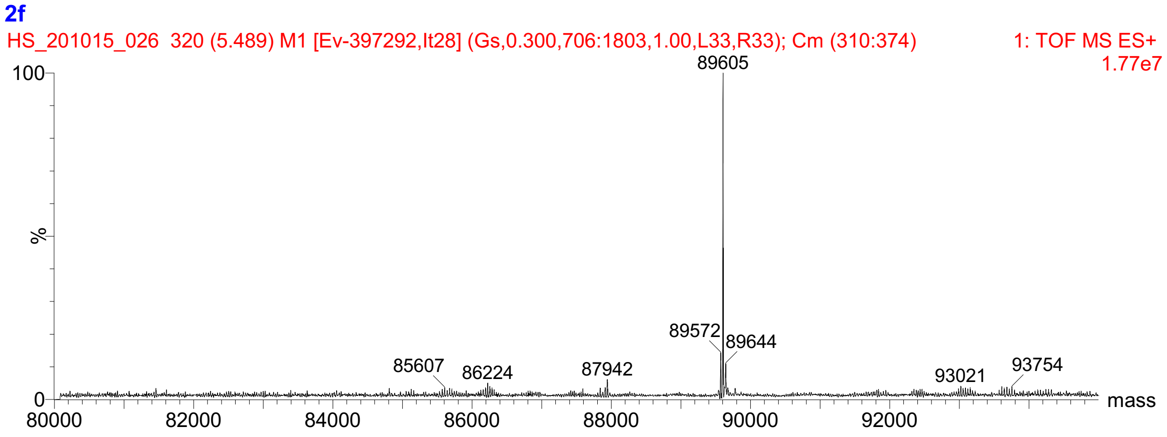

at

fOphMA2-ΔC12-401-4-Pal

Calculated mass: 89608 Da, Observed mass: 89605 Da

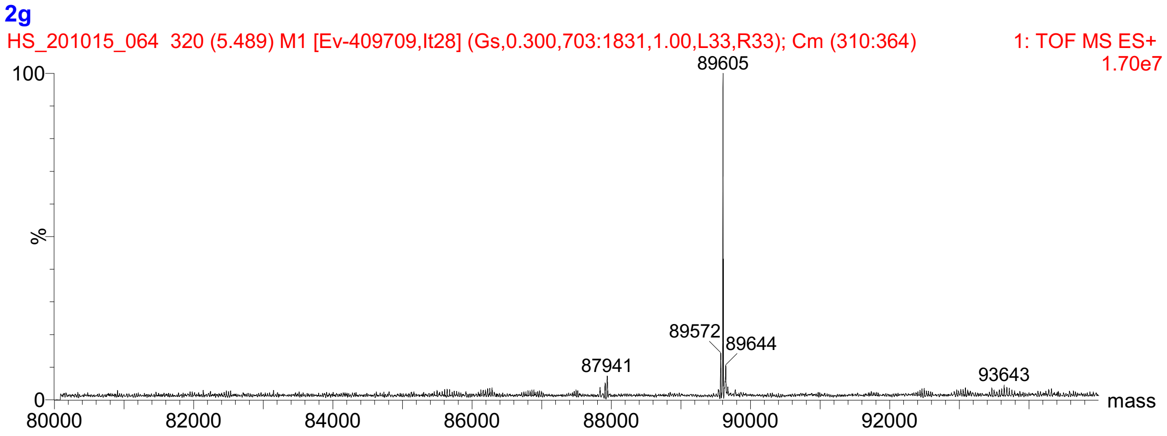

au

fOphMA2-ΔC12-401Phg

Calculated mass: 89593 Da, Observed mass: 89590 Da

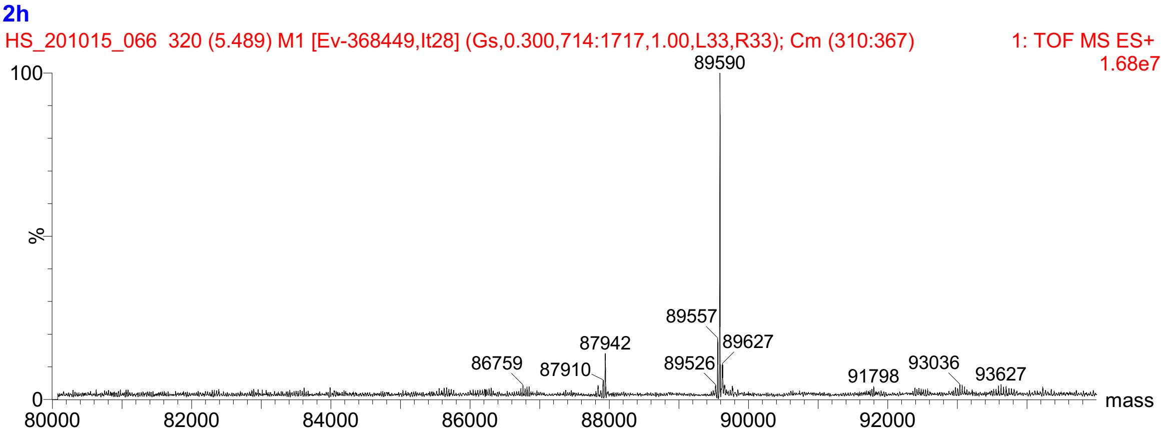

av

fOphMA2-ΔC12-401Pen

Calculated mass: 89591 Da, Observed mass: 89588 Da

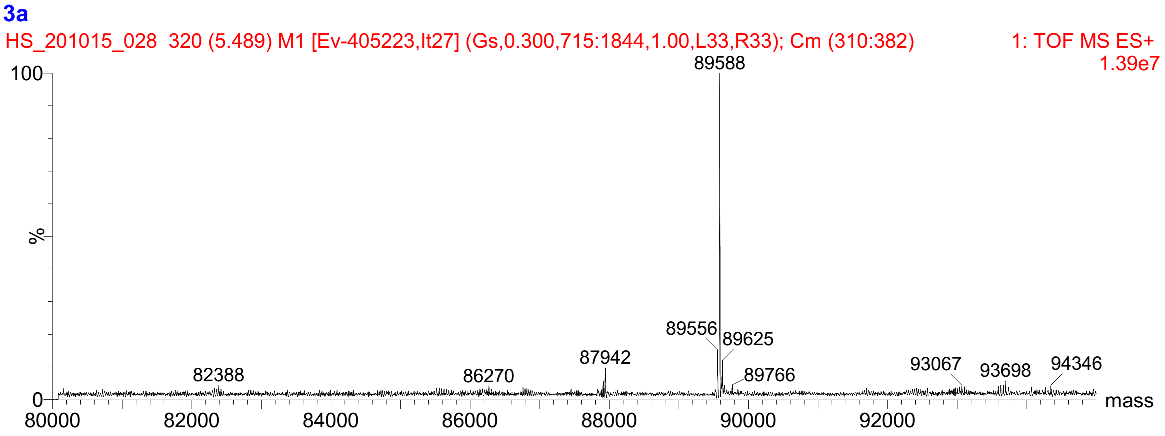

aw

fOphMA2-ΔC12-401Hse

Calculated mass: 89561 Da, Observed mass: 89559 Da

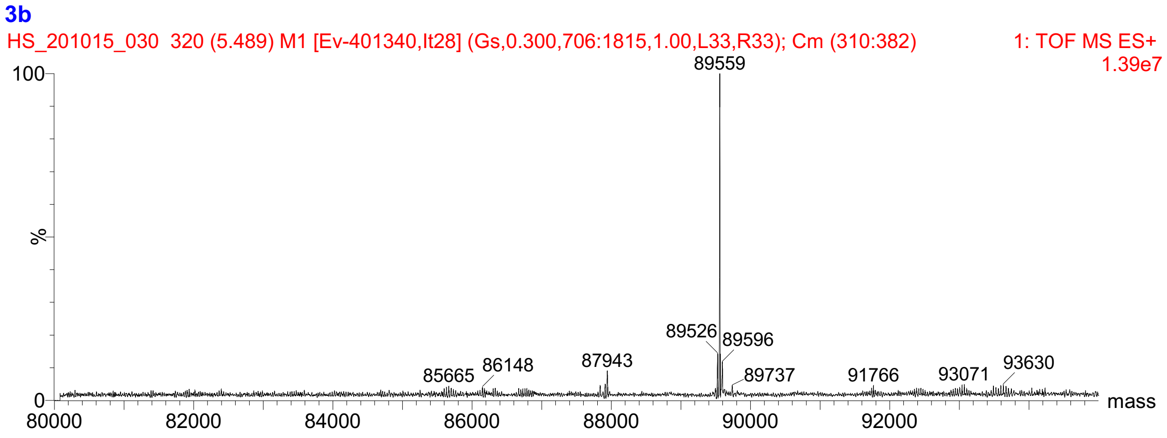

ax

fOphMA2-ΔC12-401Dab

Calculated mass: 89560 Da, Observed mass: 89557 Da

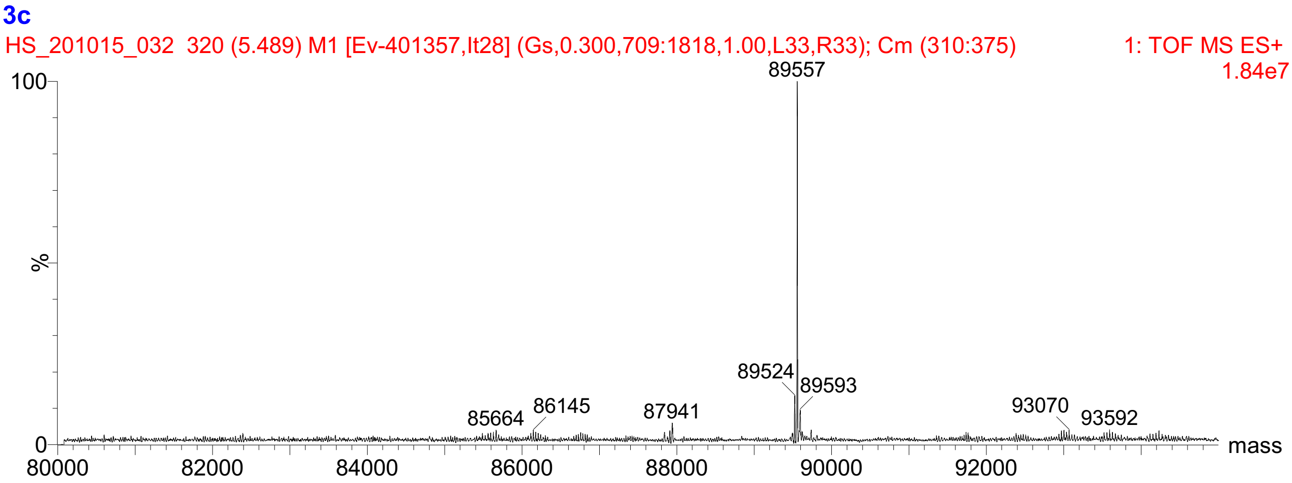

ay

fOphMA2-ΔC12-401CysACM

Calculated mass: 89634 Da, Observed mass: 89631 Da

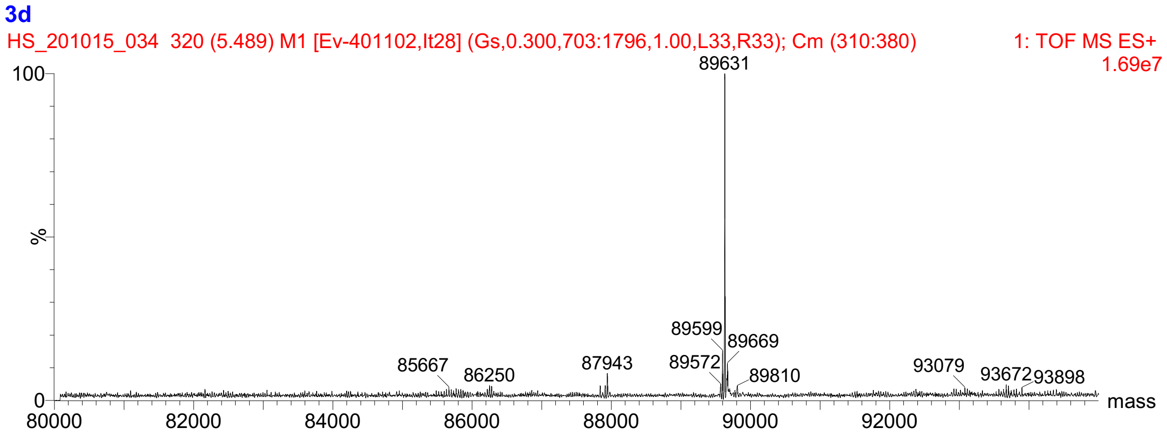

az

fOphMA2-ΔC12-401CIT

Calculated mass: 89617 Da, Observed mass: 89614 Da

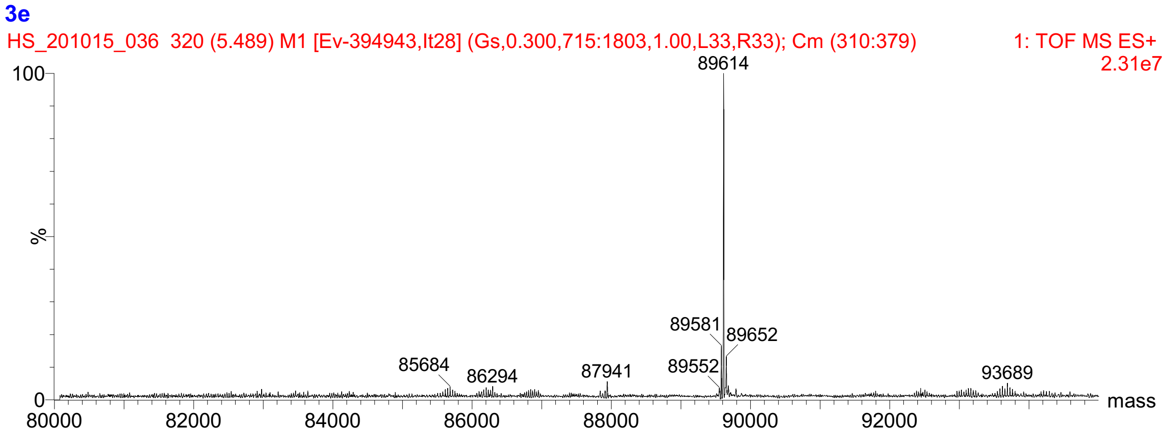

ba

fOphMA2-ΔC12-401Gaba

Calculated mass: 89545 Da, Observed mass: 89542 Da

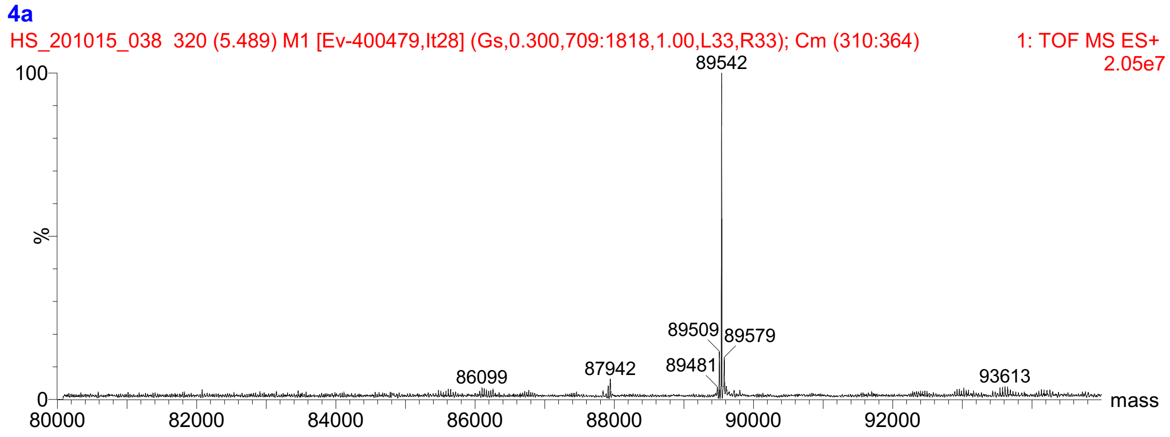

bb

fOphMA2-ΔC12-401-β-Ala

Calculated mass: 89531 Da, Observed mass: 89528 Da

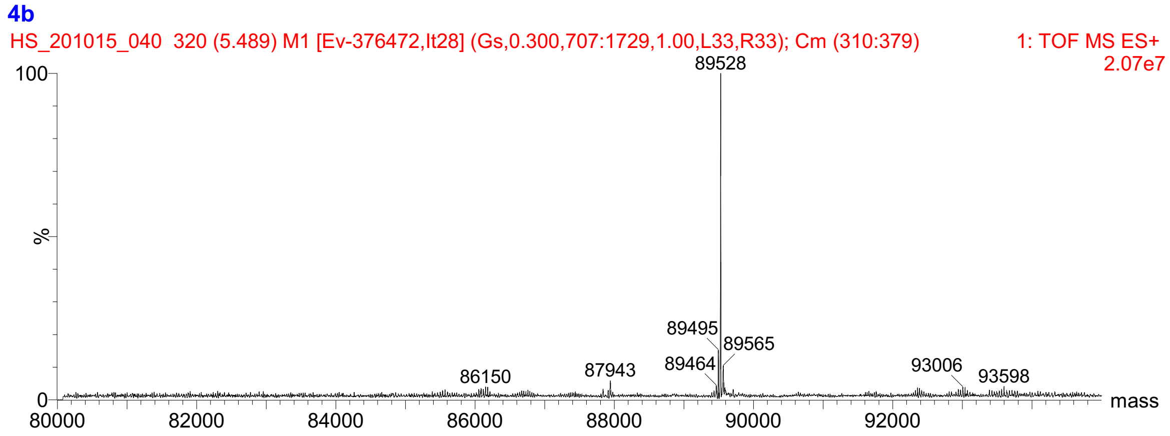

bc

fOphMA2-ΔC12-401-β-homoleucine

Calculated mass: 89587 Da, Observed mass: 89584 Da

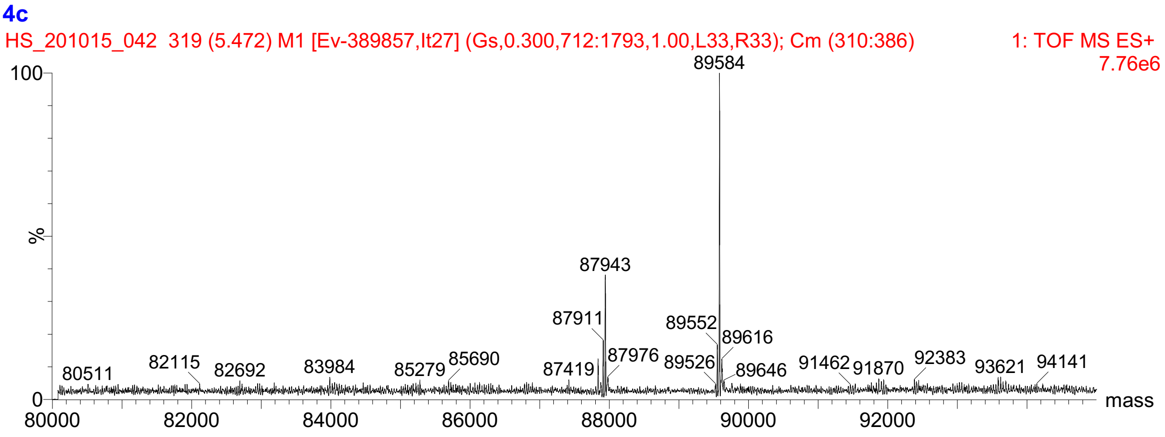

bd

fOphMA2-ΔC12-401Ahx

Calculated mass: 89573 Da, Observed mass: 89570 Da

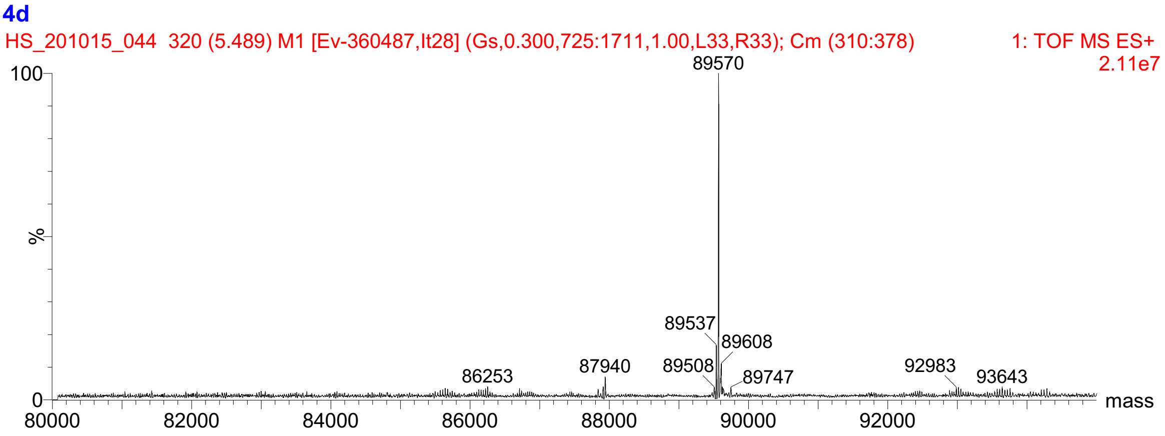

be

fOphMA2-ΔC12-401-D-Ala

Calculated mass: 89531 Da, Observed mass: 89528 Da

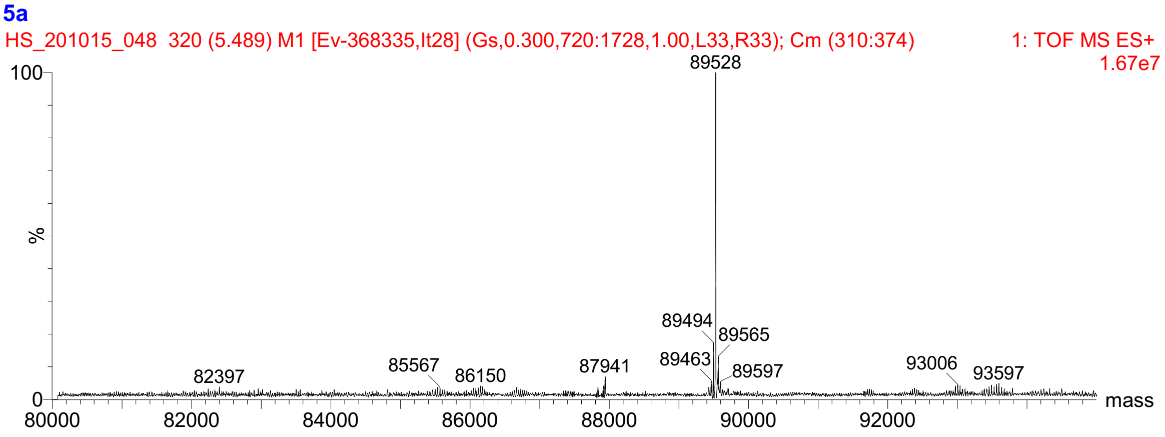

bf

fOphMA2-ΔC12-401-D-Val

Calculated mass: 89559 Da, Observed mass: 89556 Da

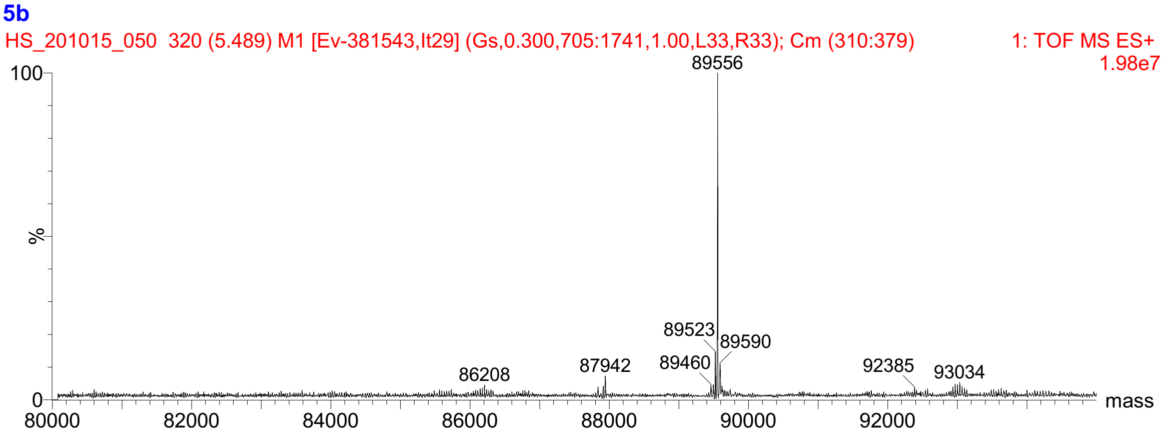

bg

fOphMA2-ΔC12-401-D-Thr

Calculated mass: 89561 Da, Observed mass: 89558 Da

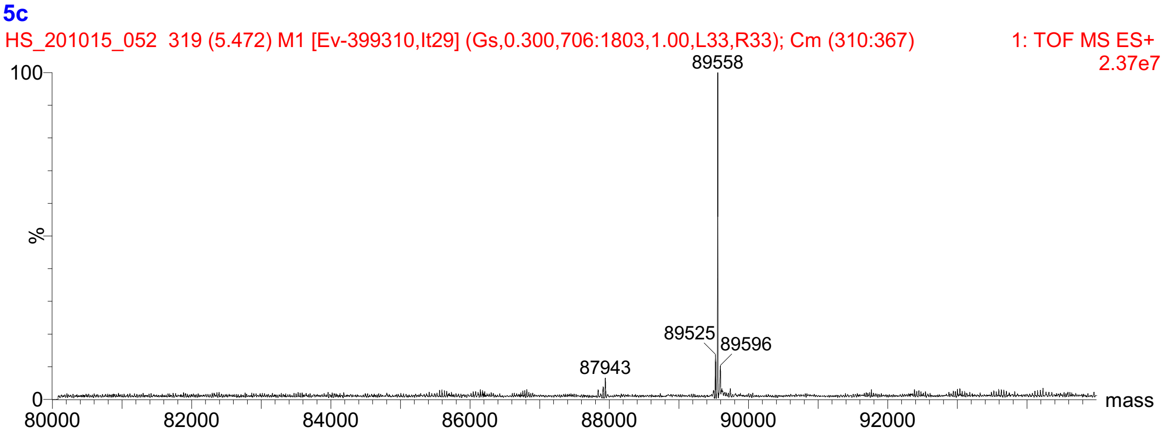

bh

fOphMA2-ΔC12-401Aib

Calculated mass: 89545 Da, Observed mass: 89542 Da

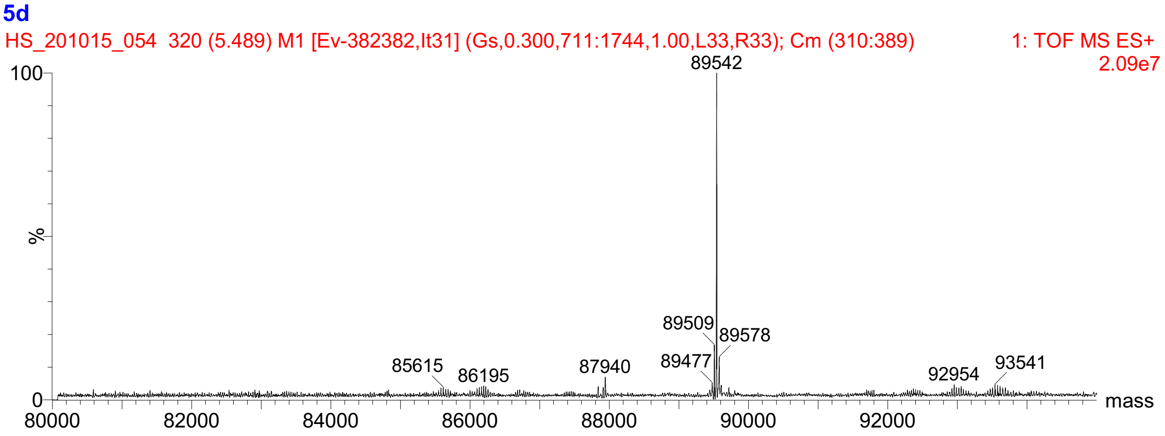

bi

fOphMA2-Peptide (CEEASQNGFPWVIVVGVIG)

Calculated mass: 89829 Da, Observed mass: 87943 Da

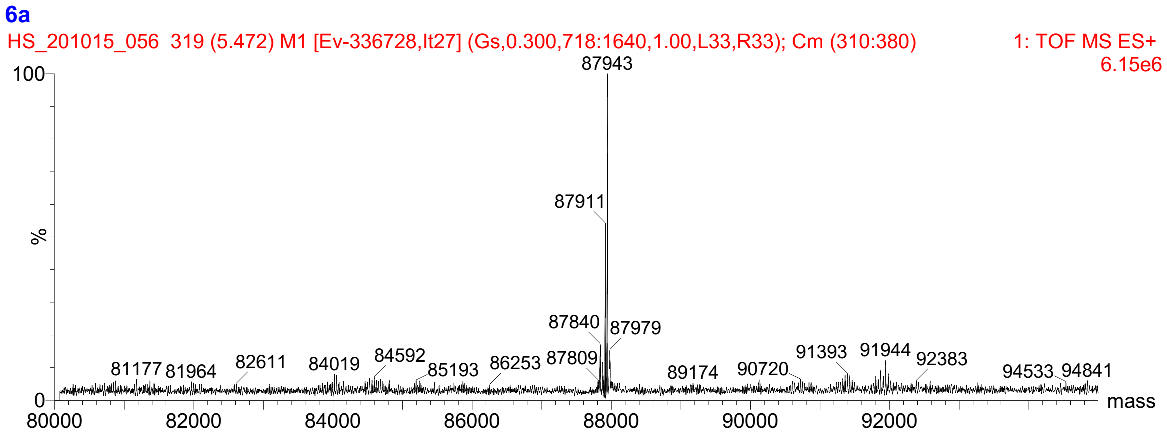

Figure S7. MS analysis of Glu-C digested various ligated fOphMA2 after three days' incubation with SAM. Peptides with hydrophobic side chains (**A**), aromatic side chains (**B**), hydrophilic or charged side chains (**C**), non-alpha amino acids (**D**) and D-amino acids (**E**).

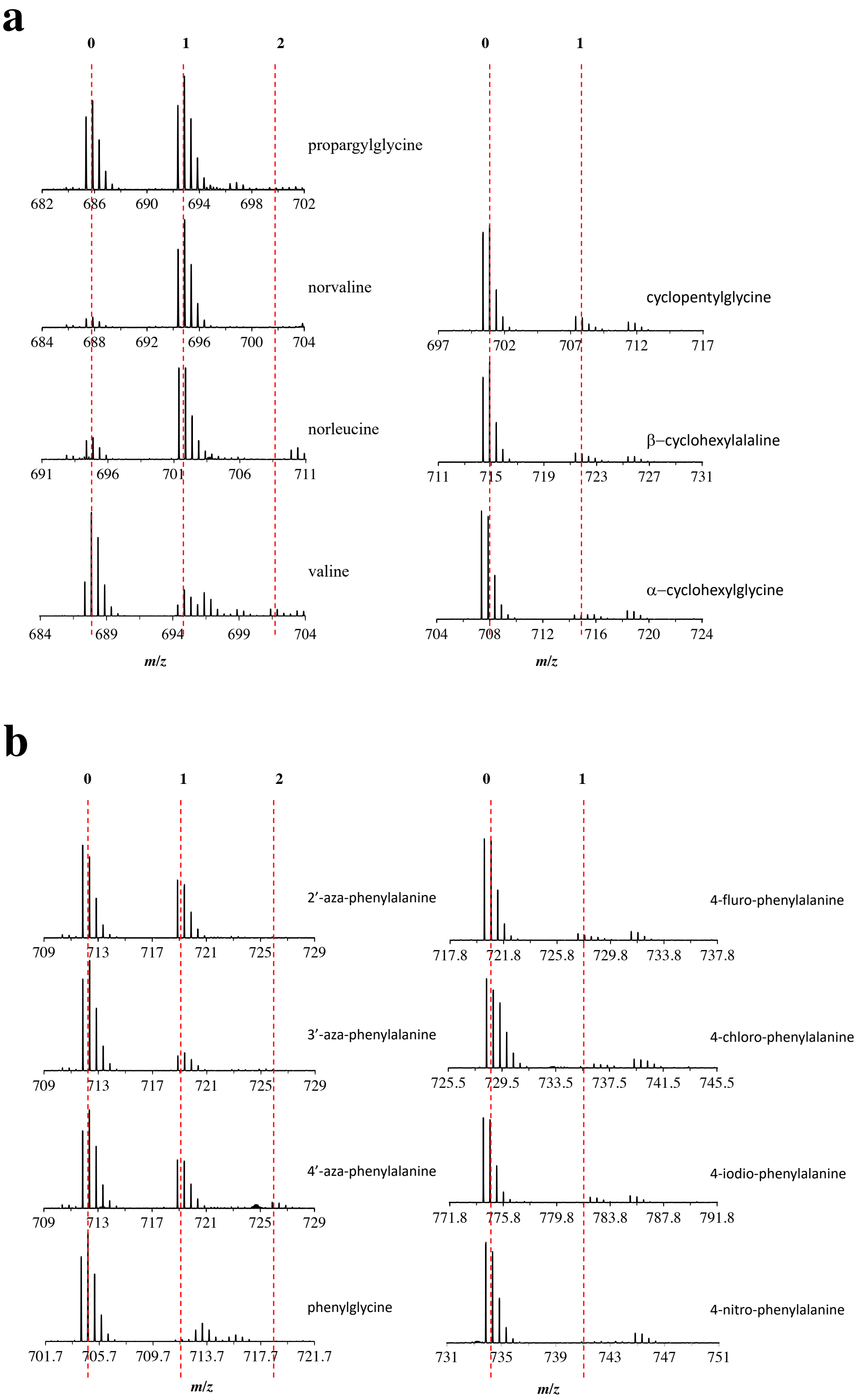

c

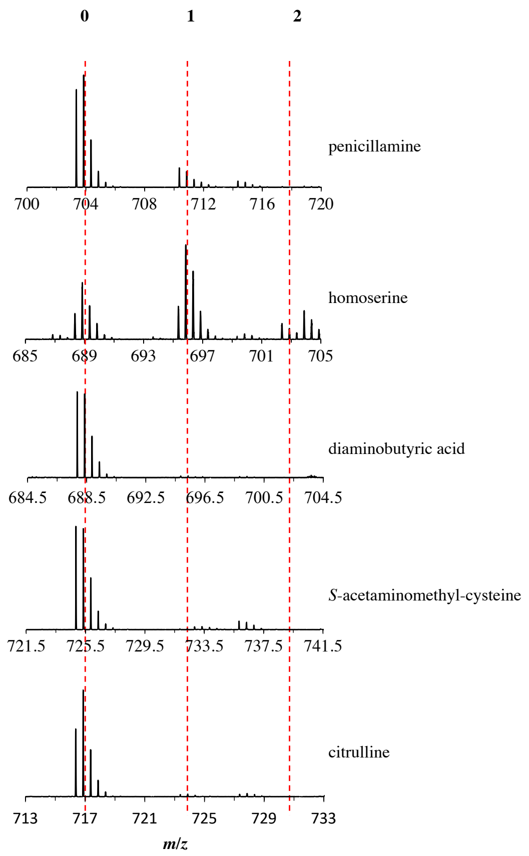
0
1
2
penicillamine
700 704 708 712 716 720
homoserine
685 689 693 697 701 705
diaminobutyric acid
684.5 688.5 692.5 696.5 700.5 704.5
S-acetaminomethyl-cysteine
721.5 725.5 729.5 733.5 737.5 741.5
citrulline
713 717 721 725 729 733
m/z

d

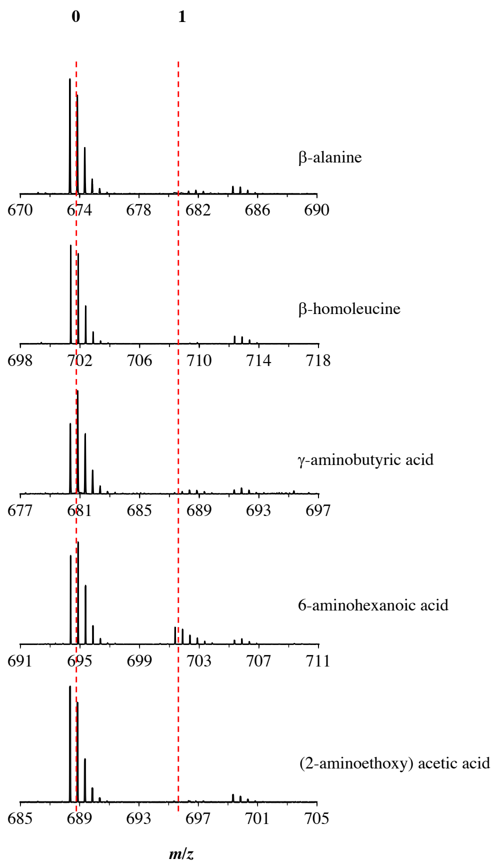
0
1
β-alanine
670 674 678 682 686 690
β-homoleucine
698 702 706 710 714 718
γ-aminobutyric acid
677 681 685 689 693 697
6-aminohexanoic acid
691 695 699 703 707 711
(2-aminoethoxy) acetic acid
685 689 693 697 701 705
m/z

e

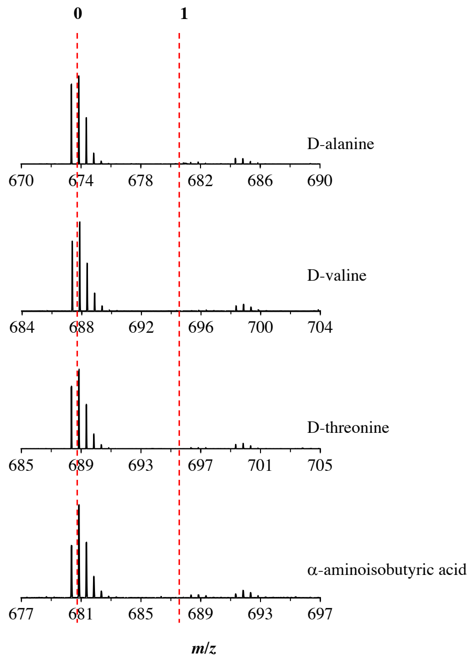
0
1
D-alanine
670 674 678 682 686 690
D-valine
684 688 692 696 700 704
D-threonine
685 689 693 697 701 705
α-aminoisobutyric acid
677 681 685 689 693 697
m/z

Figure S8. MS^2 analysis of all the proteins. All the proteins were digested with Glu-C. The methylation site is coloured in orange solid circle and the non-proteinogenic incorporation residue is coloured red. For Cys-ACM, the fragmentation also happened at the side chain which gave an apparent Cys at position 401. For Cit, fragmentation at the side chain of Cit (loss of 43 Da) is also observed at low intensity and corresponding ions were not listed. Further fragmentation of y_7 ion is observed in all the MS^2 spectrum and the MS^3 ions were labelled with black diamond.

aa

OphMA-ΔC12

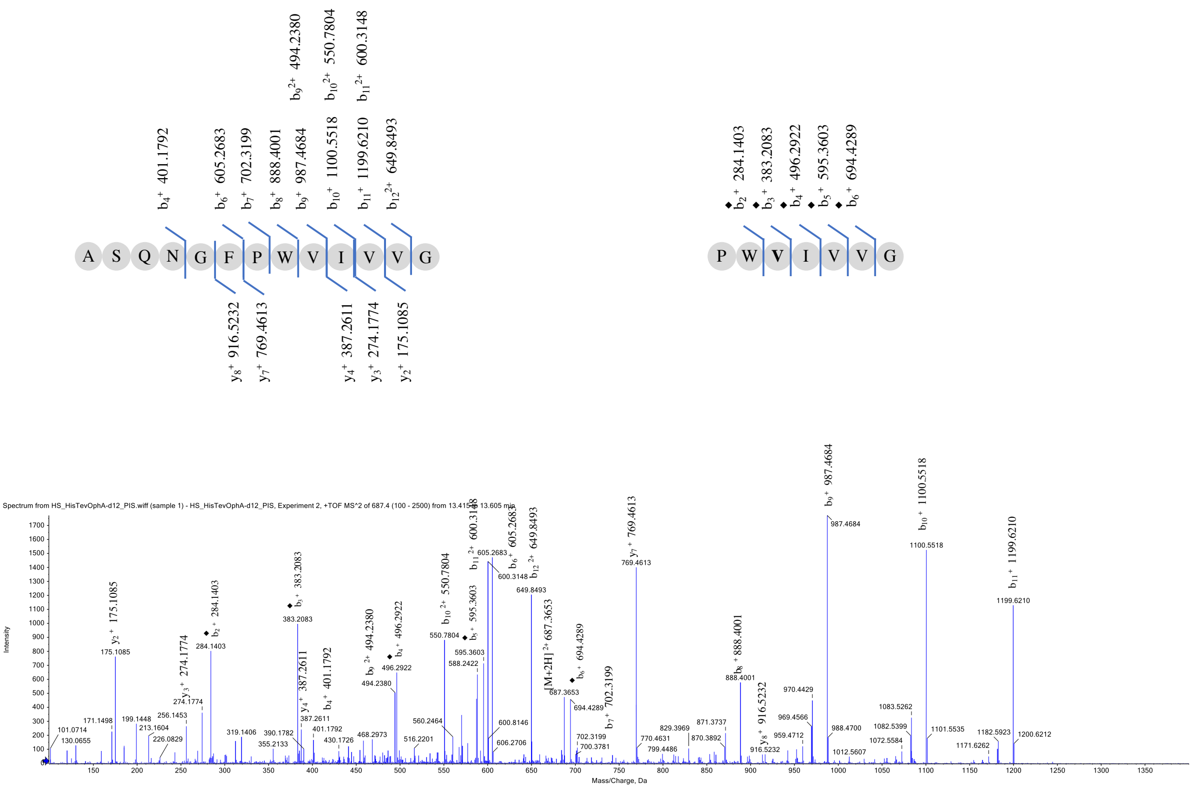

ab

OphMA-ΔC12

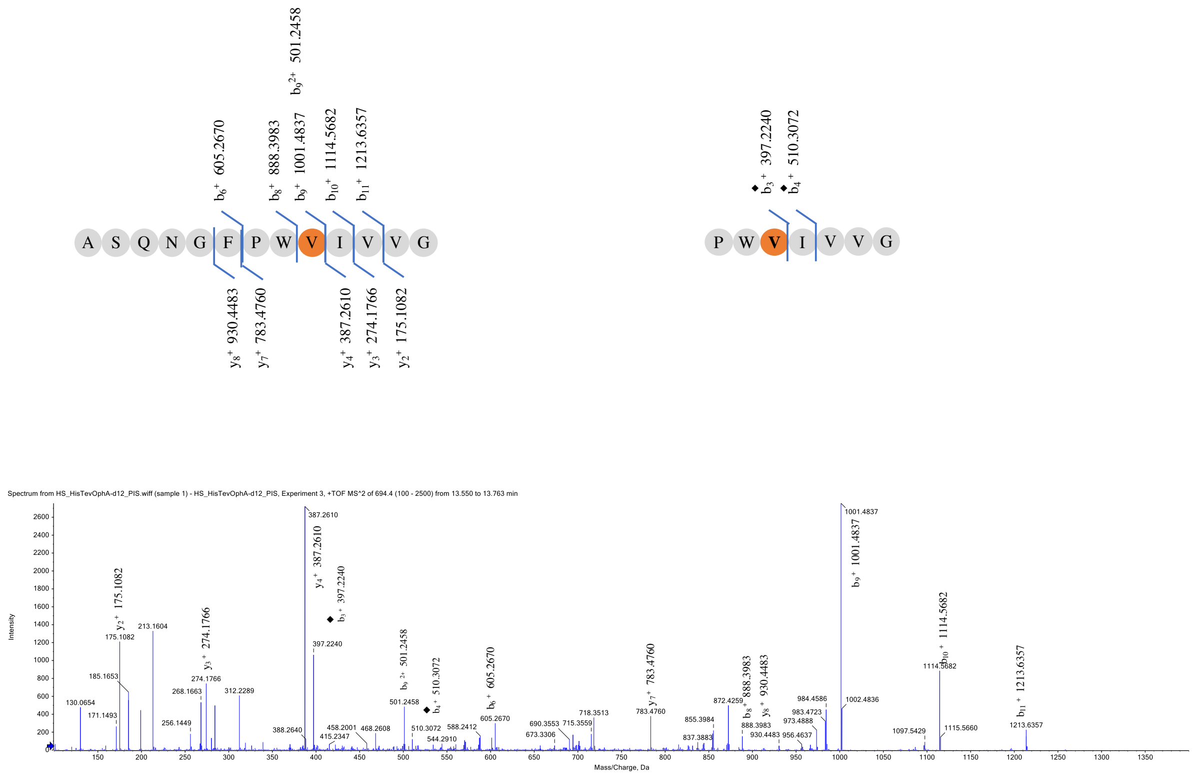

ac

OphMA-ΔC12

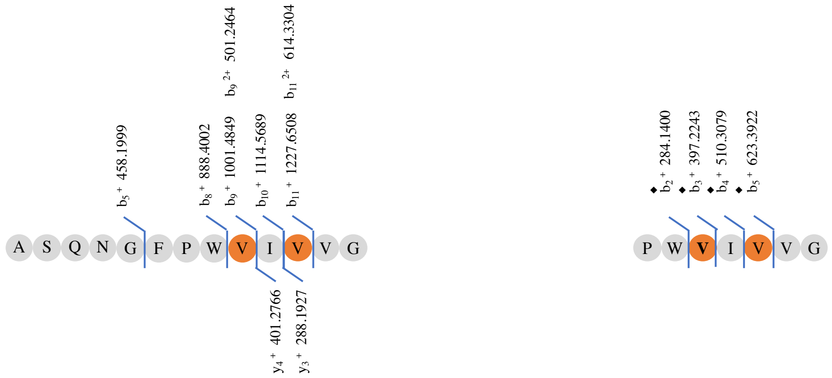

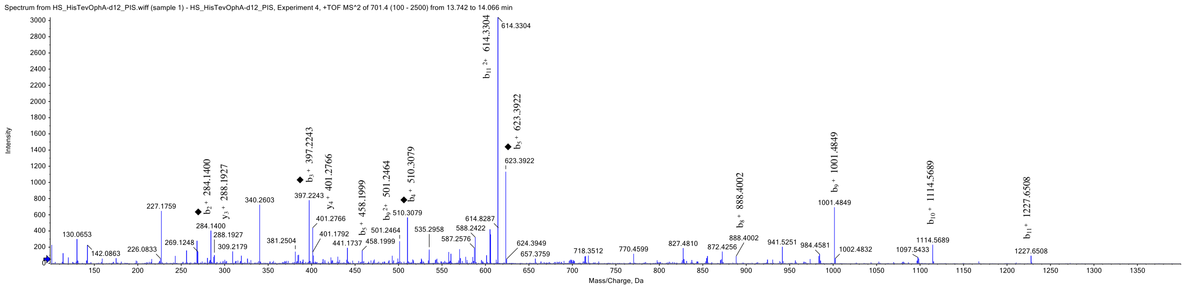

ad

OphMA-ΔC12-G390_E391insCys

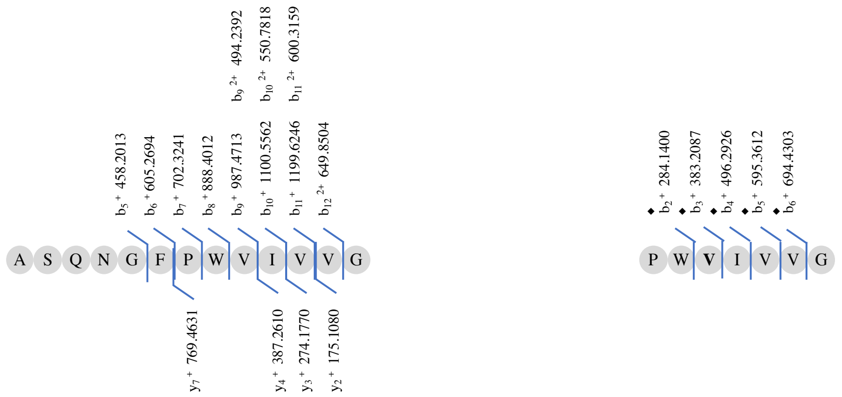

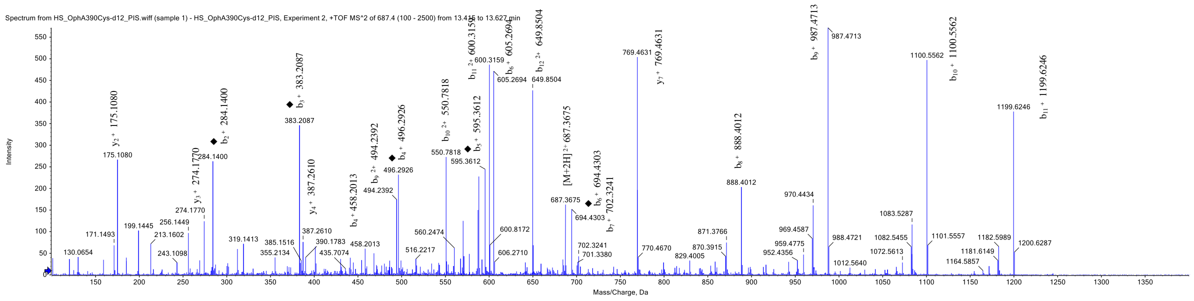

ae

OphMA-ΔC12-G390_E391insCys

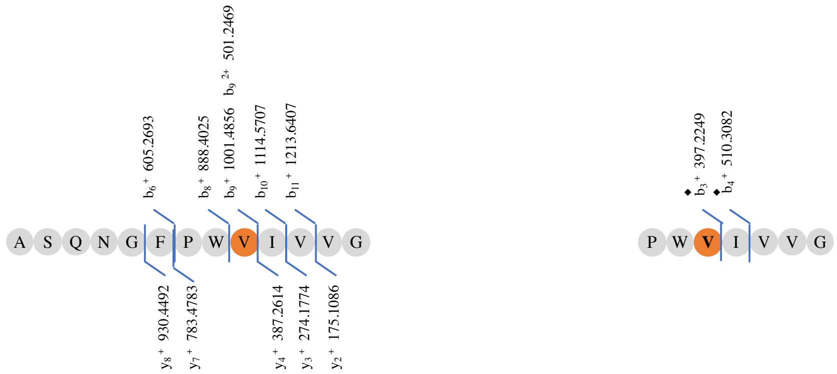

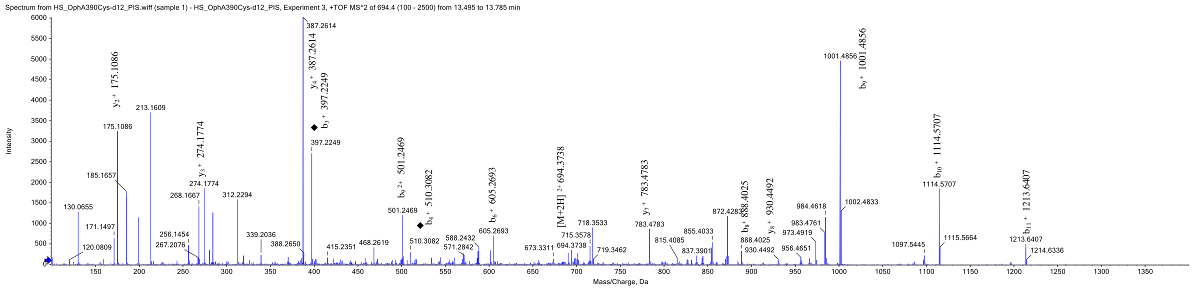

af

OphMA-ΔC12-G390_E391insCys

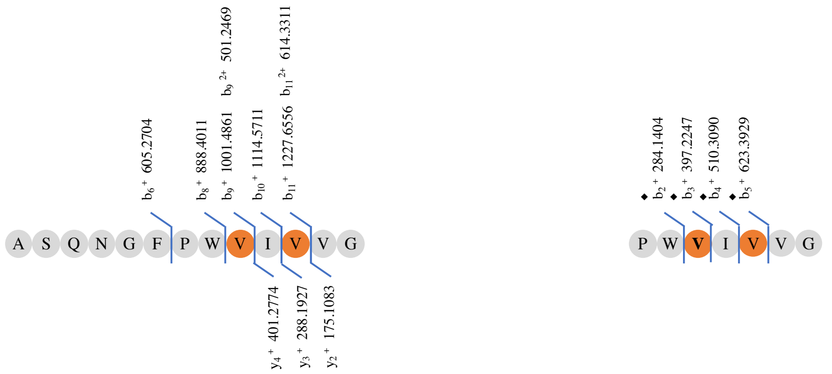

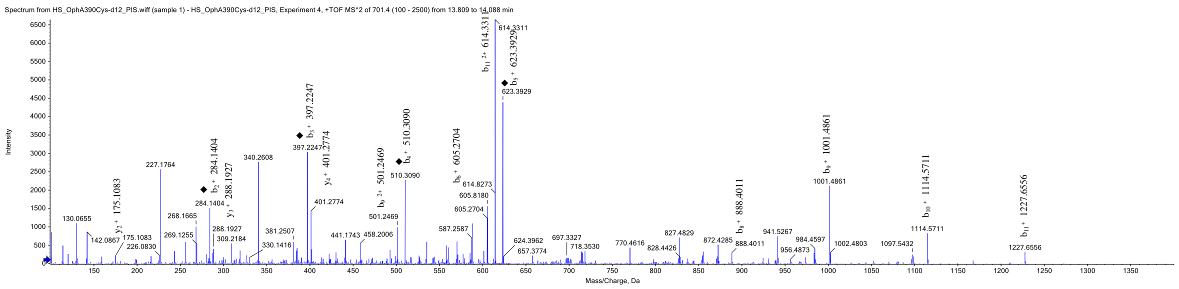

ag

fOphMA2-ΔC12-G390_E391insCys

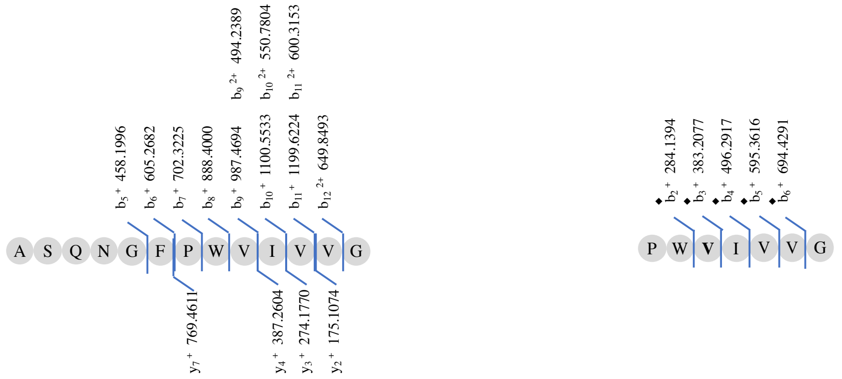

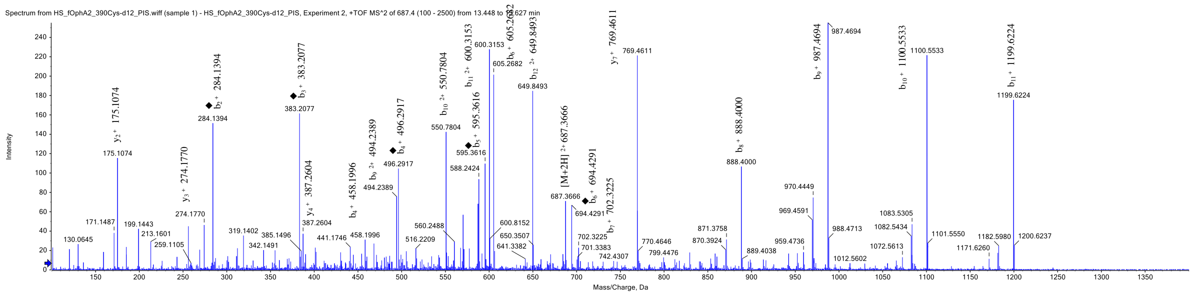

ah

fOphMA2-ΔC12-G390_E391insCys

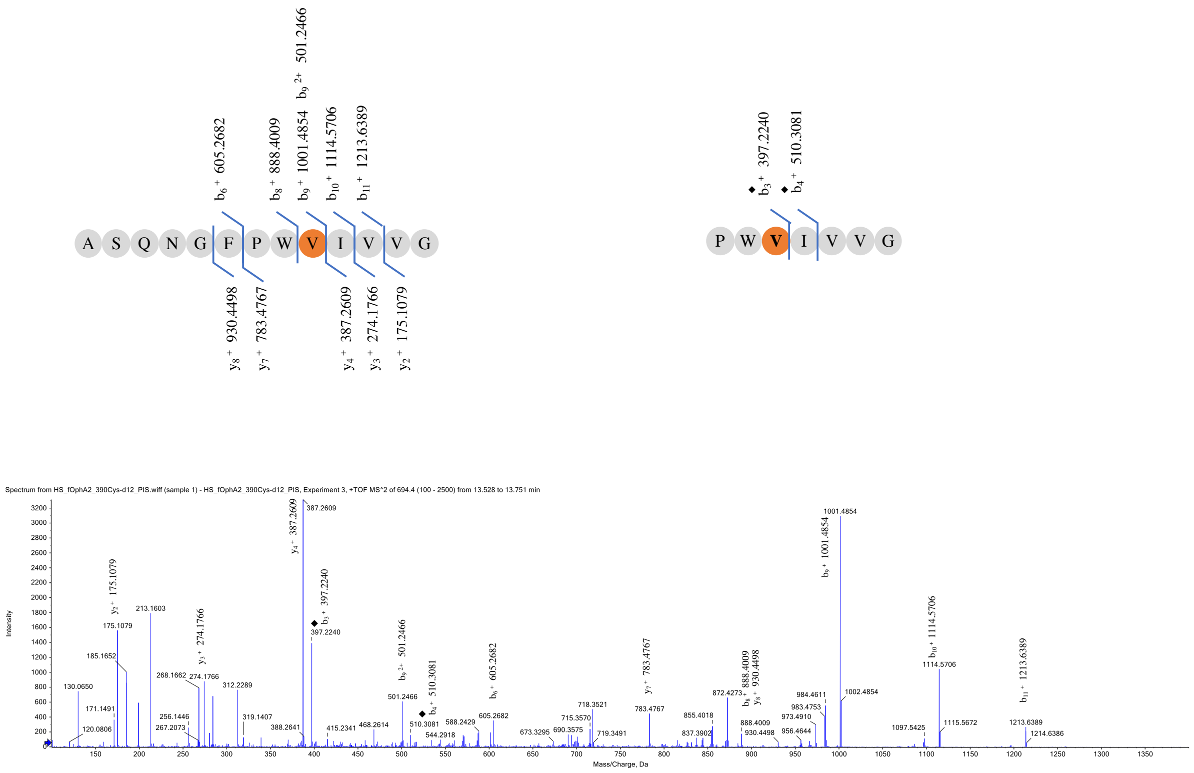

ai

fOphMA2-ΔC12-G390_E391insCys

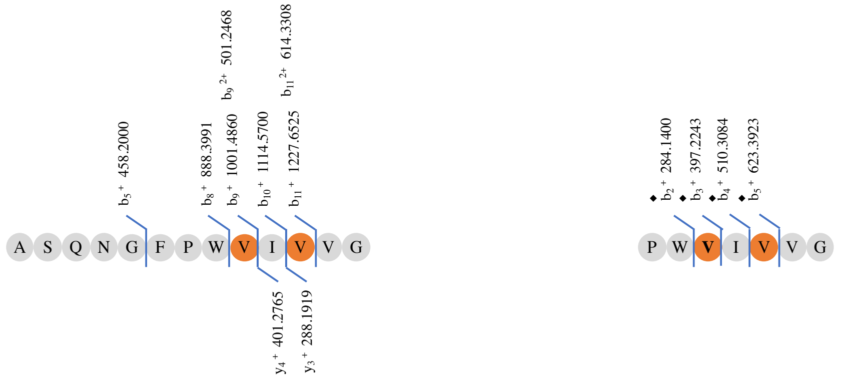

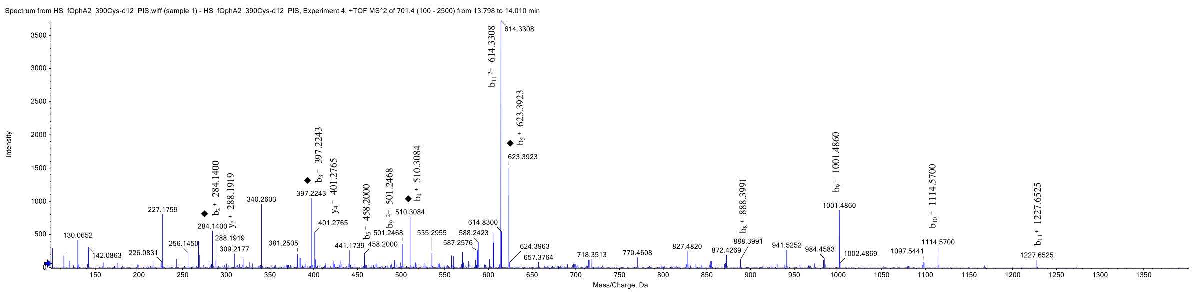

aj

fOphMA2-ΔC12-401Val

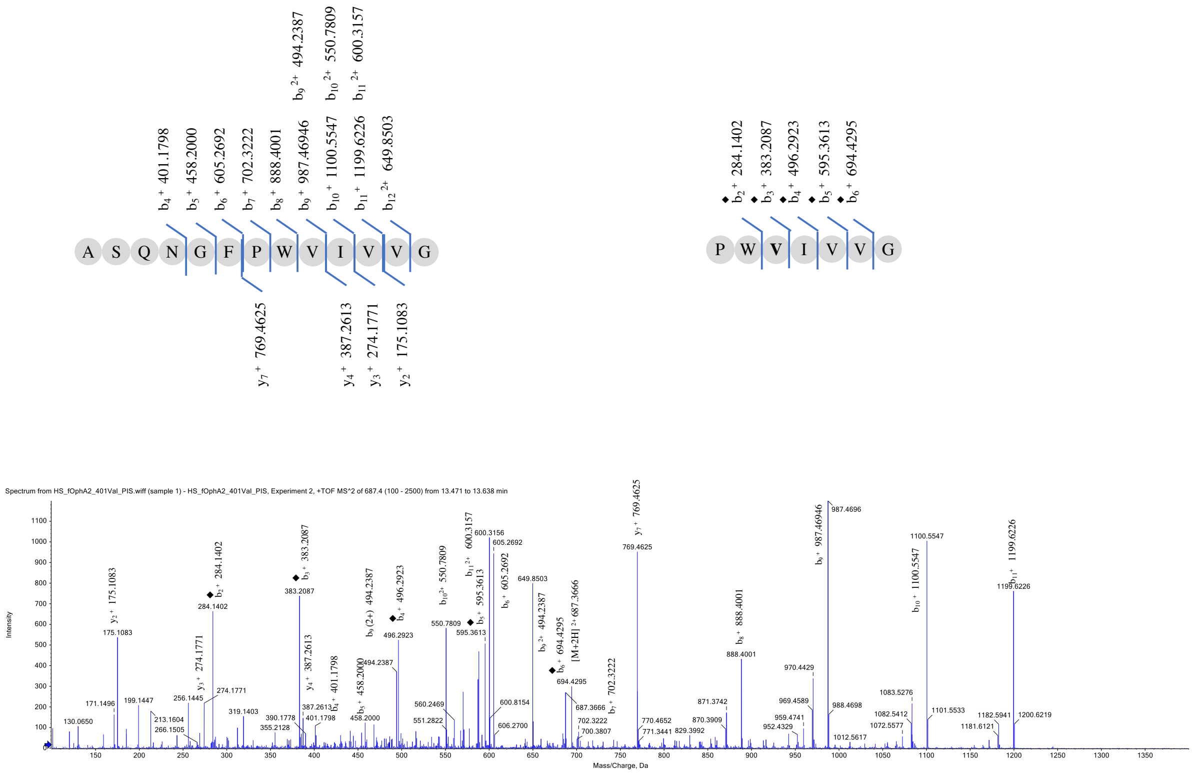

ak

fOphMA2-ΔC12-401Val

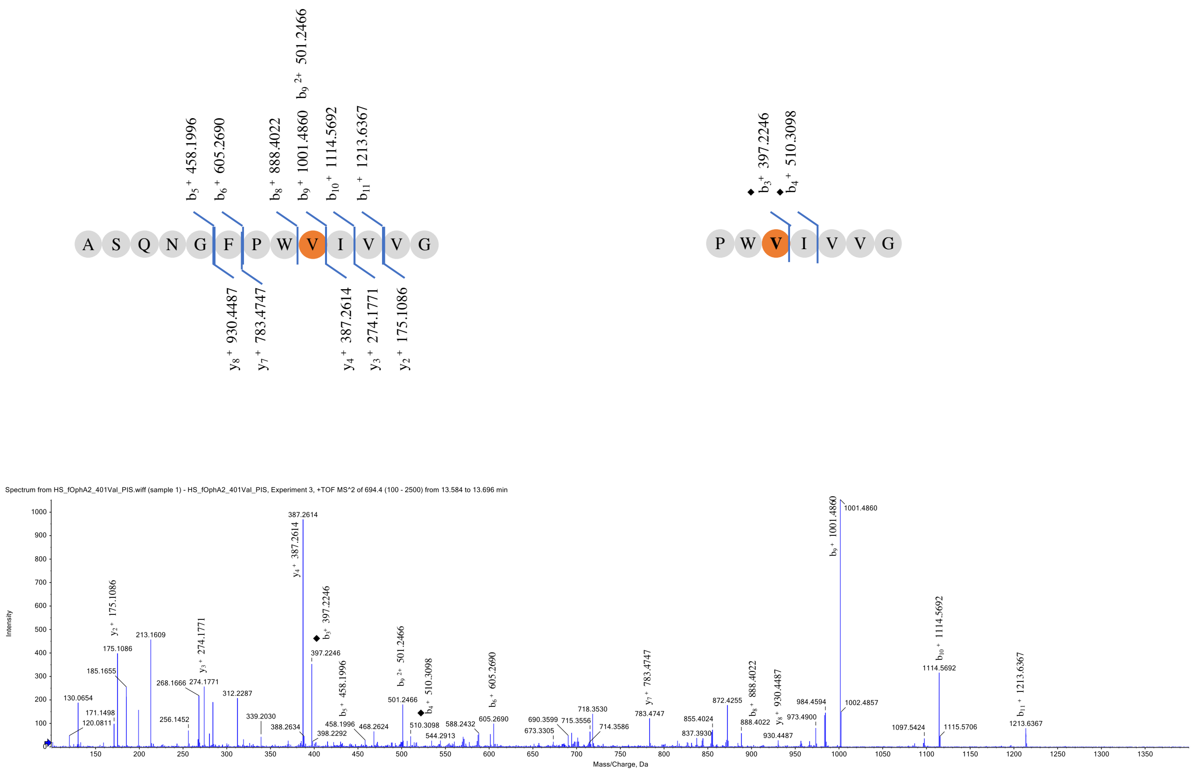

al

fOphMA2-ΔC12-401Val

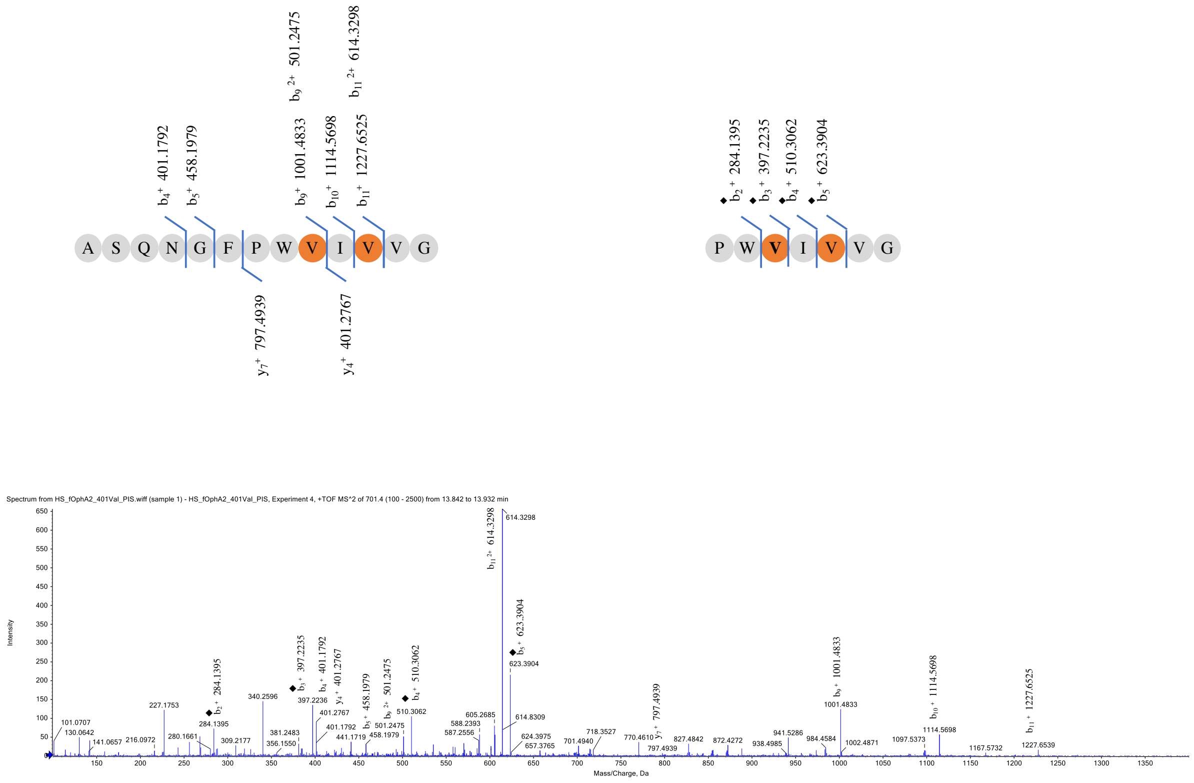

am

OphMA-401Val

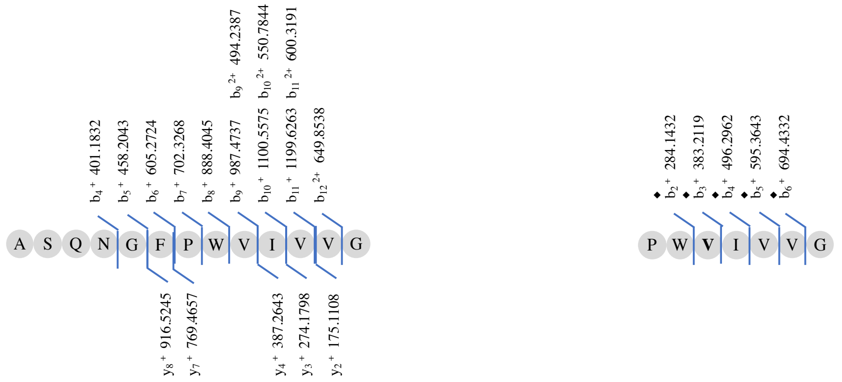

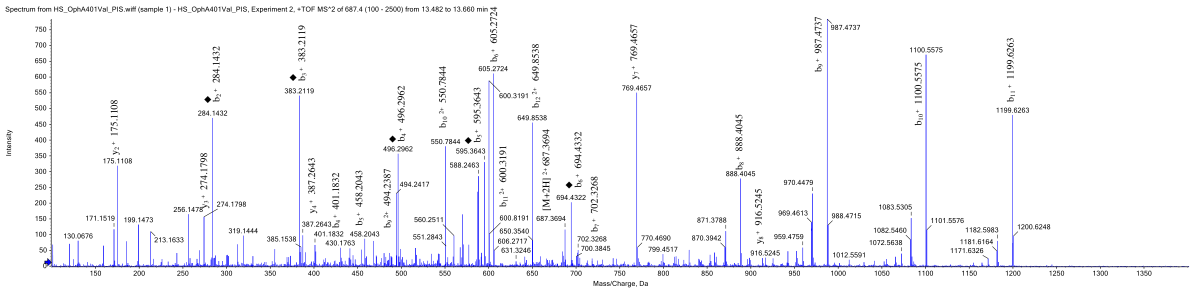

an

OphMA-ΔC12-Val401

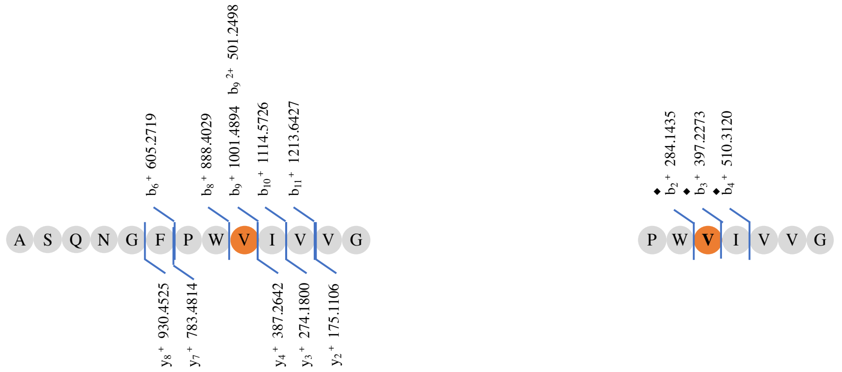

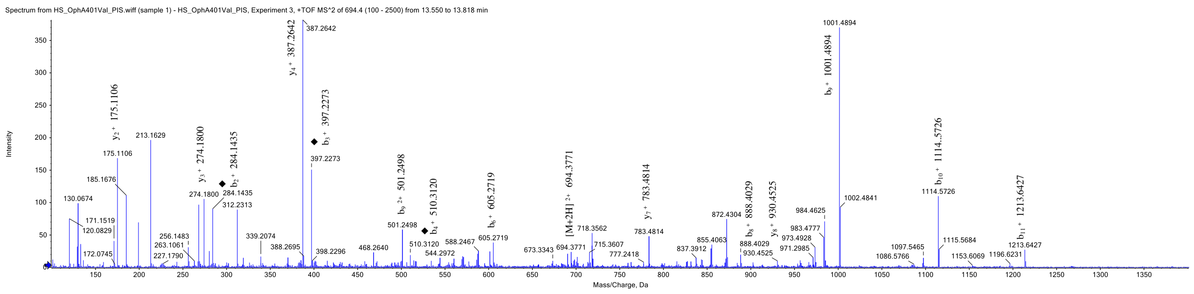

ao

fOphMA2-ΔC12-401Nva

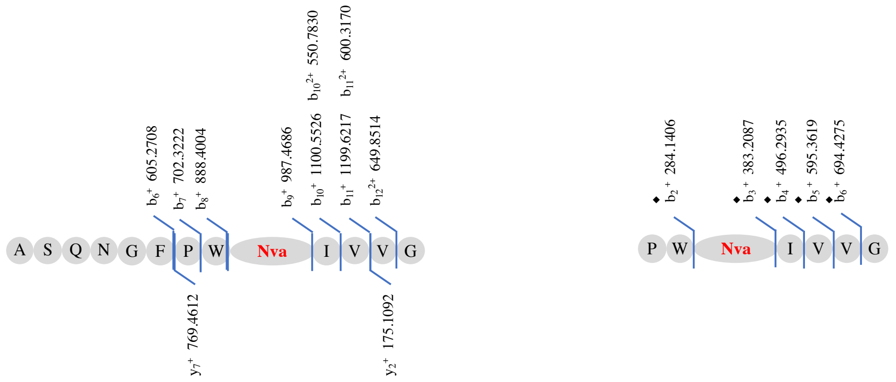

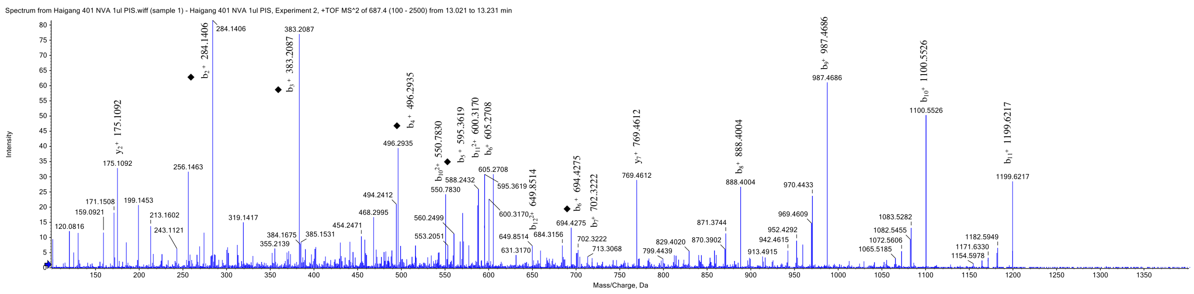

ap

fOphMA2-ΔC12-401Nva

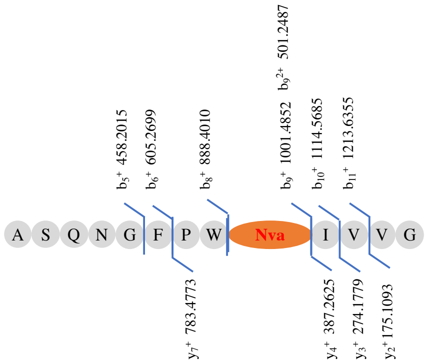

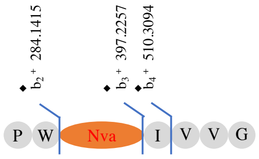

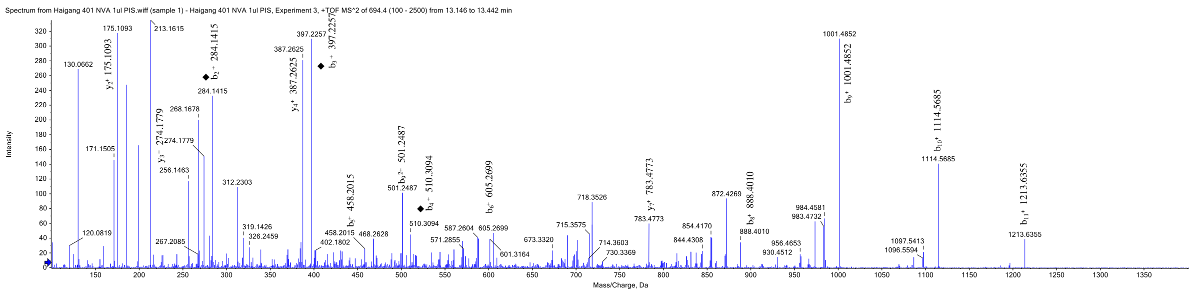

aq

fOphMA2-ΔC12-401Nle

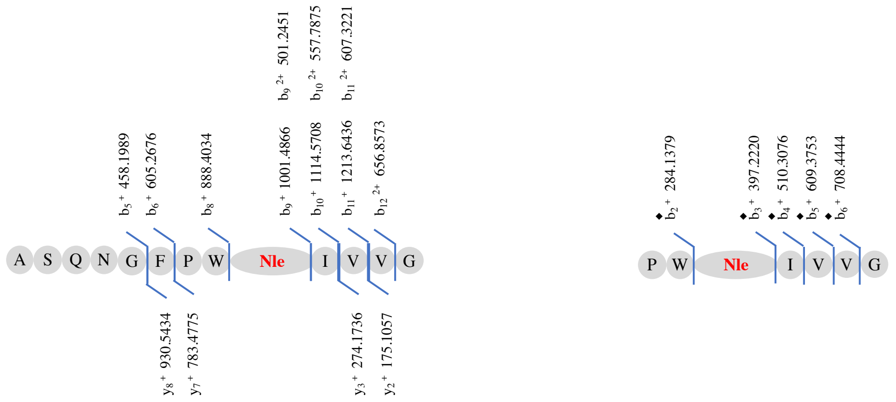

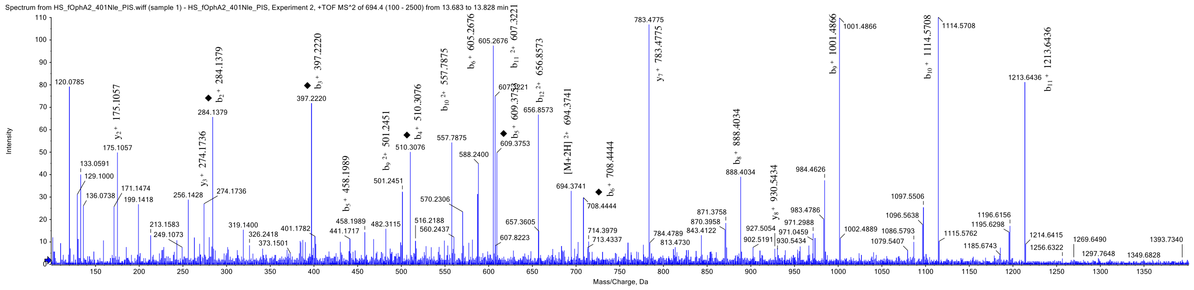

ar

fOphMA2-ΔC12-401Nle

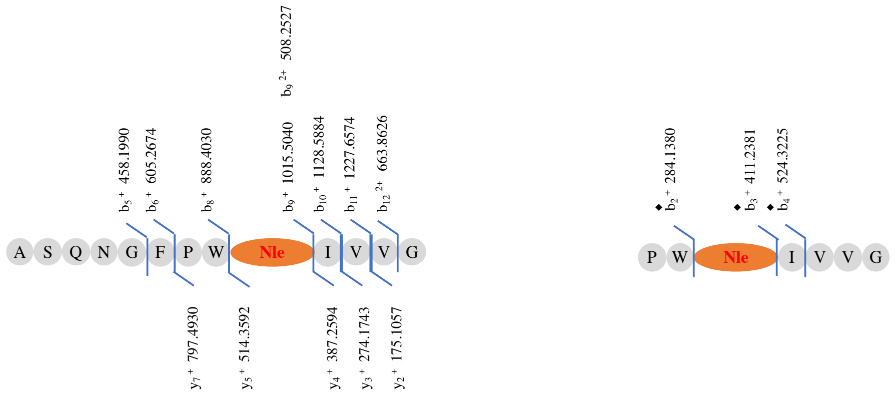

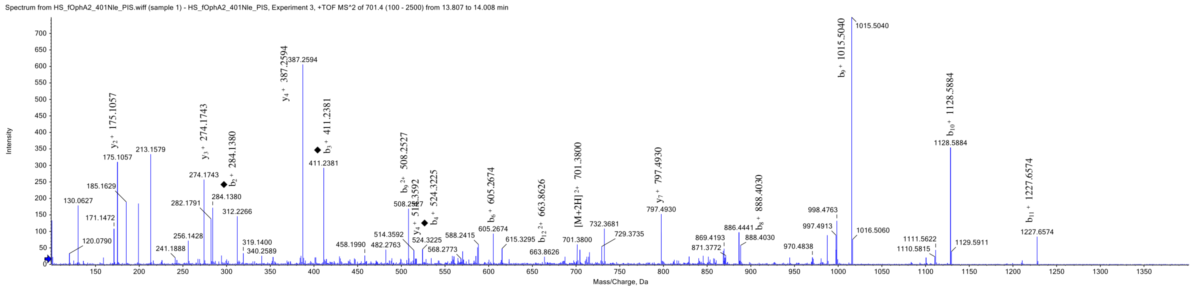

as

fOphMA2-ΔC12-401Pra

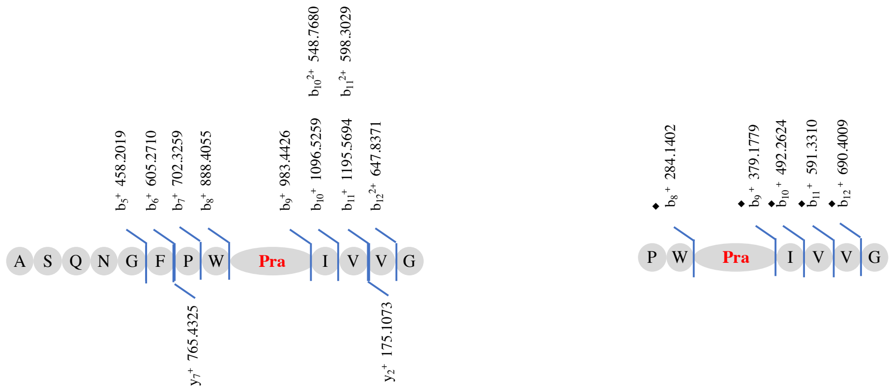

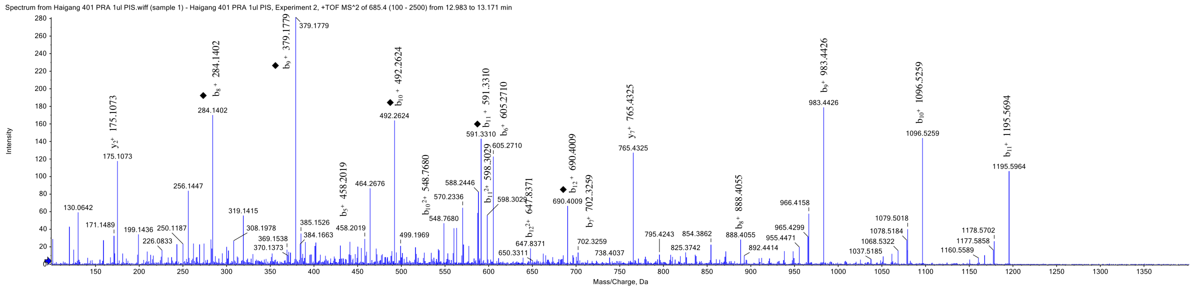

at

fOphMA2-ΔC12-401Pra

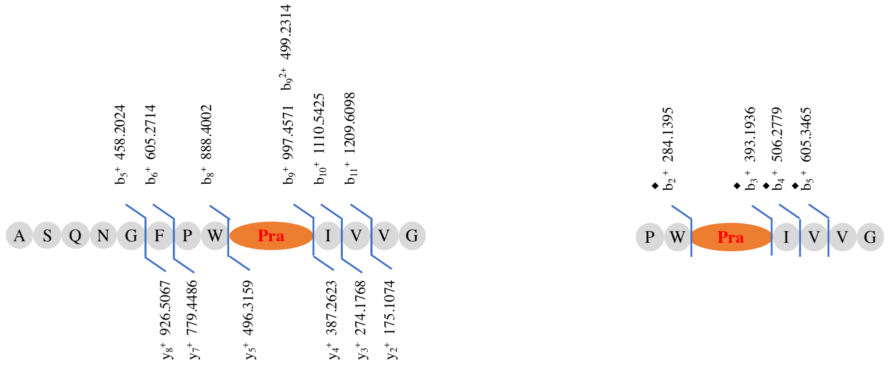

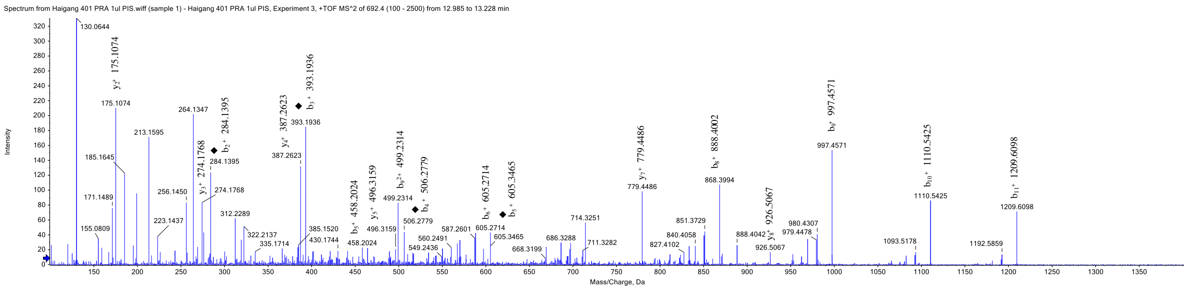

au

fOphMA2-ΔC12-401Pra

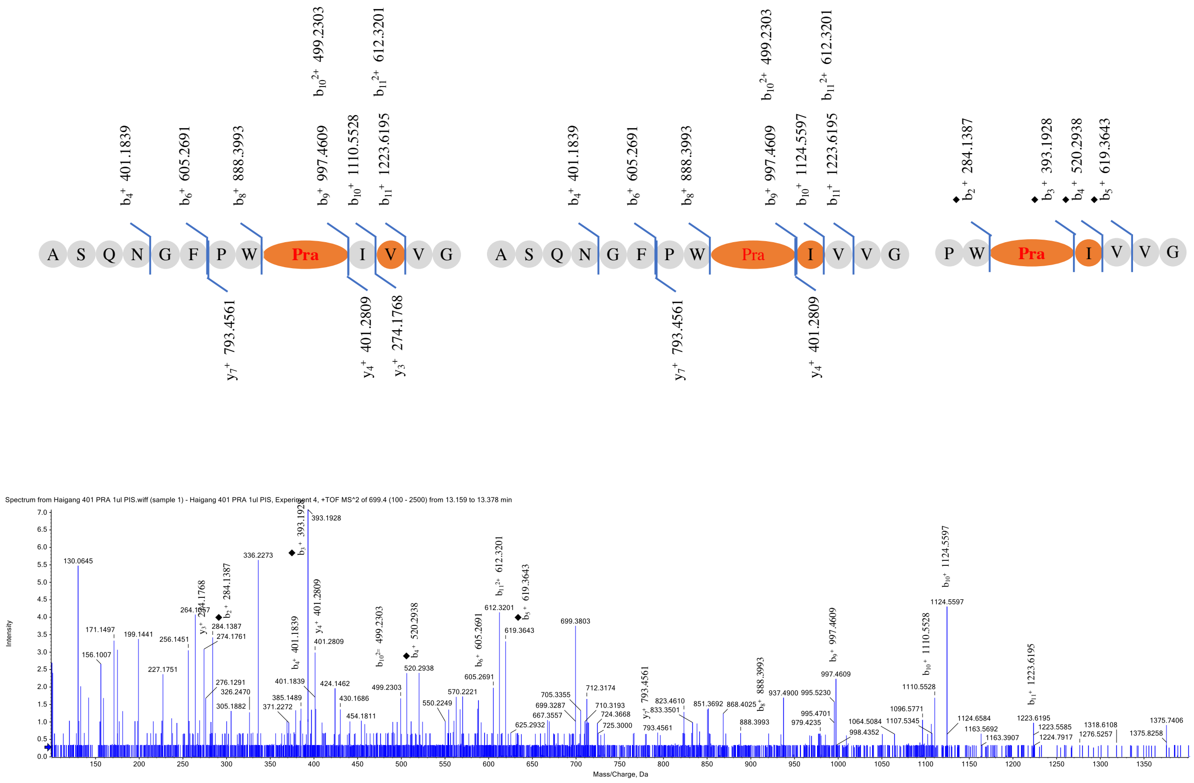

av

fOphMA2-ΔC12-401Cha

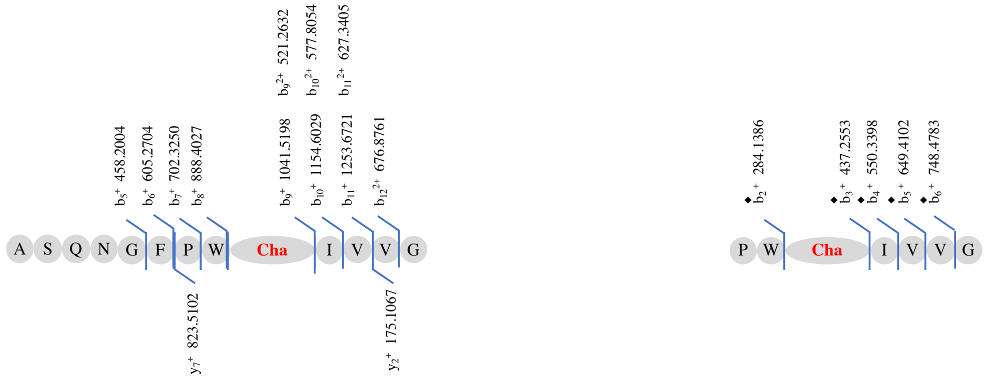

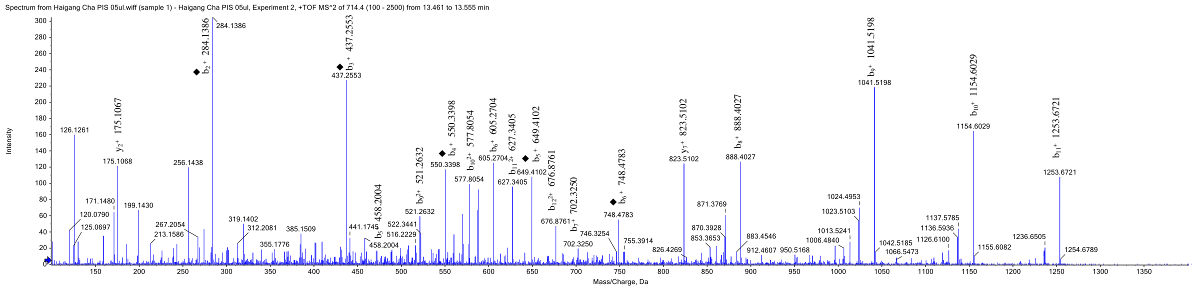

aw

fOphMA2-ΔC12-401Cha

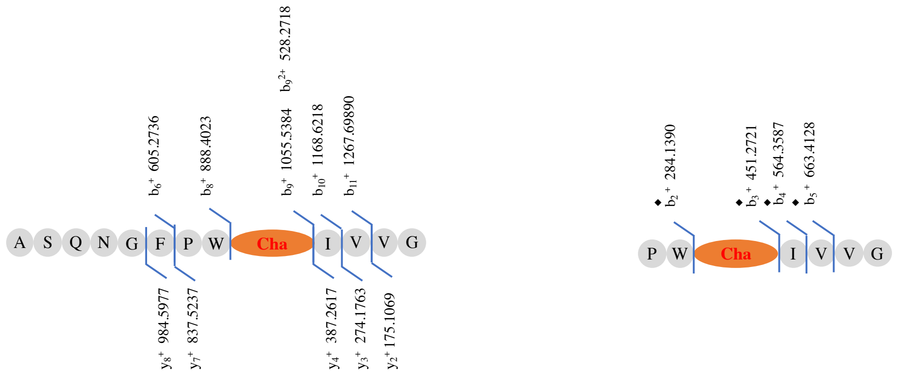

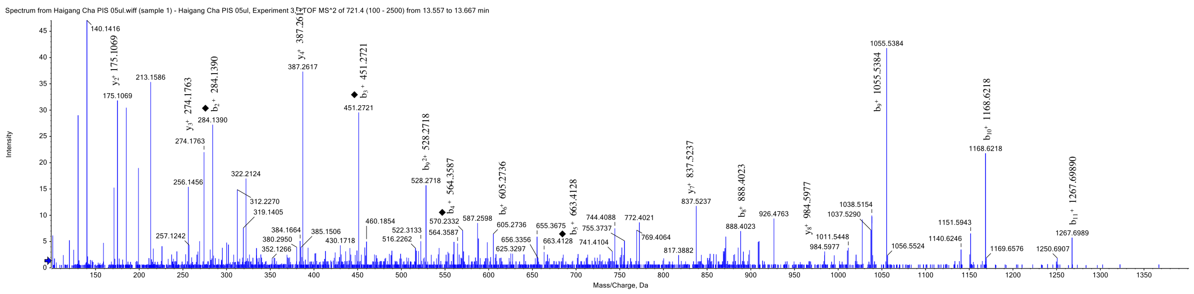

ax

fOphMA2-ΔC12-401Chg

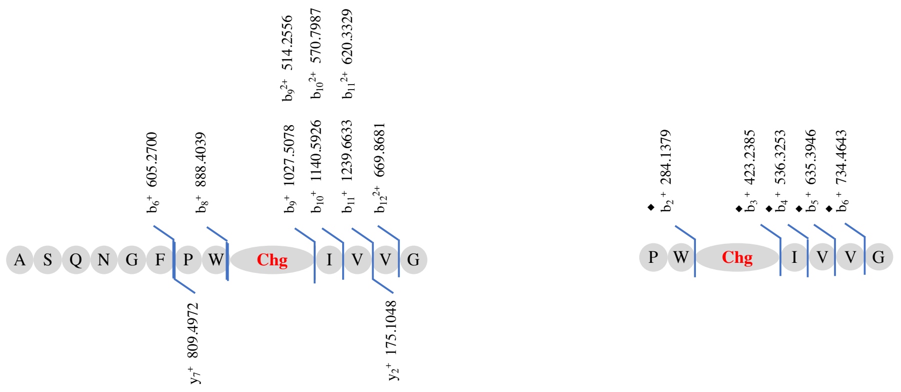

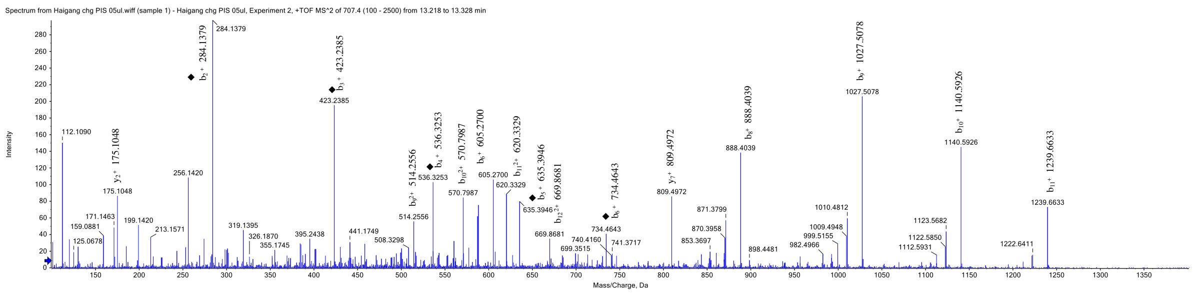

ay

fOphMA2-ΔC12-401Chg

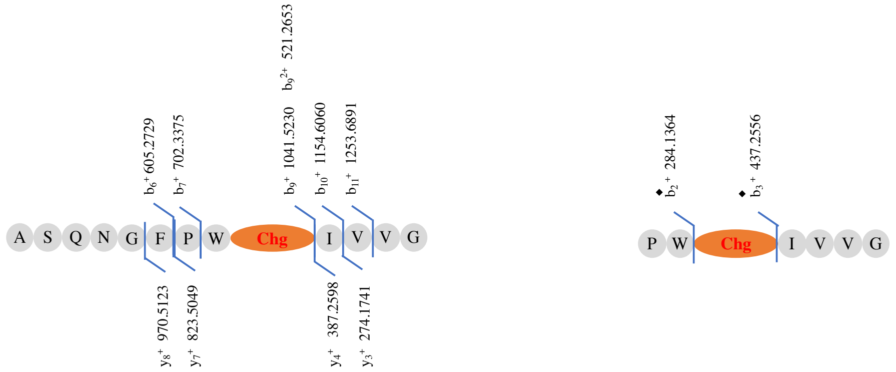

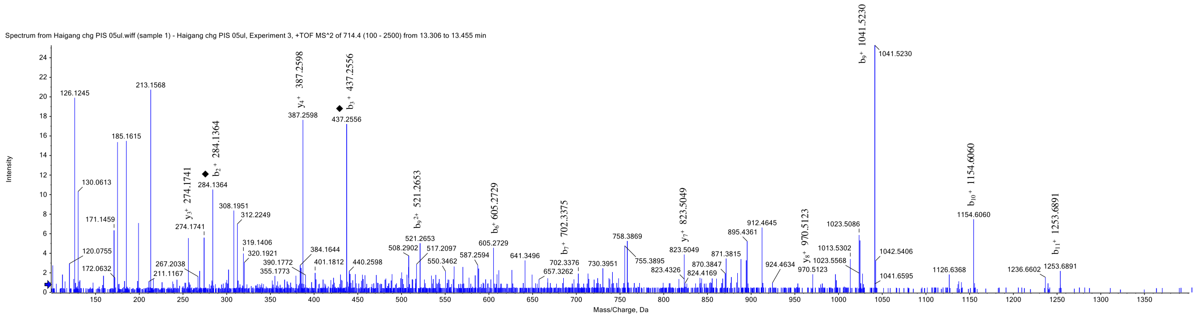

az

fOphMA2-ΔC12-401Cpg

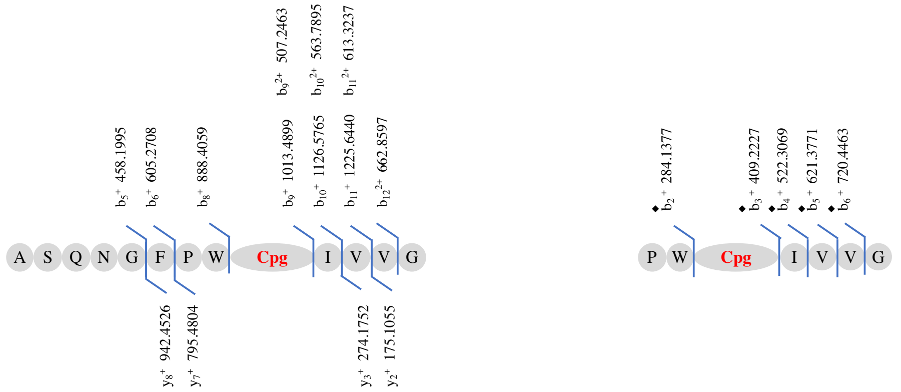

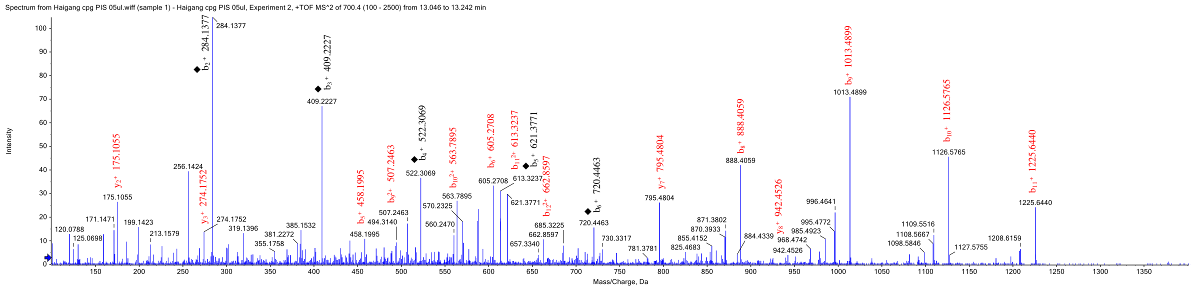

ba

fOphMA2-ΔC12-401Cpg

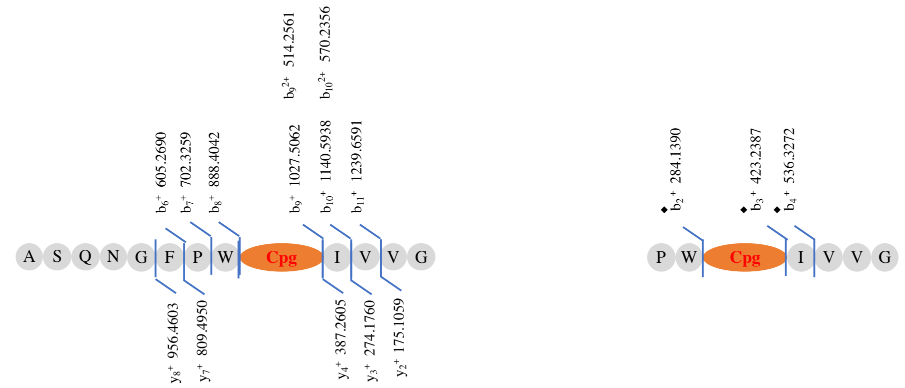

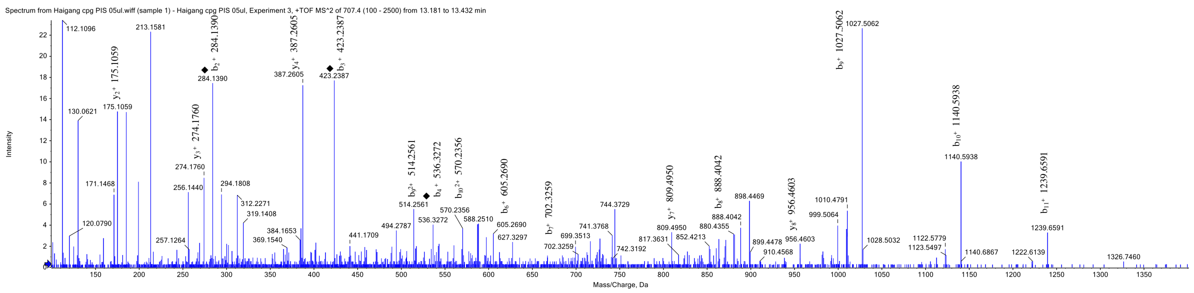

bb

fOphMA2-ΔC12-401Cpg

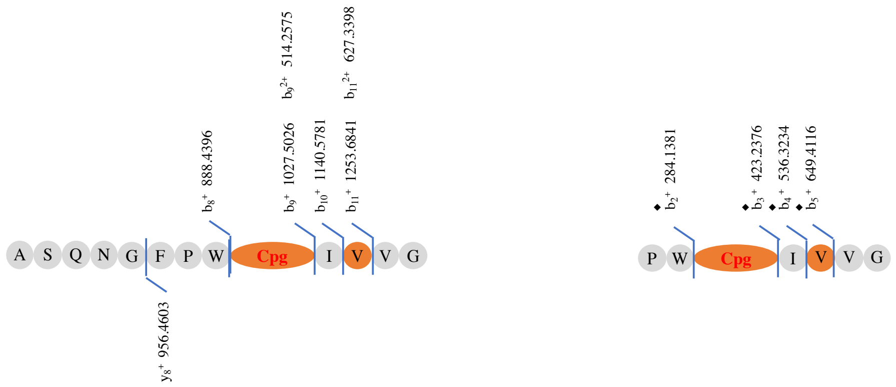

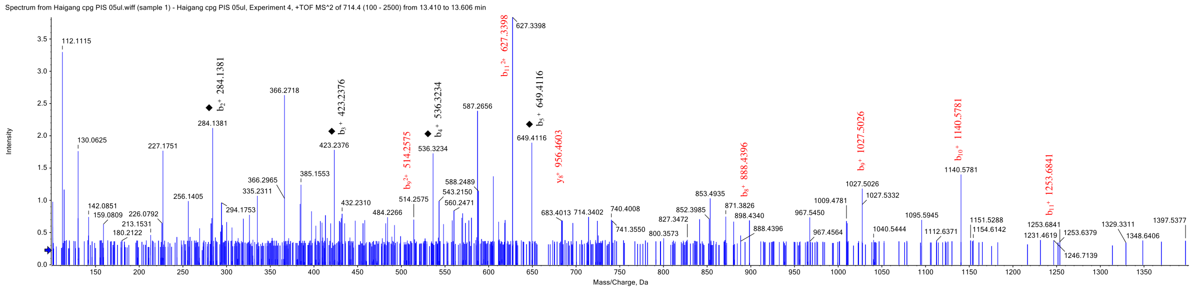

bc

fOphMA2-ΔC12-401Phg

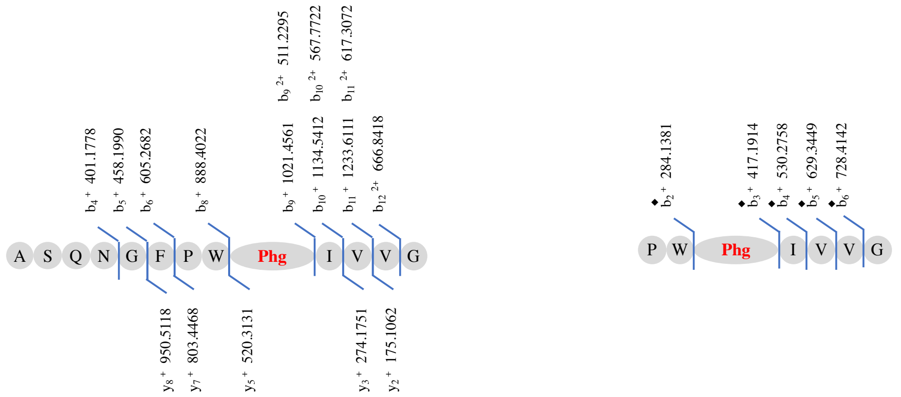

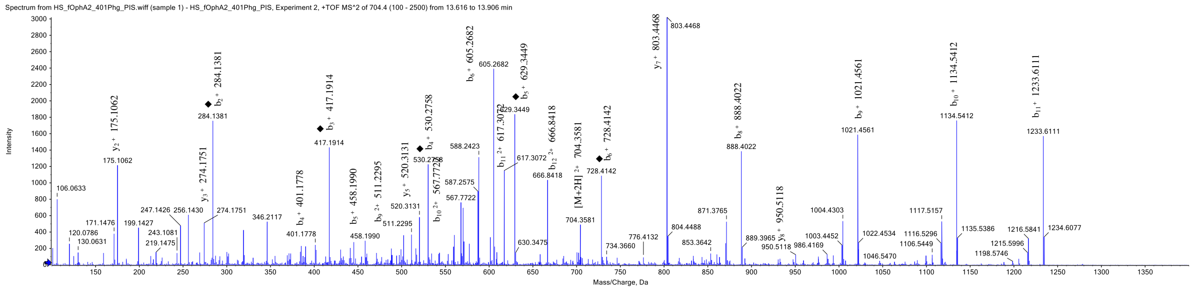

bd

fOphMA2-ΔC12-401Phg

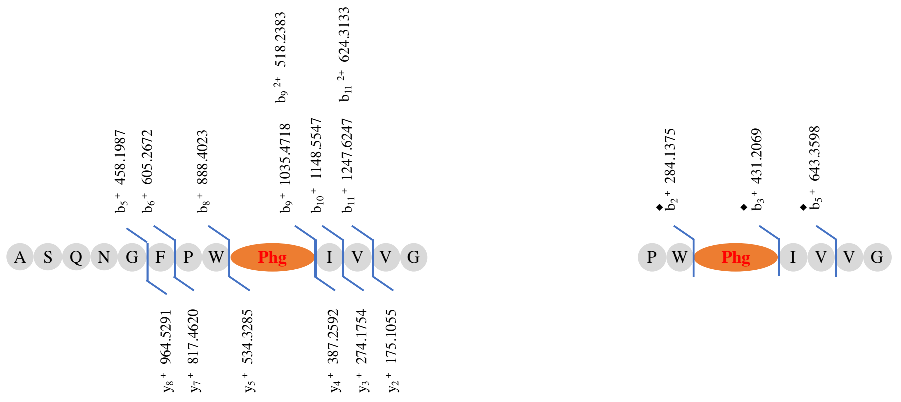

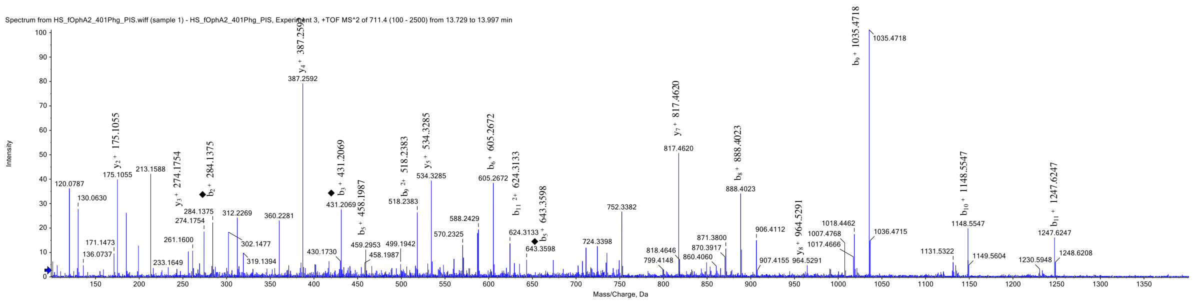

be

fOphMA2-ΔC12-401-2-Pal

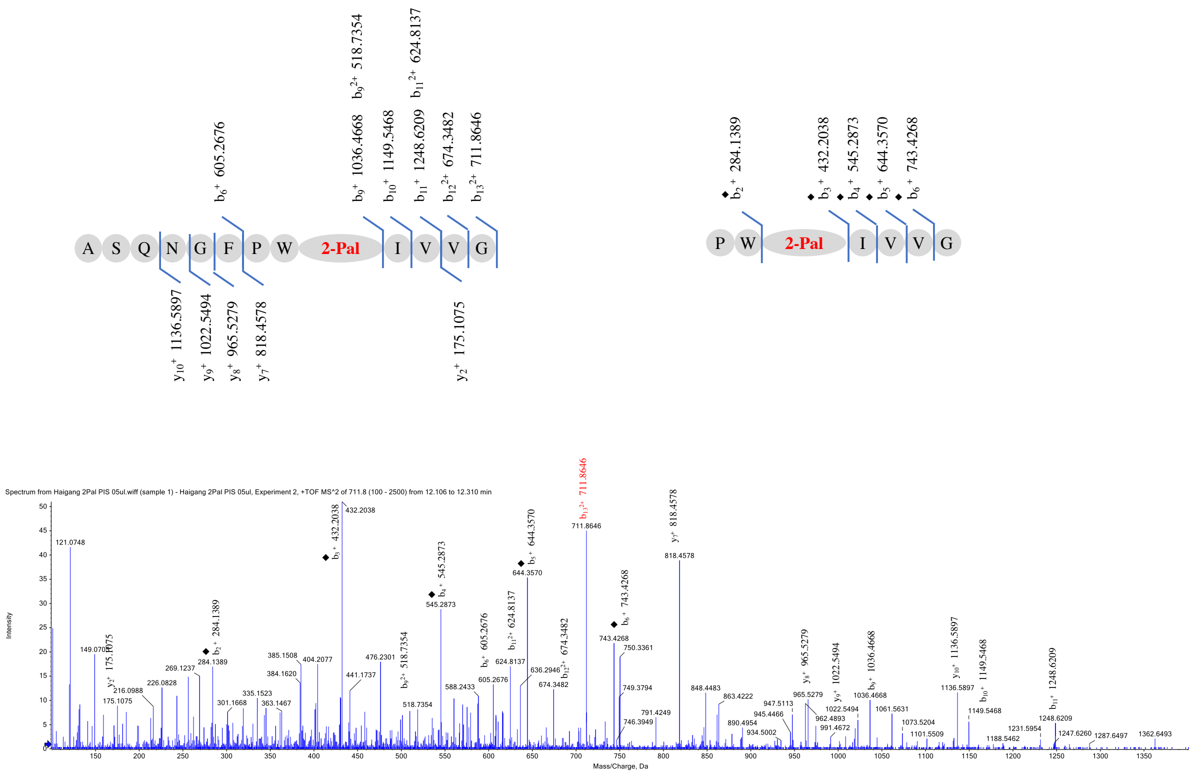

bf

fOphMA2-ΔC12-401-2-Pal

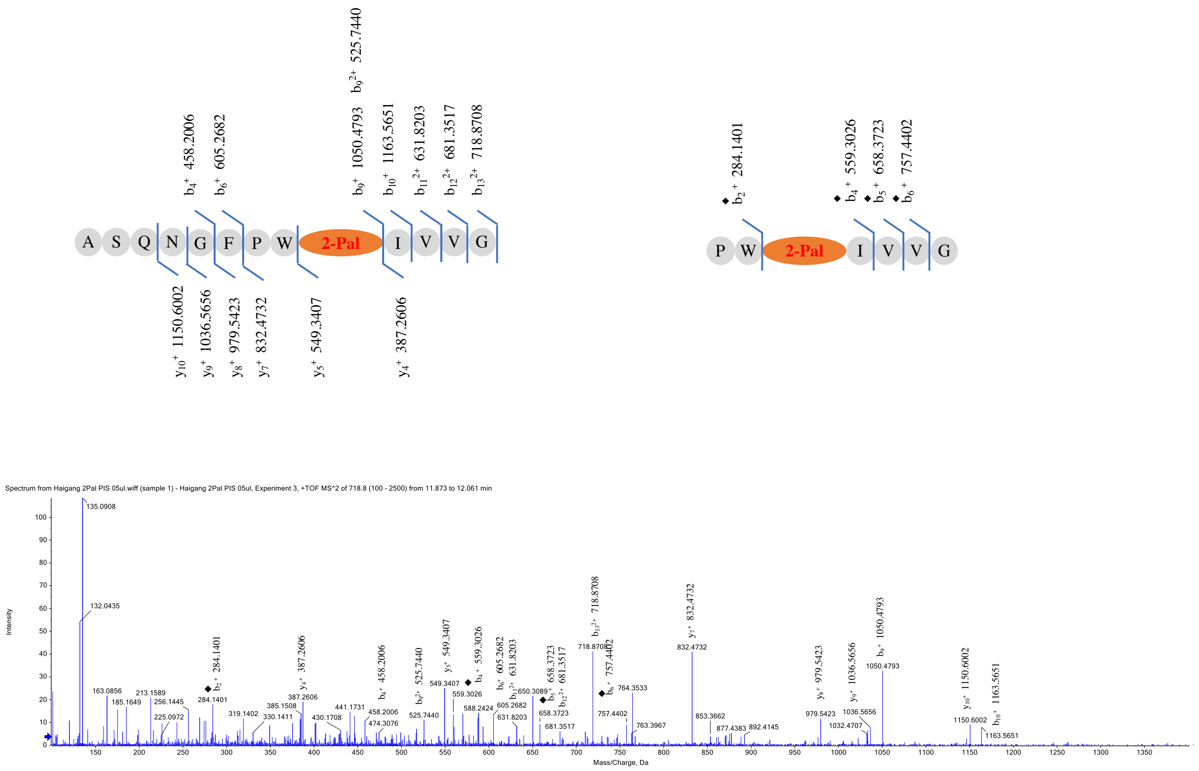

bg

fOphMA2-ΔC12-401-3-Pal

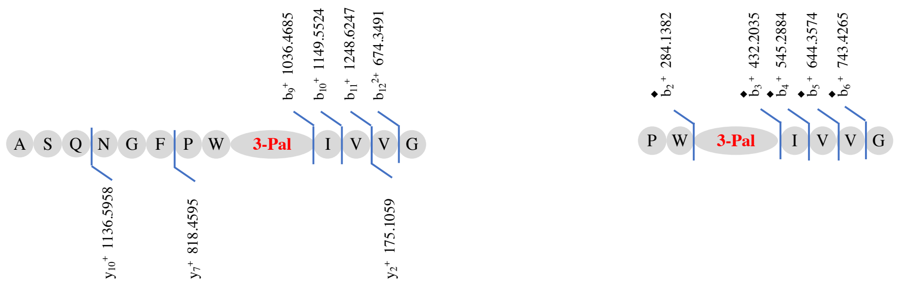

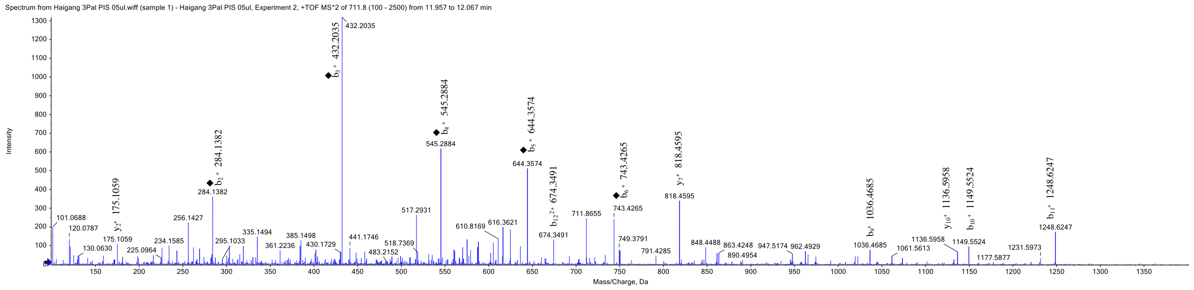

bh

fOphMA2-ΔC12-401-3-Pal

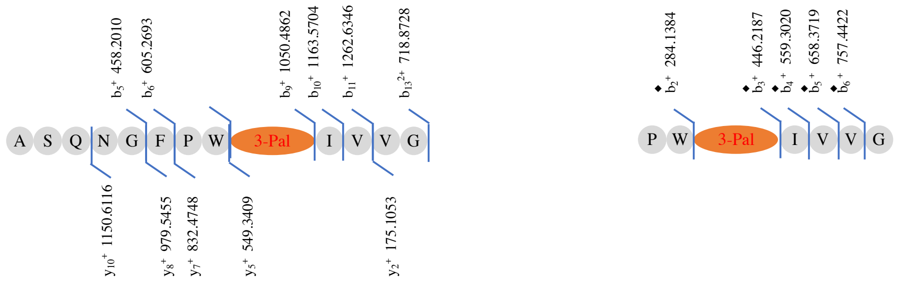

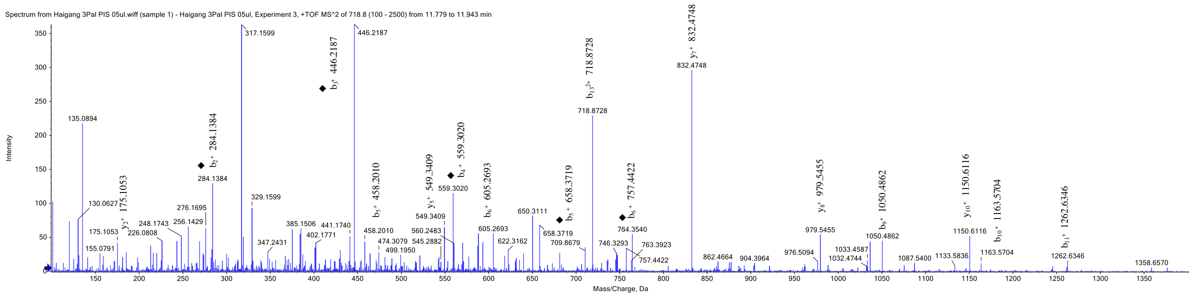

bi

fOphMA2-ΔC12-401-4-Pal

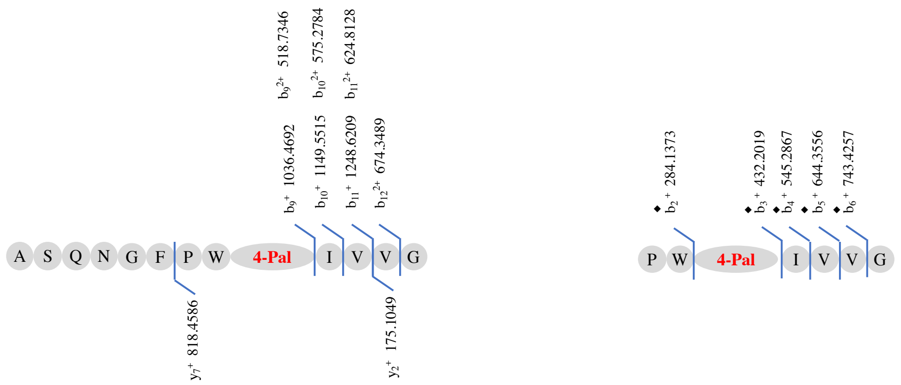

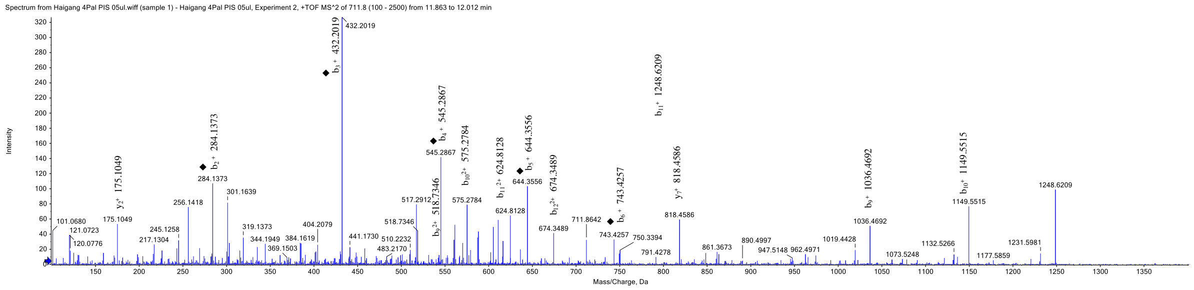

bj

fOphMA2-ΔC12-401-4-Pal

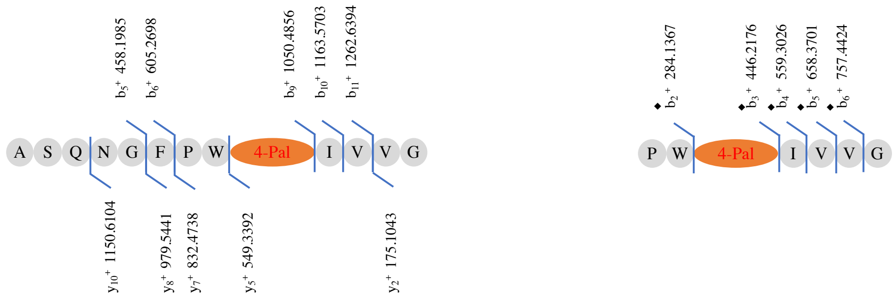

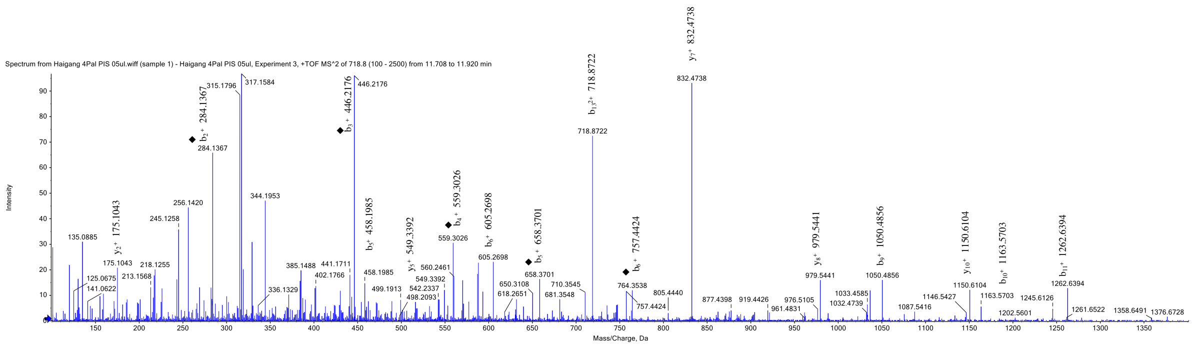

bk

fOphMA2-ΔC12-401-4-Pal

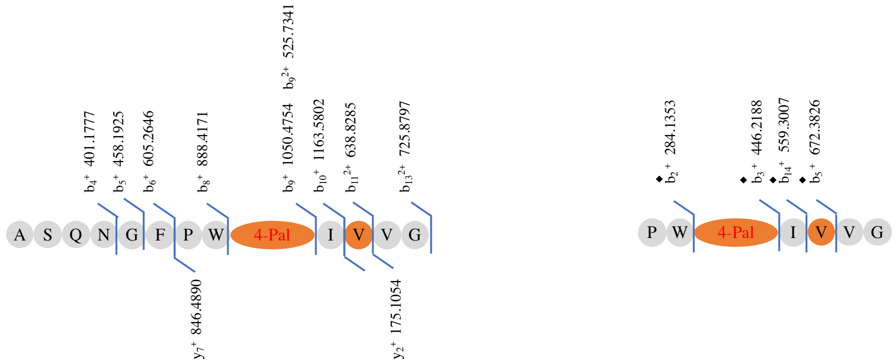

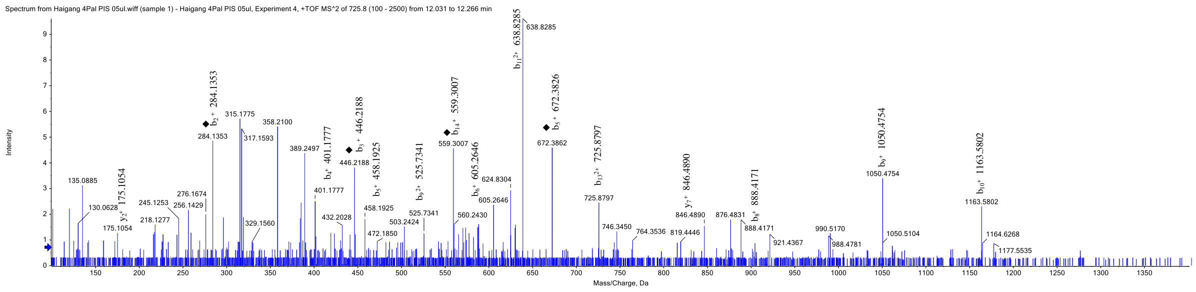

bl

fOphMA2-ΔC12-401-4F-Phe

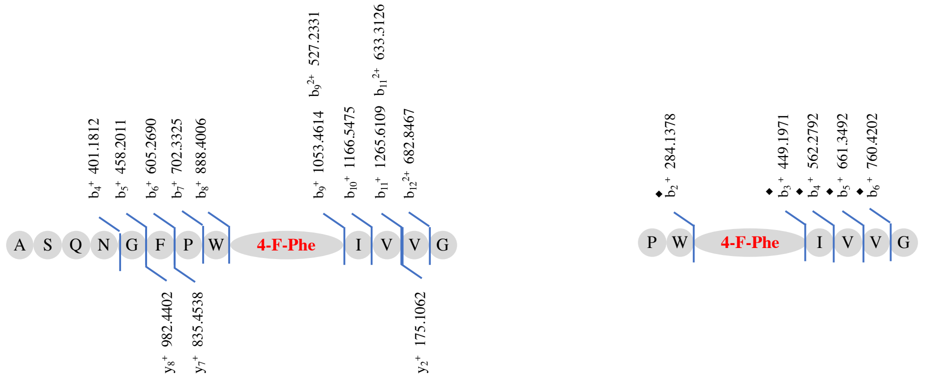

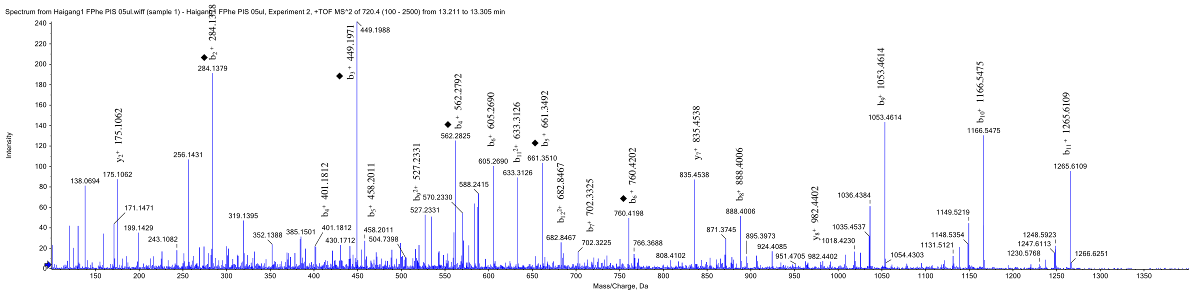

bm

fOphMA2-ΔC12-401-4F-Phe

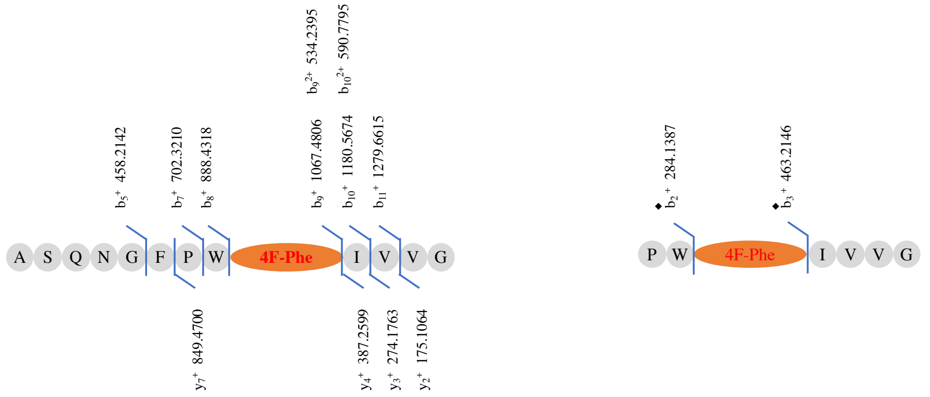

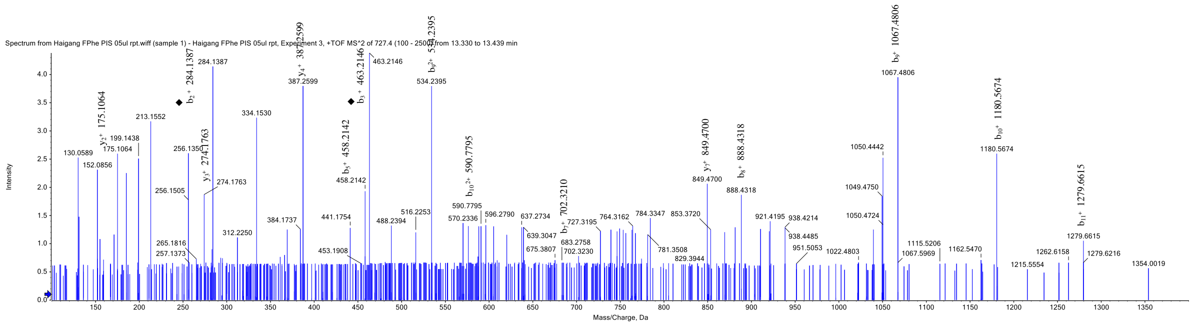

bn

fOphMA2-ΔC12-401-4Cl-Phe

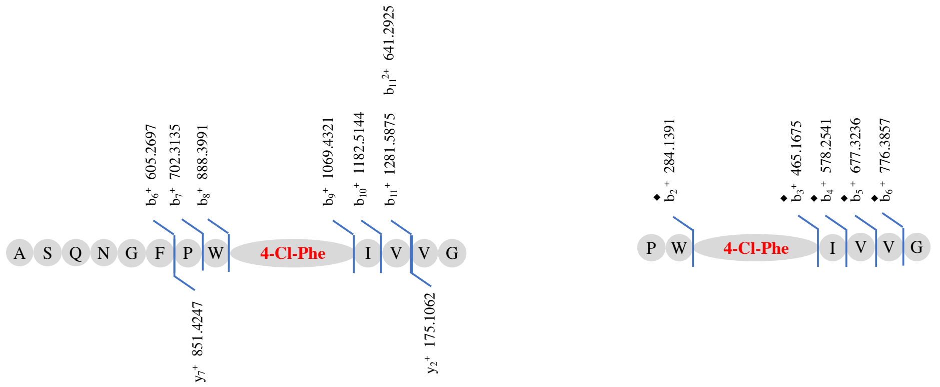

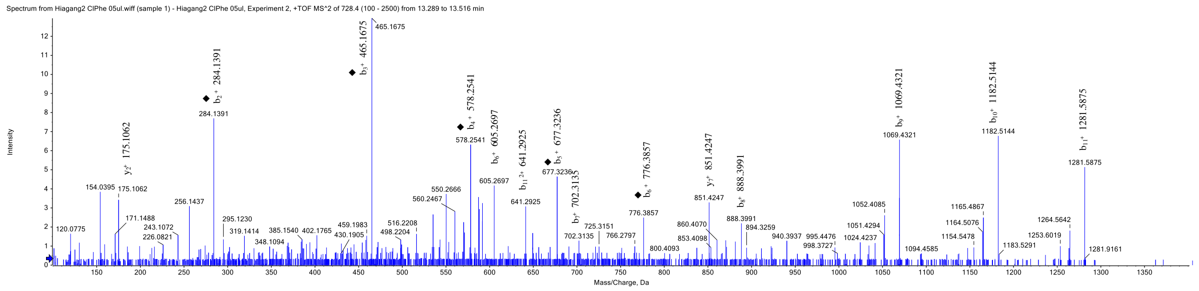

bo

fOphMA2-ΔC12-401-4I-Phe

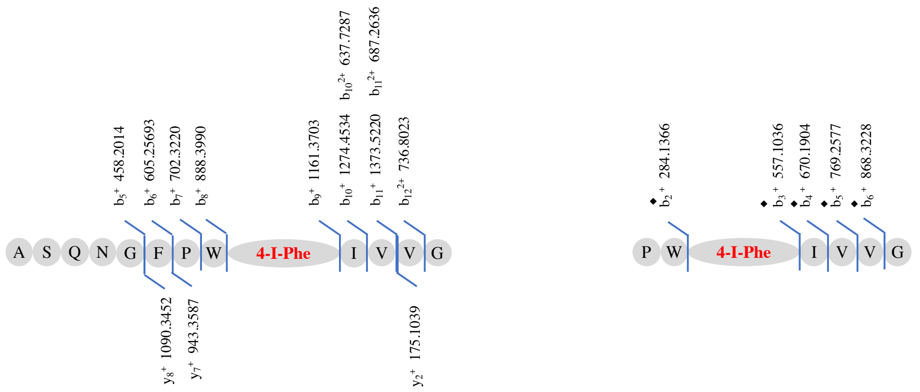

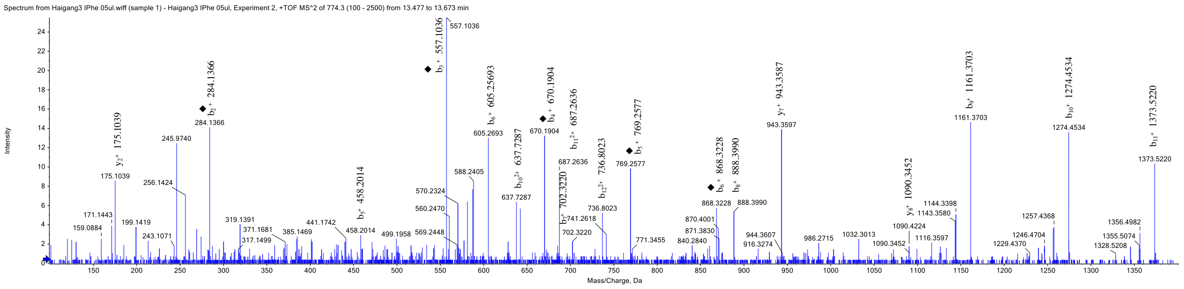

bp

fOphMA2-ΔC12-401-4NO_2-Phe

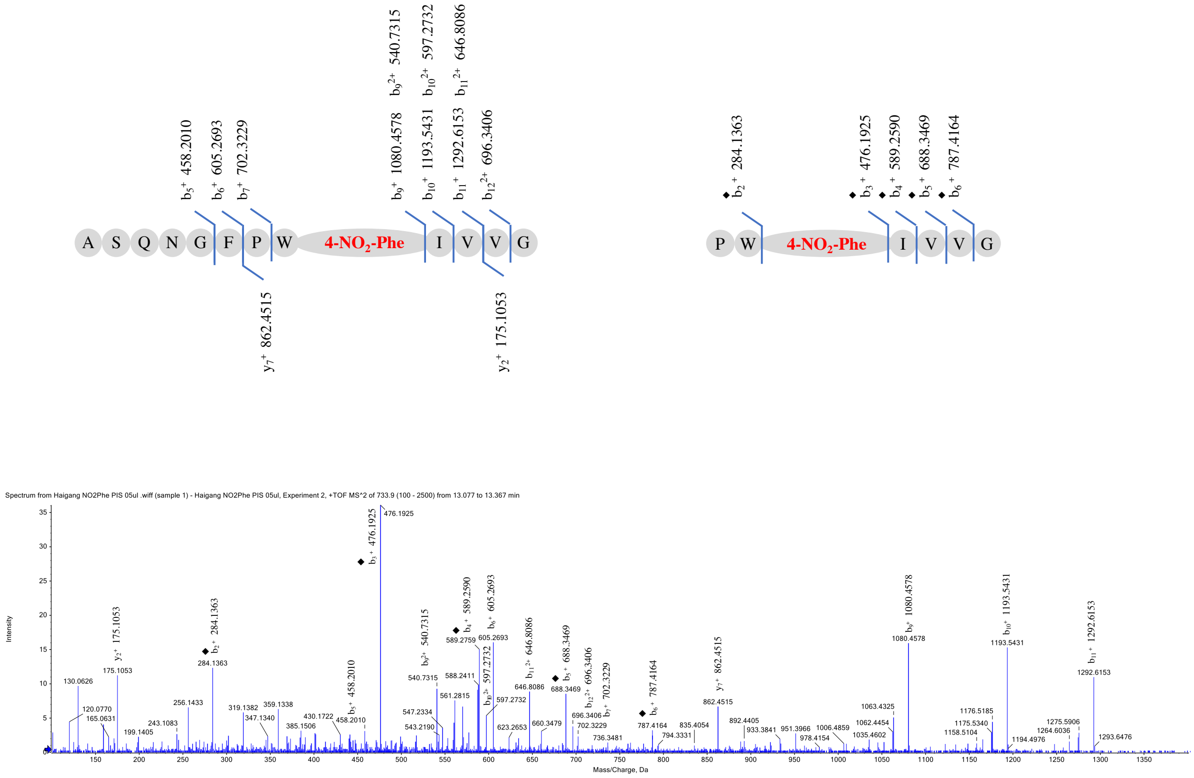

bq

fOphMA2-ΔC12-401Pen

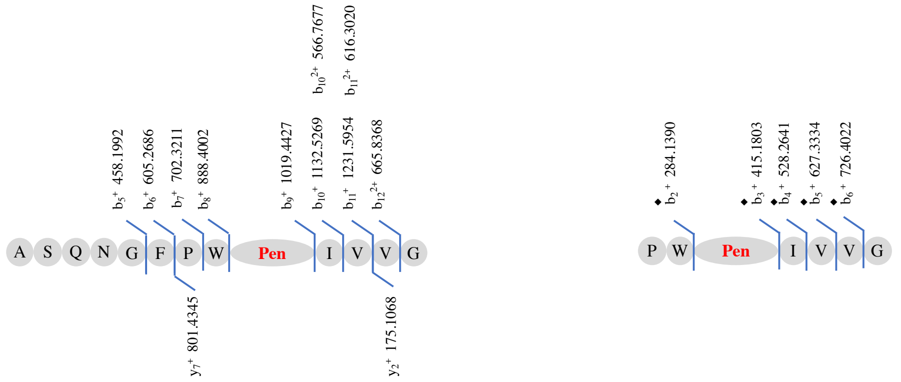

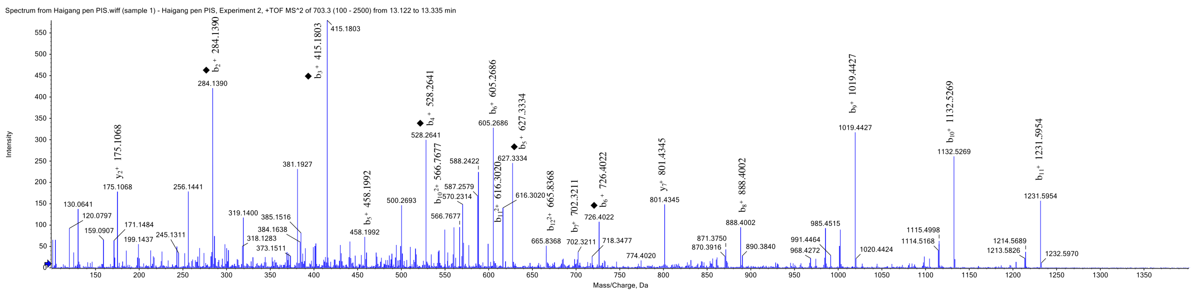

br

fOphMA2-ΔC12-401Pen

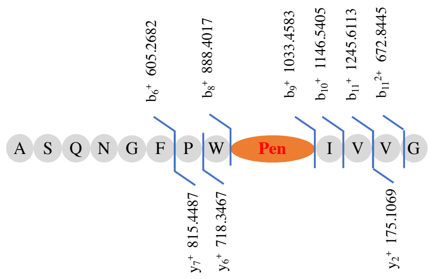

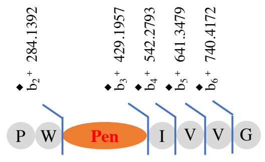

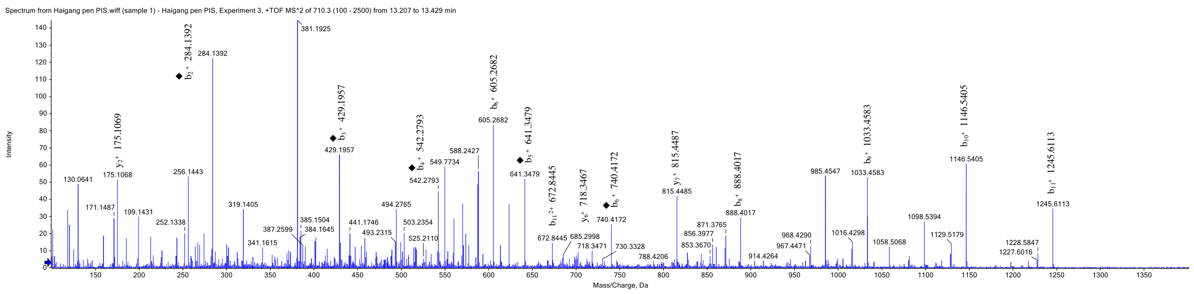

bs

fOphMA2-ΔC12-401Pen

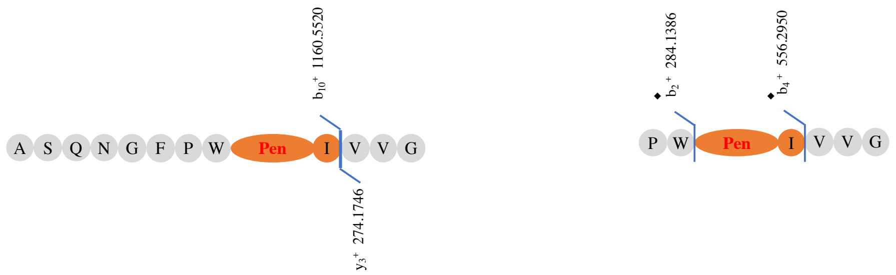

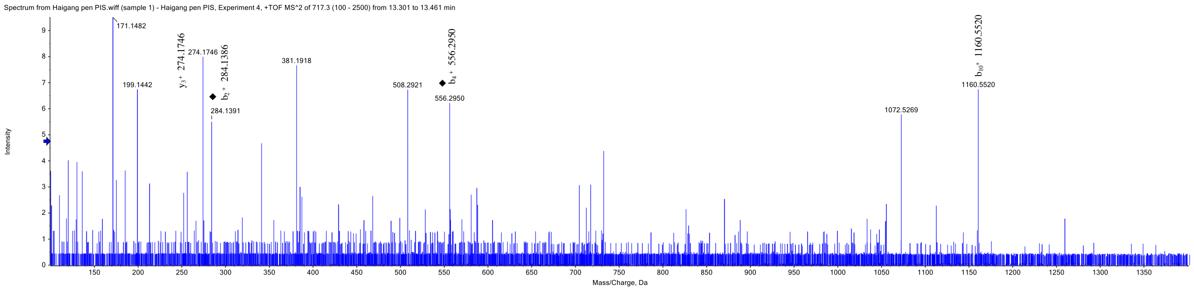

bt

fOphMA2-ΔC12-401Hse

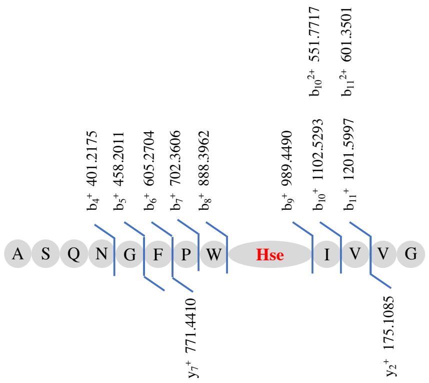

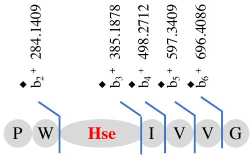

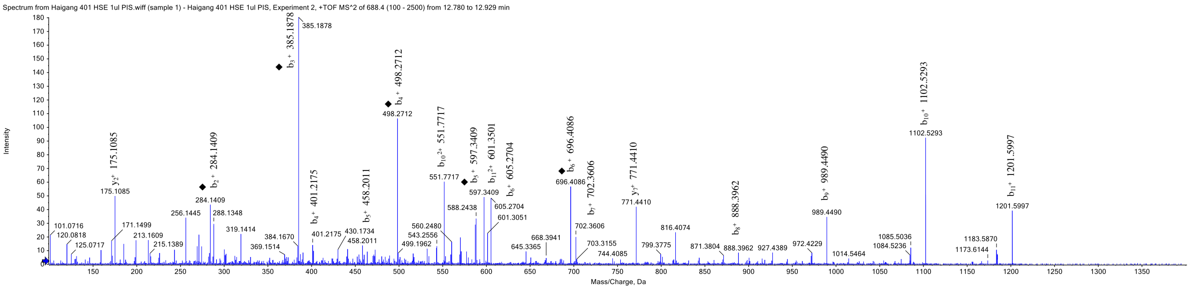

bu

fOphMA2-ΔC12-401Hse

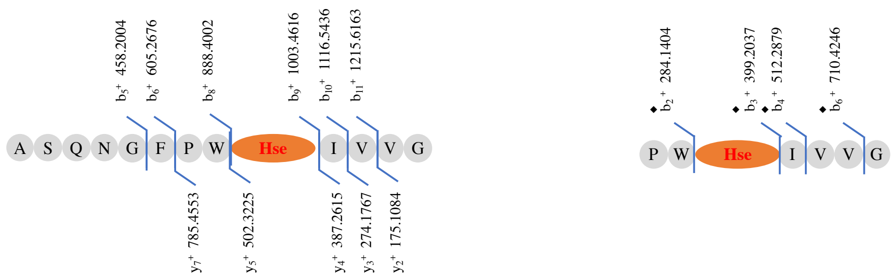

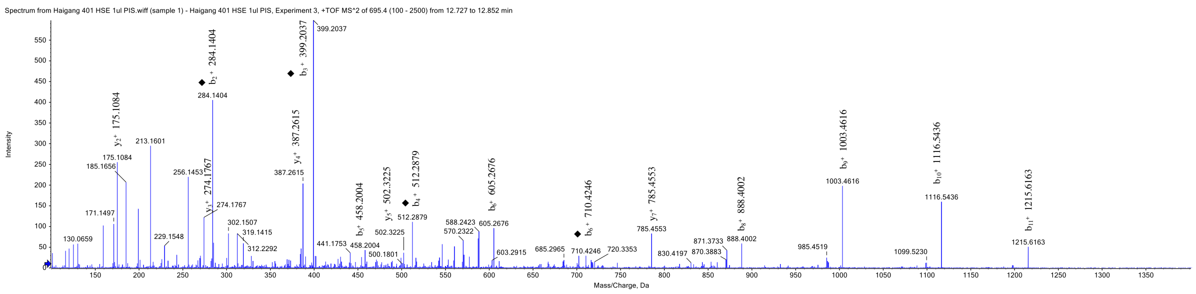

bv

fOphMA2-ΔC12-401Hse

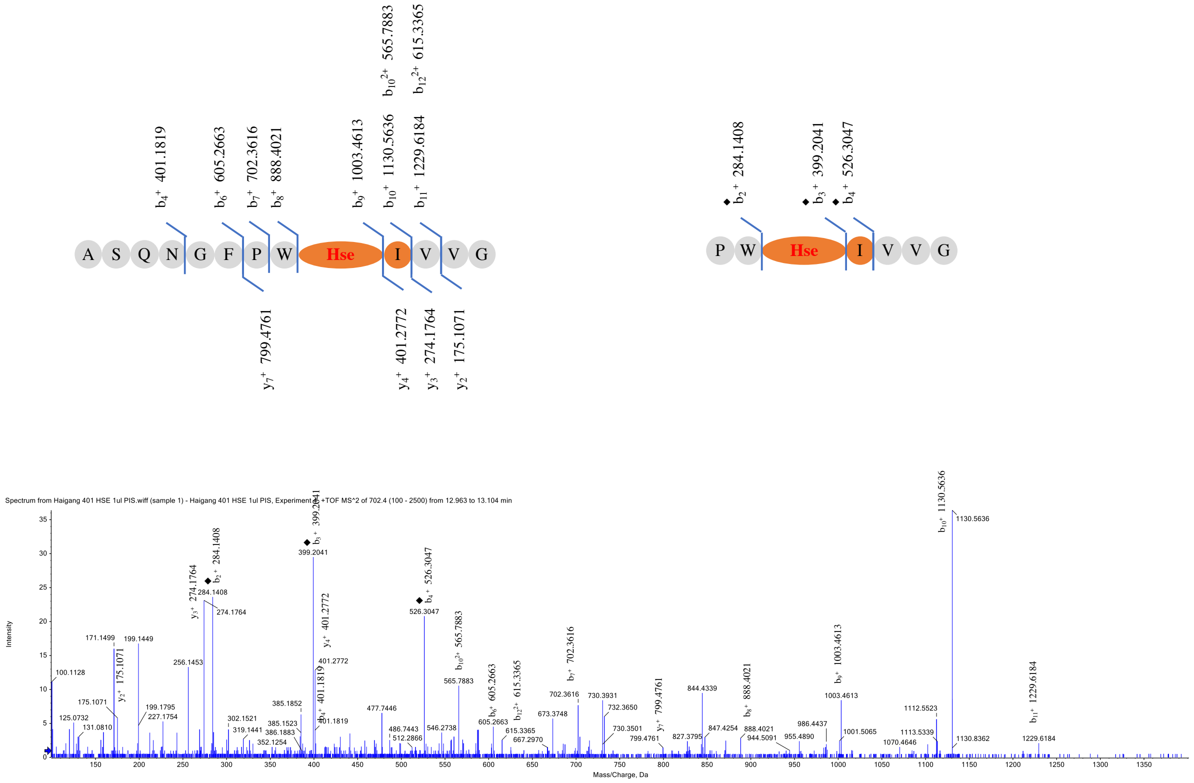

bw

fOphMA2-ΔC12-401Dab

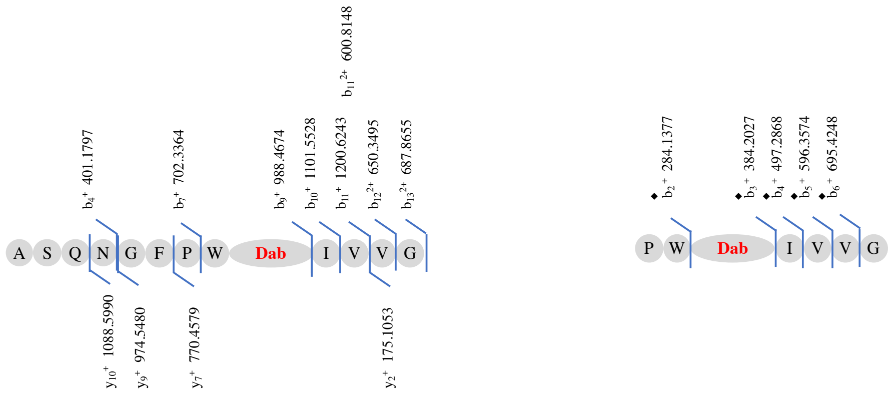

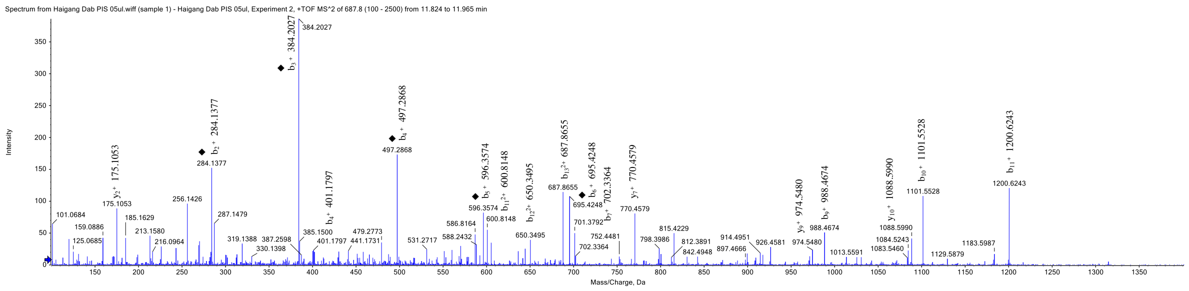

bx

fOphMA2-ΔC12-401Dab

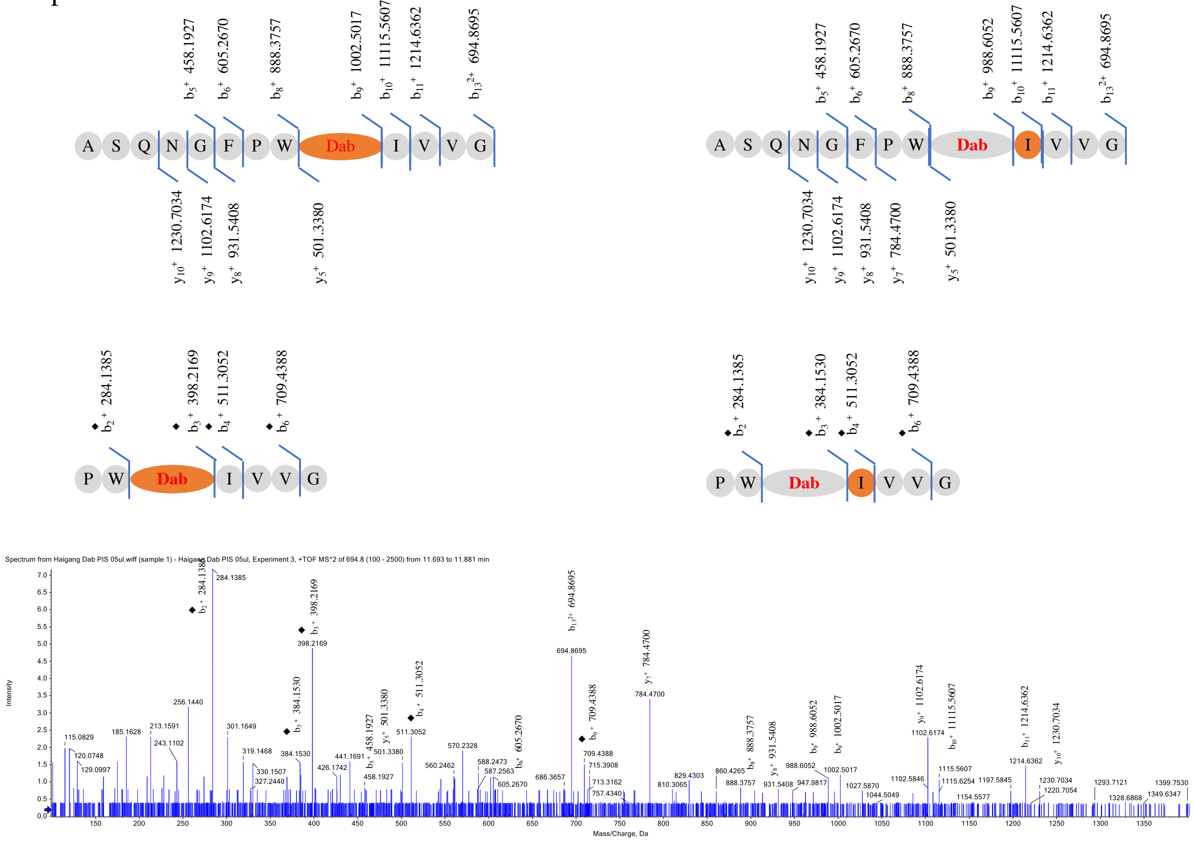

by

fOphMA2-ΔC12-401CysACM

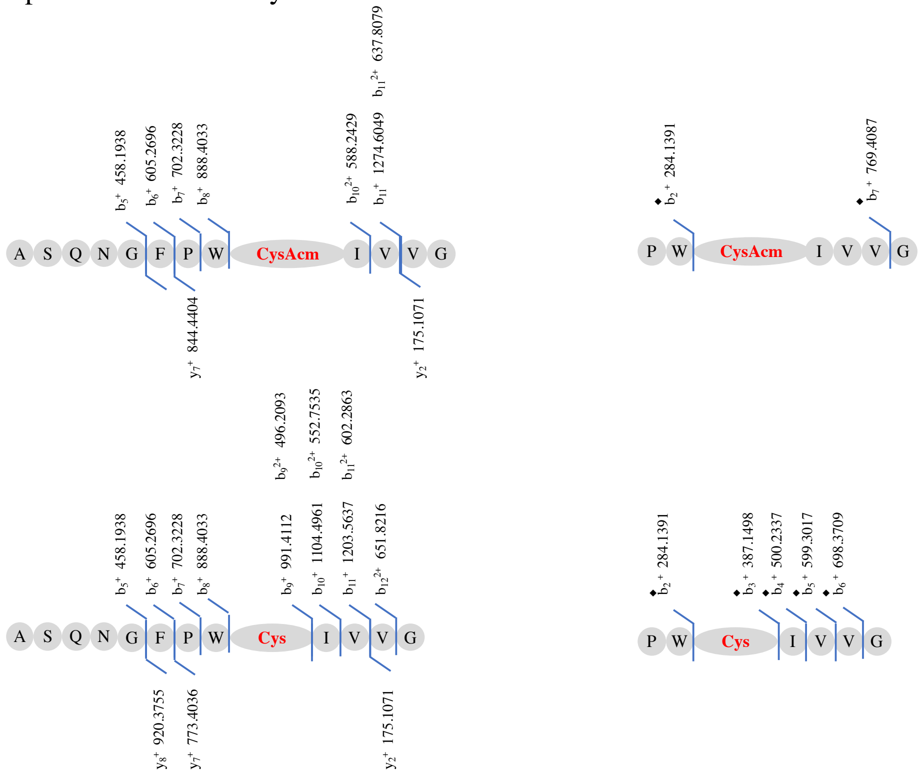

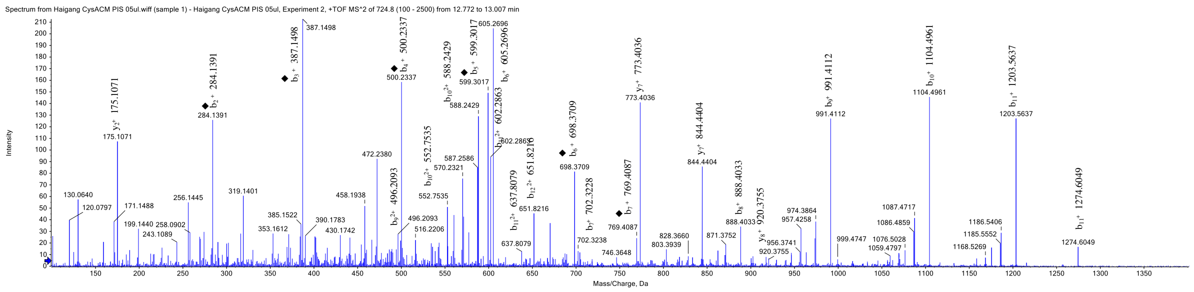

bz

fOphMA2-ΔC12-401CysACM

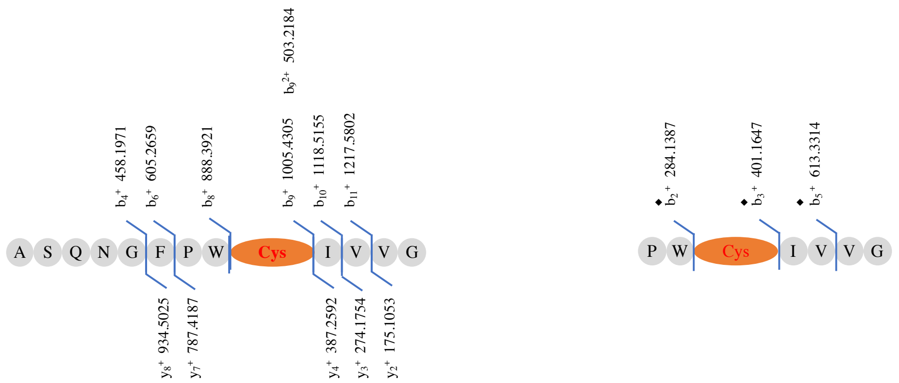

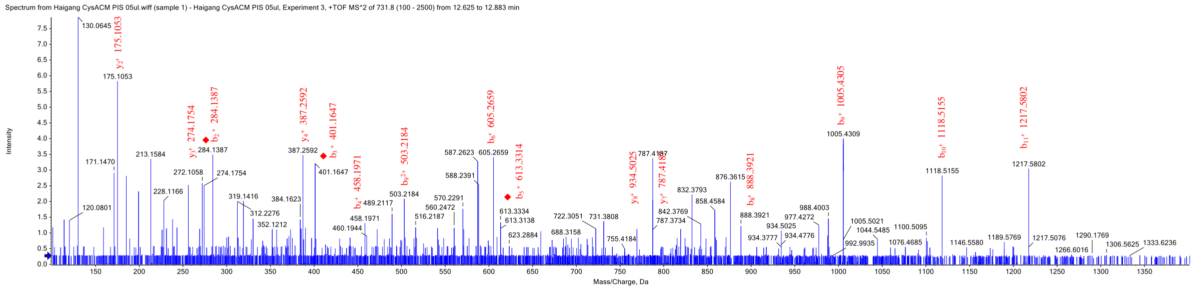

ca

fOphMA2-ΔC12-401Cit

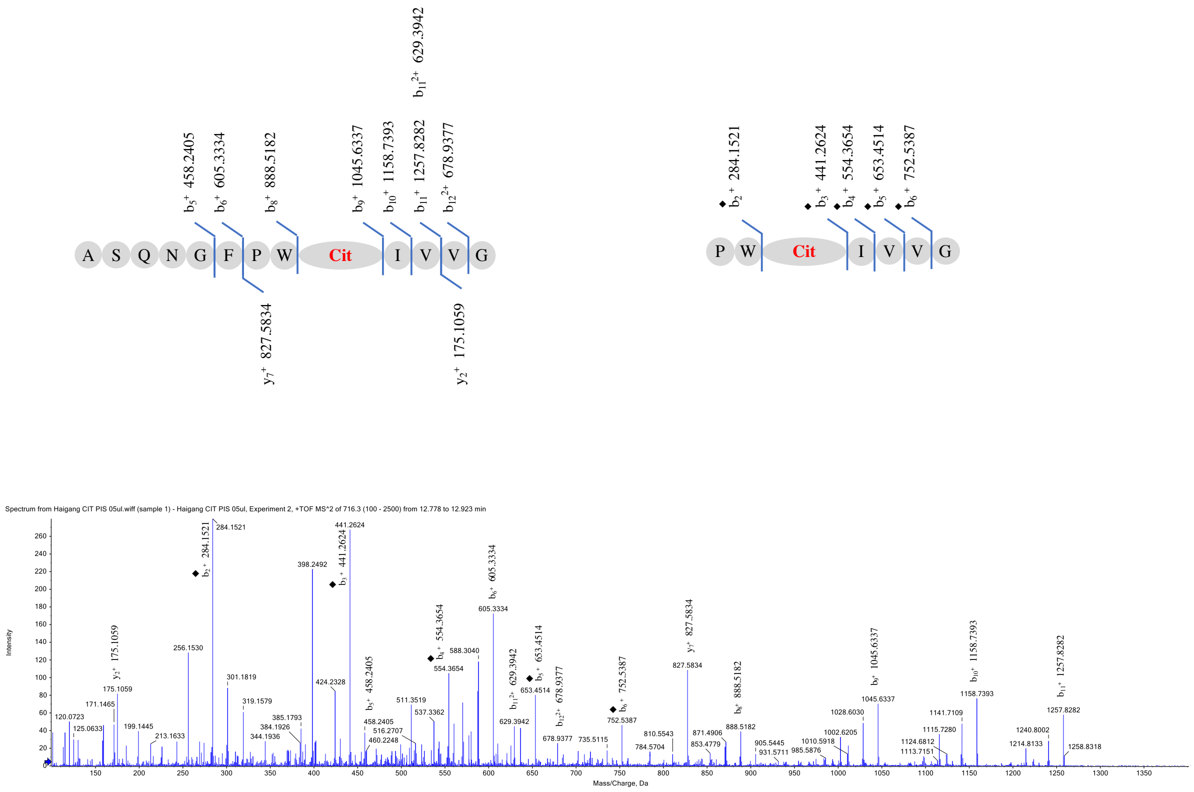

cb

fOphMA2-ΔC12-401Aib

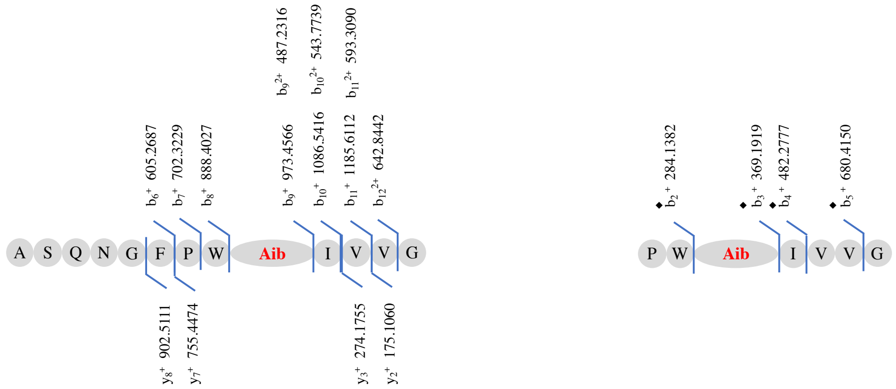

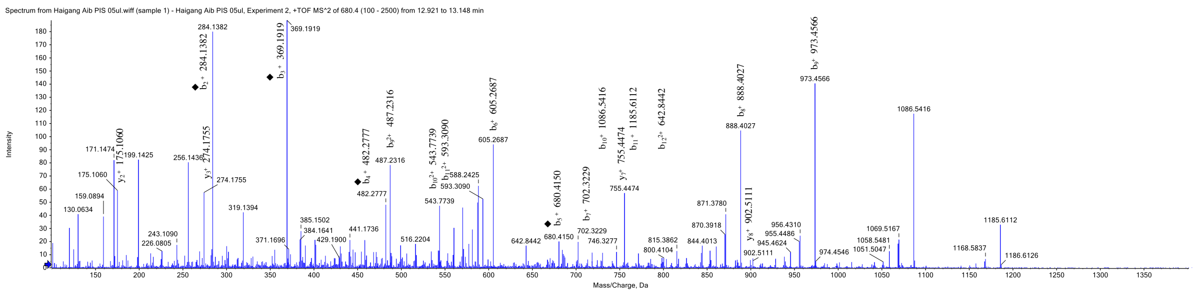

cc

fOphMA2-ΔC12-401-D-Ala

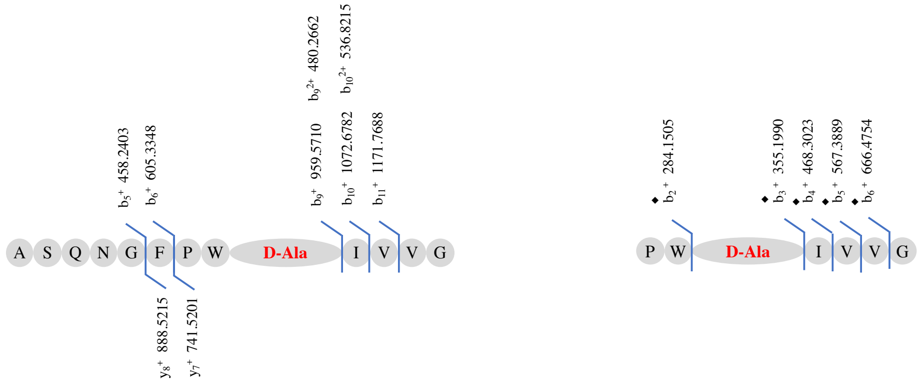

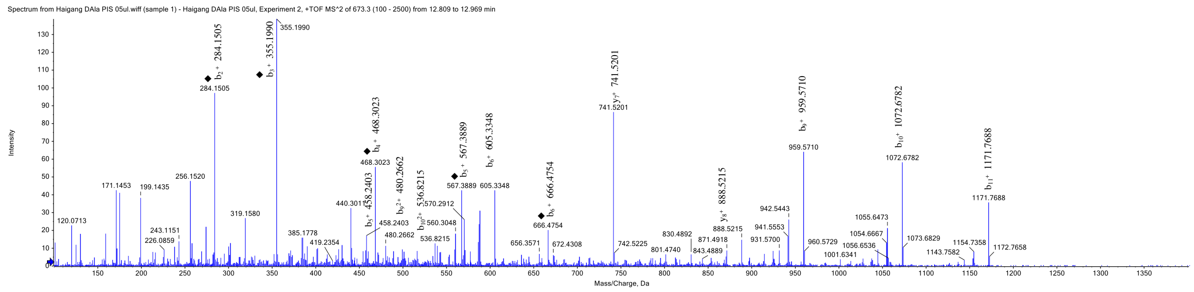

cd

fOphMA2-ΔC12-401-D-Val

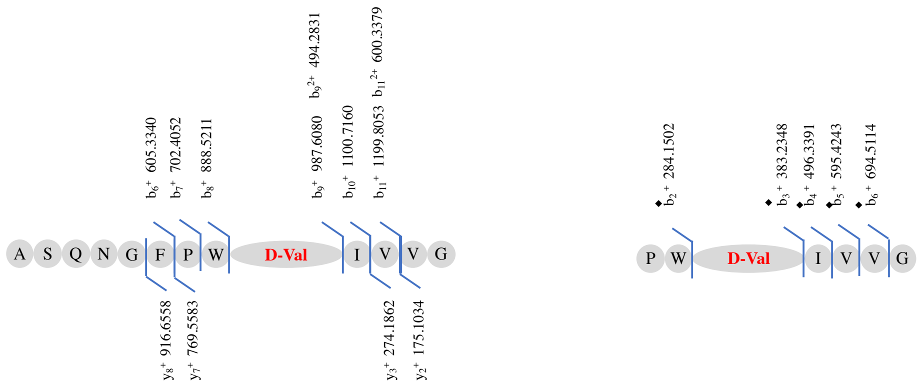

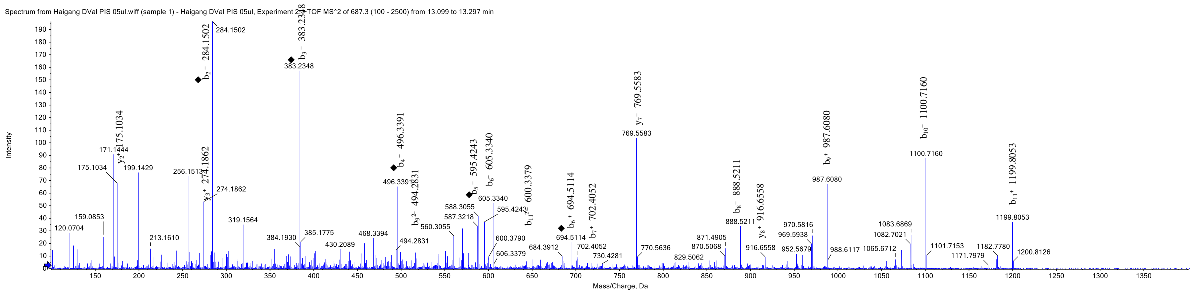

ce

fOphMA2-ΔC12-401-D-Thr

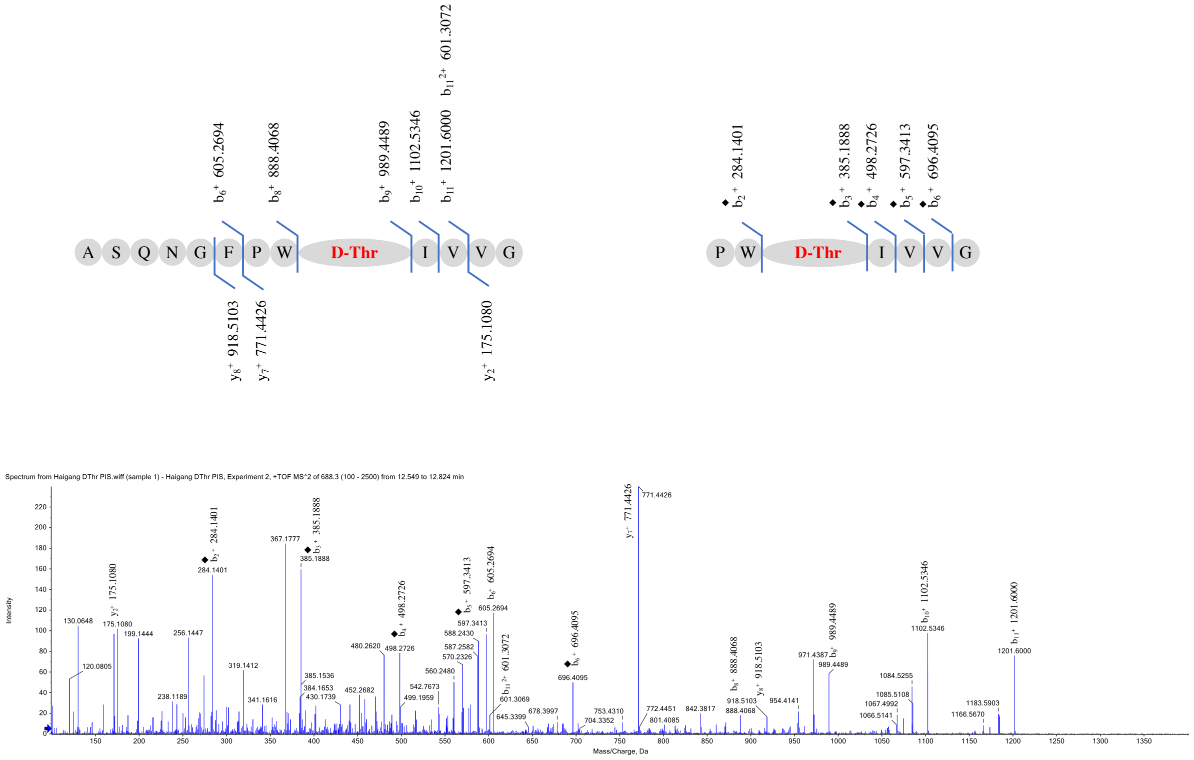

cf

fOphMA2-ΔC12-401-β-Ala

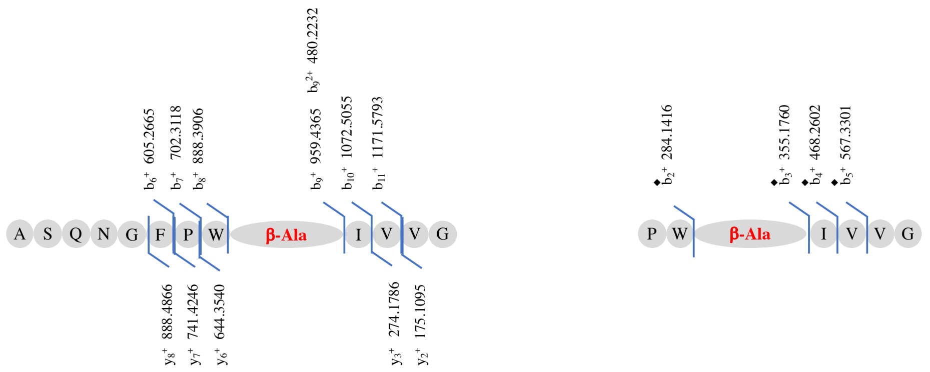

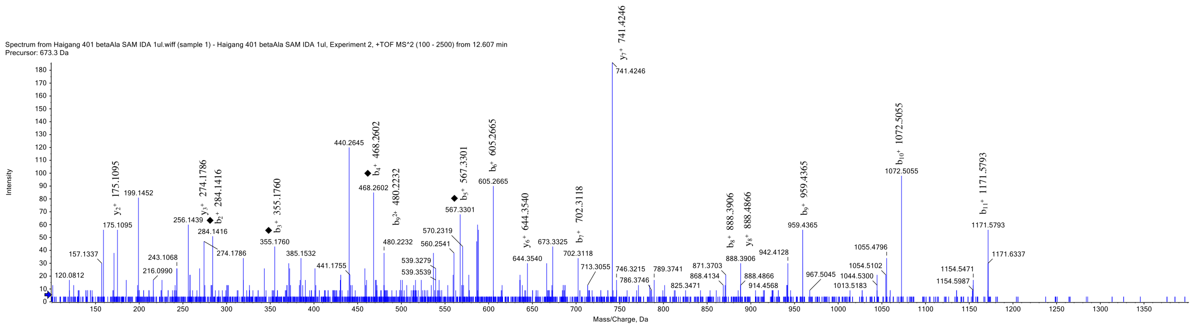

cg

fOphMA2-ΔC12-401-β-homoLeu

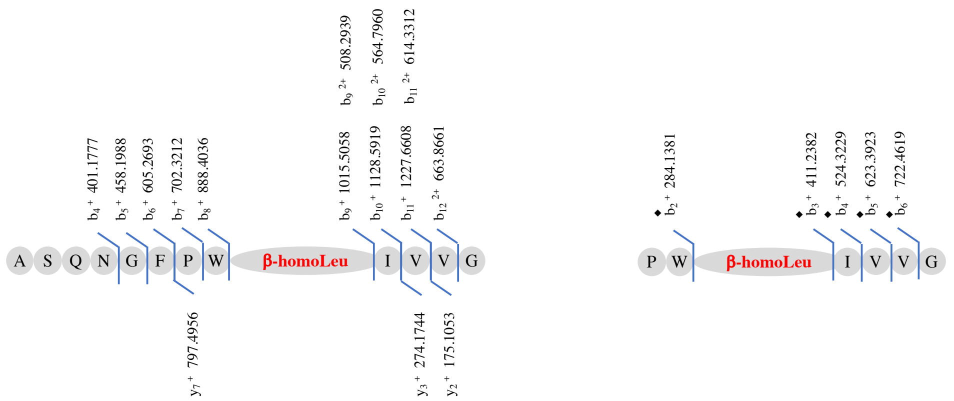

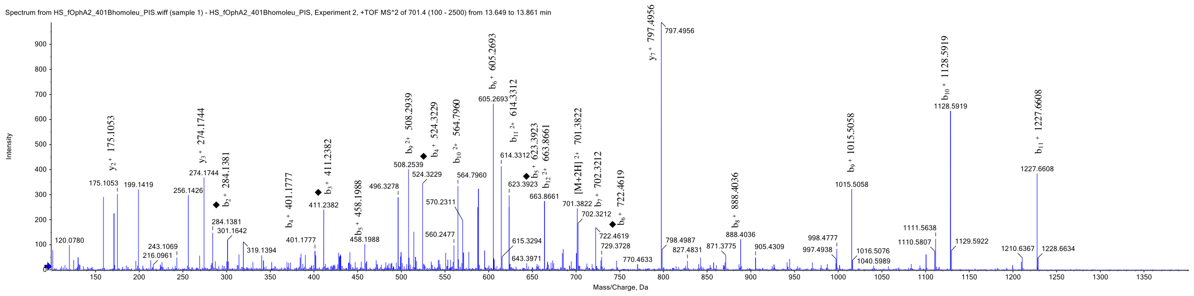

ch

fOphMA2-ΔC12-401-Gaba

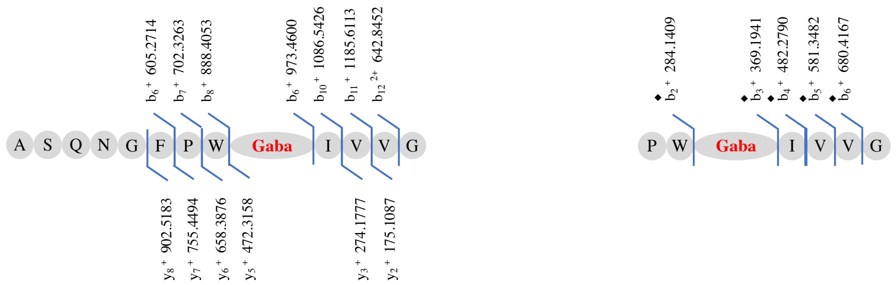

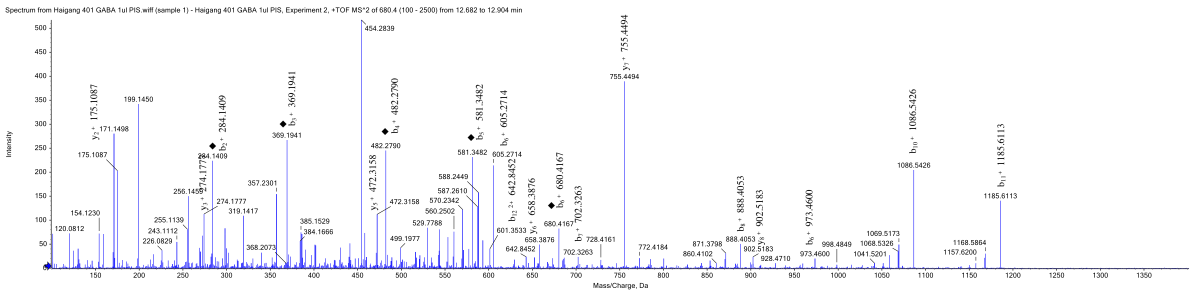

ci

fOphMA2-ΔC12-401-Gaba

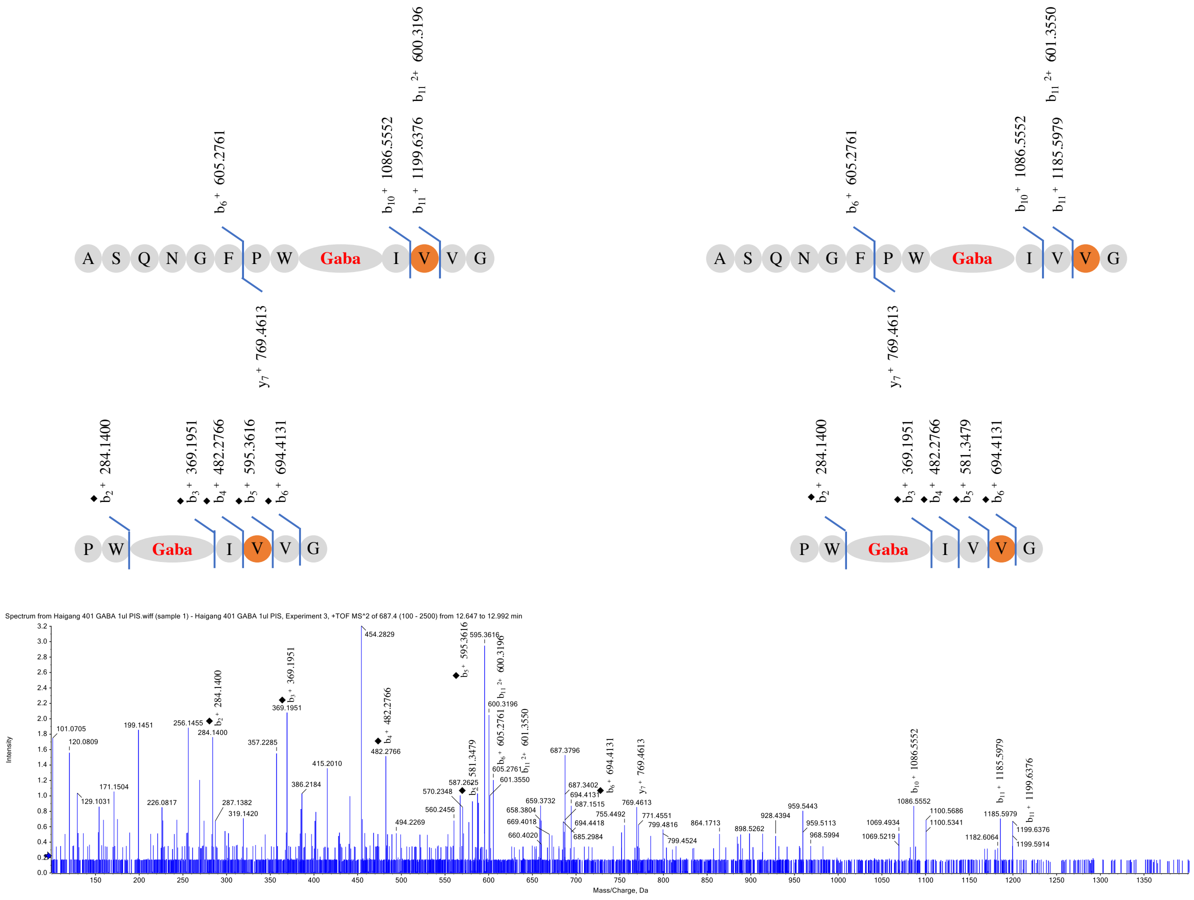

cj

fOphMA2-ΔC12-401-Ahx

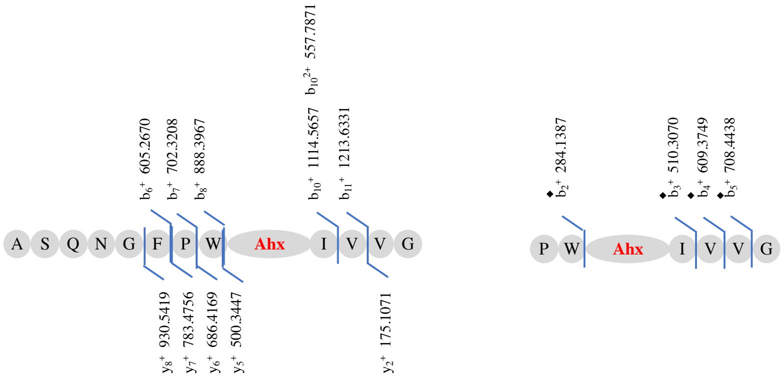

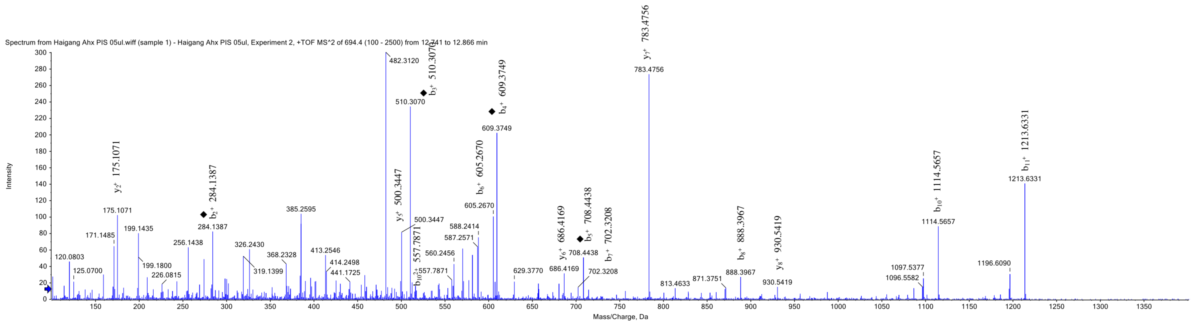

ck

fOphMA2-ΔC12-401-Ahx

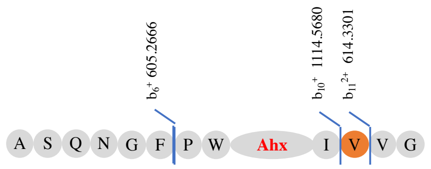

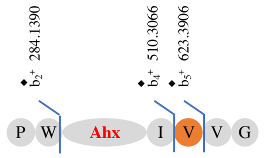

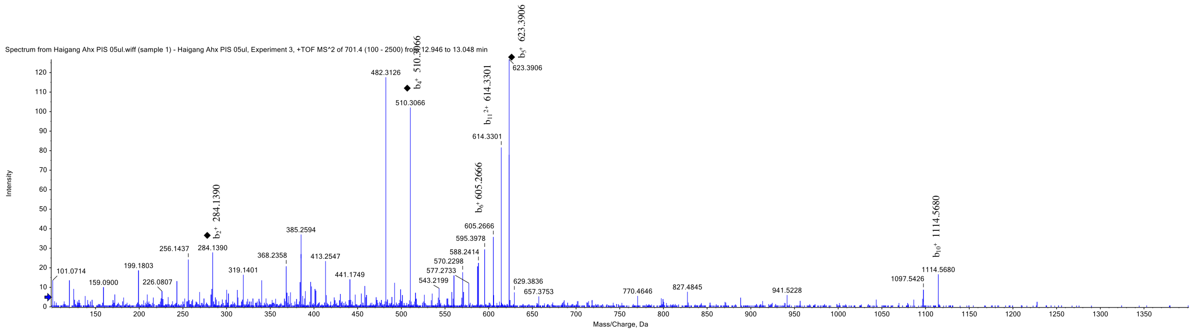

cl

fOphMA2-ΔC12-401-MiniPeg1

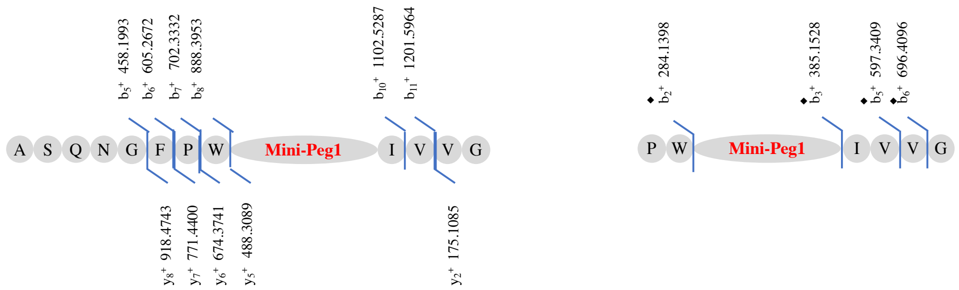

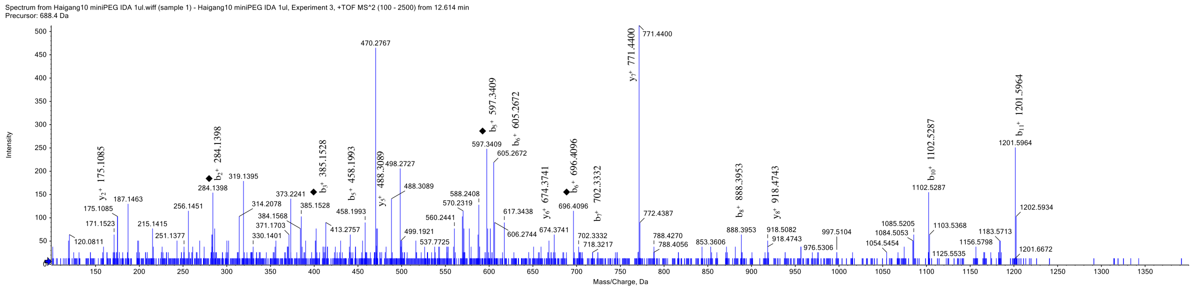

Table S1. Lists of all the peptides with various non-proteinogenic amino acids at position 401. The yield is based on the relative intensity (integrated peak areas) of individual species.

Peptide	Amino acid at 401 position	Abbreviation	Structure	Methylation at 401	Yield (mono-methylation)
**1**	Valine	**Val**	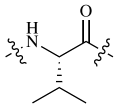	Yes	21%
**2**	Norvaline	**Nva**	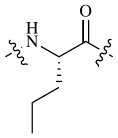	Yes	84%
**3**	Norleucine	**Nle**	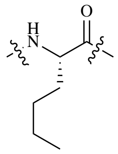	Yes	79%
**4**	Propargylglycine	**Pra**	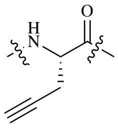	Yes	59%
**5**	β-cyclohexylalanine	**Cha**	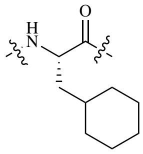	Yes	11%
**6**	α-cyclohexylglycine	**Chg**	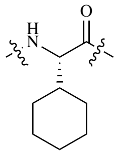	Yes	6%
**7**	Cyclopentylglycine	**Cpg**	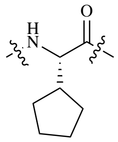	Yes	21%
**8**	2'-aza-phenylalanine	**2-Pal**	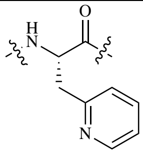	Yes	40%
**9**	3'-aza-phenylalanine	**3-Pal**	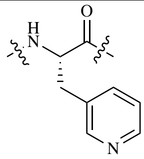	Yes	16%
**10**	4'-aza-phenylalanine	**4-Pal**	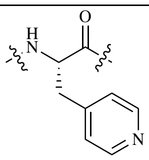	Yes	35%

**11**	Phenylglycine	**Phg**	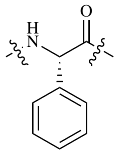	Yes	Detactable
**12**	4-fluro-phenylalanine	**4-F-Phe**	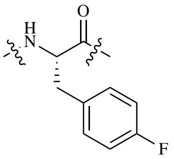	Yes	7%
**13**	4-chloro-phenylalanine	**4-Cl-Phe**	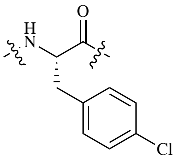	No	NA
**14**	4-iodo-phenylalanine	**4-I-Phe**	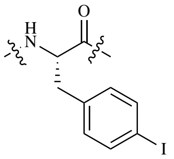	No	NA
**15**	4-nitro-phenylalanine	**4-NO_2-Phe**	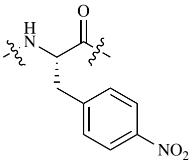	No	NA
**16**	Penicillamine	**Pen**	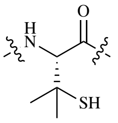	Yes	22%
**17**	Diaminobutyric acid	**Dab**	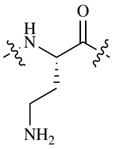	Yes	Detectable
**18**	Homoserine	**Hse**	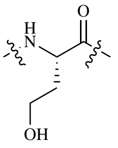	Yes	61% (Hse)<br><br>7% (Hse and Ile402)
**19**	S-acetaminomethyl-cysteine	**CysAcm**	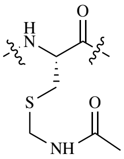	Yes	Detectable
**20**	Citrulline	**Cit**	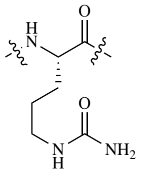	No	NA
**21**	β-alanine	**B-Ala**	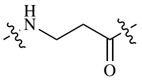	No	NA

**22**	β-homoleucine	**B-homoLeu**	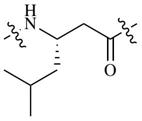	No	NA
**23**	γ-aminobutyric acid	**Gaba**	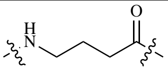	No	Detectable<br><br>(at Val403 or Val404)
**24**	6-aminohexanoic acid	**Ahx**	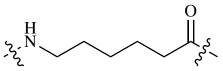	No	18%<br><br>(at Val403)
**25**	(2-aminoethoxy)-acetic acid	**Mini-Peg1**	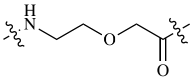	No	NA
**26**	D-alanine	**D-Ala**	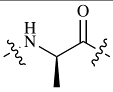	No	NA
**27**	D-valine	**D-Val**	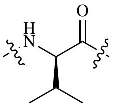	No	NA
**28**	D-threonine	**D-Thr**	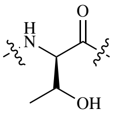	No	NA
**29**	α-aminoisobutyric acid	**Aib**	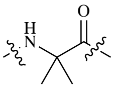	No	NA
**30**	Valine<br><br>(**CEEASQNGFPWVIVV GVIG**)	**Val**	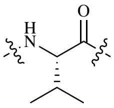	No<br><br>(ligation unsuccessful)	NA

References

[1] H. Liu, J. H. Naismith, *BMC Biotechnol.* **2008**, *8*, 91.
